# Recruitment of toxin-like proteins with ancestral venom function supports endoparasitic lifestyles of Myxozoa

**DOI:** 10.7717/peerj.11208

**Published:** 2021-04-26

**Authors:** Ashlie Hartigan, Adrian Jaimes-Becerra, Beth Okamura, Liam B. Doonan, Malcolm Ward, Antonio C. Marques, Paul F. Long

**Affiliations:** 1 Department of Life Sciences, Natural History Museum, London, United Kingdom; 2 Faculty of Life Sciences & Medicine, King’s College London, University of London, London, United Kingdom; 3 Departamento de Zoologia, Instituto de Biociências, Universidade de São Paulo, São Paulo, São Paulo, Brazil; 4 Aulesa Biosciences Ltd, Shefford, Bedfordshire, United Kingdom; 5 Faculdade de Ciências Farmacêuticas, Universidade de São Paulo, São Paulo, São Paulo, Brazil

**Keywords:** Venom, Cnidaria, *Buddenbrockia plumatellae*, *Polypodium hydriforme*, Toxin divergence, Secretion

## Abstract

Cnidarians are the oldest lineage of venomous animals and use nematocysts to discharge toxins. Whether venom toxins have been recruited to support parasitic lifestyles in the Endocnidozoa (Myxozoa + *Polypodium*) is, however, unknown. To examine this issue we variously employed transcriptomic, proteomic, associated molecular phylogenies, and localisation studies on representative primitive and derived myxozoans (Malacosporea and Myxosporea, respectively), *Polypodium hydriforme*, and the free-living staurozoan *Calvadosia cruxmelitensis*. Our transcriptomics and proteomics analyses provide evidence for expression and translation of venom toxin homologs in myxozoans. Phylogenetic placement of Kunitz type serine protease inhibitors and phospholipase A2 enzymes reveals modification of toxins inherited from ancestral free-living cnidarian toxins, and that venom diversity is reduced in myxozoans concordant with their reduced genome sizes. Various phylogenetic analyses of the Kunitz-type toxin family in Endocnidozoa suggested lineage-specific gene duplications, which offers a possible mechanism for enhancing toxin diversification. Toxin localisation in the malacosporean *Buddenbrockia plumatellae* substantiates toxin translation and thus illustrates a repurposing of toxin function for endoparasite development and interactions with hosts, rather than for prey capture or defence. Whether myxozoan venom candidates are expressed in transmission stages (e.g. in nematocysts or secretory vesicles) requires further investigation.

## Introduction

Venoms, by definition, are compounds delivered by injection for prey capture, defence and reproduction (Fry et al., 2009; Casewell et al., 2013; Schendel et al., 2019). Typically, they comprise complex mixtures of peptides and proteins, colloquially known as toxins, which variously act to kill or disrupt functions. Most research focuses on selected venoms of potential importance for human health–a bias that may highly compromise understanding of venom diversity. Indeed, wide-ranging sampling reveals an enormous and previously unappreciated diversity of other peptide and protein venom components such as peptidase inhibitors that unlike toxins, do not cause damage directly, but nevertheless still disrupt physiological functions (Jenner et al., 2019; Casewell et al., 2019; Lu et al., 2020). However, in its extensive focus on free-living animals, venom research may overlook major avenues of toxin diversification. In particular, venom components may have suited transitions to parasitism with venom traits being co-opted and further modified in support of parasitic lifestyles. In view of the multiple transitions to parasitism within Metazoa (Weinstein & Kuris, 2016), such a scenario is not far-fetched, but the deployment of venom cocktails by parasites may not always involve injection by delivery systems.

Cnidarians are arguably the most basal venomous extant metazoan (Starcevic & Long, 2013). Their toxins are produced by the Golgi apparatus of ‘stinging cells’ (cnidocytes) and are then transported to specialised organelles (nematocysts). Venom is inoculated from nematocysts into prey following the explosive eversion of a hollow tubule that punctures prey surfaces (Fautin, 2009). As in other venomous animals, the major toxic activities of cnidarian venoms involve enzymatic, neurotoxic, and cytolytic actions. Cnidarian venoms can be as complex in composition as those of insects, gastropods and elapid snakes (Jaimes-Becerra et al., 2019; Šuput, 2009; Badré, 2014; Mariottini et al., 2015).

**Figure 1:**
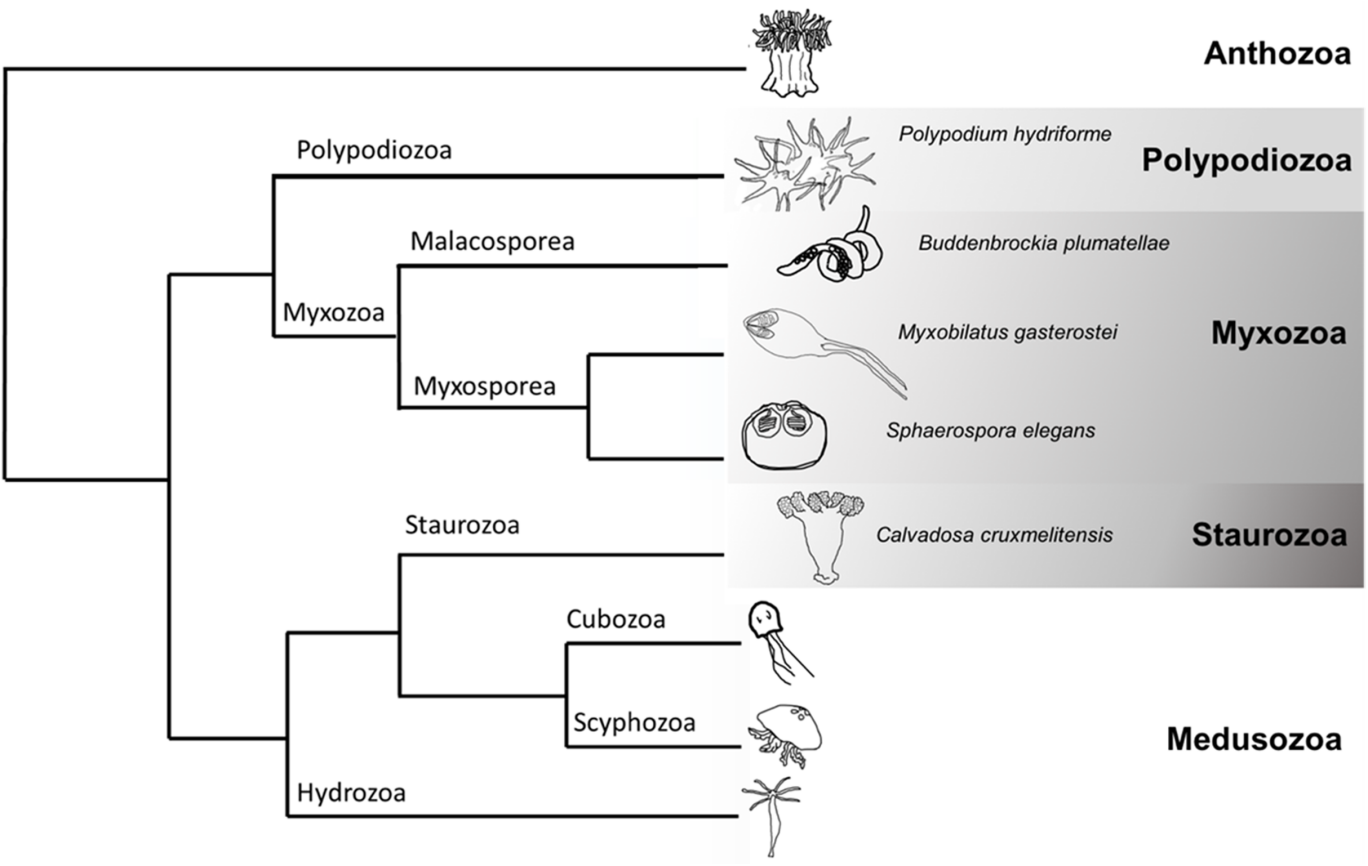
Cladogram of Cnidaria displaying some major lineages including placement of species used in this study (greyscale clades). Phylogenetic tree representing some of the major clades of Cnidaria (based (Chang et al., 2015; Kayal et al., 2018)). The species sampled in this venomics study were the myxozoans *Buddenbrockia plumatellae, Myxobilatus gasterostei* and *Sphaerospora elegans*, the parasitic and free-living *Polypodium hydriforme* and the free-living staurozoan *Calvadosia cruxmelitensis*.

Although widely recognised as iconic residents of our seas (e.g. corals, jellyfish, and sea anemones), cnidarians also include a large radiation of endoparasites, the Myxozoa (Fig. 1). Molecular phylogenetic analyses place the monotypic *Polypodium hydriforme* as sister to Myxozoa, forming the Endocnidozoa (Chang et al., 2015; Kayal et al., 2018) (Fig. 1). Myxozoans comprise some 20% of currently recognised cnidarian species (Okamura, Hartigan & Naldoni, 2018) and exploit vertebrate and invertebrate hosts in complex life cycles. Extremely rapid rates of molecular evolution, morphological simplification and miniaturisation long precluded higher-level placement of myxozoans (Okamura, Gruhl & Reft, 2015) and may be associated with their greatly reduced genome sizes (Chang et al., 2015). Myxozoans are comprised of the sister taxa, Malacosporea and Myxosporea. The former possess tissues and develop into active vermiform ‘myxoworms’ (e.g. *Buddenbrockia plumatellae*) and inert sacs (e.g. *Tetracapsuloides bryosalmonae*). Myxosporeans are more derived having lost tissues and developing as plasmodia and pseudoplasmodia (Okamura, Gruhl & Bartholomew, 2015). Myxozoans exploit marine, freshwater and terrestrial hosts. Transmission is achieved by water-borne multicellular spores. Nematocysts (traditionally referred to as polar capsules in myxozoans, henceforth referred to as nematocysts (Americus et al., 2020)) within spores effect attachment to hosts and amoeboid cells then penetrate host surfaces and migrate to infection sites. The life cycle of *P. hydriforme* entails a parasitic larval stage in eggs of paddlefish and sturgeon and a free-living, actively feeding adult stage (Raikova, 1994).

Could venoms deployed by free-living ancestors be used to support parasitic lifestyles in endocnidozoans? There is currently no compelling evidence. Foox et al. (2015) identified proteins similar to cnidarian toxins in the transcriptome of *Myxobolus pendula* but did not address whether such putative toxins were distinct from those with non-toxic physiological functions. Proteomic analysis of isolated polar capsules from the myxozoan *Ceratonova shasta* found minimal evidence for proteins similar to toxins of cnidarians or other venomous animals (a toxin-like ShK domain was identified in one peptide sequence, but this was later discounted (Piriatinskiy et al., 2017)). However, nematocysts and filament function have been shown to be highly variable in myxozoans (Ben-David et al., 2016). The apparent loss of venom toxins in nematocysts of *C. shasta* could be attributed to a structural adaptation that facilitates host attachment rather than toxin delivery.

Here we tackle the question of whether endoparasites employ venom toxins inherited from their free-living ancestors by presenting comparative transcriptomics, targeted proteomics and further expression studies on cnidarian taxa with entirely parasitic life cycles (*Buddenbrockia plumatellae* and the myxosporeans *Myxobilatus gasterostei* and *Sphaerospora elegans*), entirely free-living life cycles (the staurozoan *Calvadosia cruxmelitensis*), and a life cycle containing both parasitic and free-living stages (*P. hydriforme*) (Fig. 1). Our rationale is that expression of venom-candidate proteins in endoparasites provides evidence for repurposing of toxins from their ancestral functions of prey capture and defence. In support of this study, we developed a custom pipeline to increase rigour in screening sequences and identifying putative toxins in our datasets. *M. gasterostei* and *S. elegans* were collected as mixed infections representing various stages of development (plasmodia with and without spores) from fish kidneys. Because their transcriptomic and proteomic data could not be deconvoluted we hereafter refer to ‘mixed myxosporean’ datasets. Multiple stages of development (mature and early stages with and without spores, respectively) also characterised the pooled *B. plumatellae* myxoworms collected from bryozoan hosts.

Our collective approaches enable us to test the following hypotheses: (H1) venom toxins have been conserved and repurposed in endoparasitic cnidarians; (H2) toxins of endoparasitic cnidarians are more divergent from homologs in free-living relatives; and (H3) toxin diversity will be reduced in myxozoans in keeping with the general trend for reduction in genome size in this group. By characterising toxins in myxozoans our aim is to expand the overall appreciation of how toxins have evolved in cnidarians as a whole and to address whether toxins may have been repurposed in animals with parasitic lifestyles.

## Materials and Methods

Extended methods are available in the Supplemental Methods.

Transcriptomics: the material used to isolate RNA was as follows: 50 stickleback kidneys of varying sizes with mixed myxosporean infections (*Myxobilatus gasterostei* and *Sphaerospora elegans* samples were not separated and hence treated and interpreted as a mixed dataset); approximately 500 *B. plumatellae* myxoworms from bryozoan host (*Plumatella emarginata*); 20 stolons of *P. hydriforme;* secondary tentacle clusters from each of two arms of 20 individual *C. cruxmelitensis*. RNA was isolated from all material using the same protocol involving homogenisation in TRIzol Reagent followed by biphasic extraction with alcohol and MagMax magnetic beads. RNA library preparation used a NEBNext Poly(A) mRNA Magnetic Isolation Module and fragments with an average size of 300 bp were sequenced on an Illumina HiSeq platform.

Removal of host contaminating sequences: see Supplemental Methods for detailed host filtering pipeline. In summary, to filter the myxozoan and *P. hydriforme* raw transcriptomic reads from potentially contaminating fish sequences, the raw reads were mapped to the cDNA and ncRNA sequences of *G. aculeatus* (https://www.ensembl.org/Gasterosteus_aculeatus/Info/Index) (Jones et al., 2012), *Rutilus rutilus* transcriptome GEBE00000000.1 and sequences from the *P. spathula* lateral-line transcriptome (Modrell et al., 2017) (bio-project PRJNA357629). Paired reads that did not align to the host datasets were used for transcriptome assembly. Putative fish sequences were also removed after assembly using BLASTn (e value 1e−10, sequence similarity/alignment 80%). Additionally, a draft transcriptome of *Plumatella vaihiriae* was constructed to remove invertebrate host sequences from the *Buddenbrockia* transcriptome using the Alien_Index pipeline (https://github.com/josephryan/alien_index, see Supplemental Methods).

Transcriptome assembly: each of the four transcriptomes were assembled with Trinity v2.6.6 using default settings (Haas et al., 2013). Assembled transcriptomes were translated with OrfPredictor v3.0 (Min et al., 2005) using default settings. Sequences >30 amino acids in length were retained. Gene completeness for each of the now non-redundant transcriptomes was assessed with BUSCO v3.0.2 using the metazoan odb nine sequence database (http://busco.ezlab.org/) (Simão et al., 2015).

Proteomics: soluble proteins were extracted by homogenisation and precipitated using methanol, chloroform and water from: 100 kidneys infected with the mixed myxosporeans, approximately 500 *B. plumatellae* myxoworms, 20 individuals of *P. hydriforme* and the tentacle clusters from 20 individuals of *C. cruxmelitensis*. A total of 300 µg of protein from each sample was reduced and alkylated before in-solution digestion was performed using trypsin. Each sample was separated into 12 peptide fractions and analysed by LC-MS/MS using an Ultimate 3,000 nano-LC system in line with an Orbitrap Velos mass spectrometer operated in data-dependent acquisition mode.

Identification of putative toxin encoding transcripts and potential toxins from proteomic data: Translated non-redundant proteins from each transcriptome were matched with BLASTp (e-value 1e−05, bit score > 50) against the UniProt/Swiss-Prot database and a customised toxin dataset which consisted of the Tox-Prot database (Jungo et al., 2012) (downloaded 12/07/2017) supplemented with putative cnidarian toxin sequences reported in the literature but not deposited in either the UniProt/Swiss-Prot or Tox-Prot databases (Jungo et al., 2012; Brinkman et al., 2015; Macrander, Broe & Daly, 2015; Gacesa et al., 2015; Ponce et al., 2016). A transcript was deemed to be a potential toxin sequence if the custom toxin database BLAST hit had a bit score > the corresponding UniProt hit. For each set of putative toxins, conserved domains were predicted using InterProScan5 (Jones et al., 2014), and these predictions were used to assign sequences to the most likely toxin protein family. The assignments were then checked by manual search of the scientific literature to validate the putative toxin function. Unique MS/MS spectral events were visualised using PEAKS Studio software v8.5 (Zhang et al., 2012). Peptides for each animal were back translated and paired against the corresponding non-redundant transcriptome using the PEAKS proprietary matching algorithm. Spectra for peptide- transcriptome matches were manually validated for unbroken series of overlapping b-type and y-type sequence specific fragments ions. Alignments of peptide-spectral matches to putative toxins previously annotated from the transcriptome are given in Fig. S1.

Comparative toxin profiles: to compare toxin diversities a Venn diagram was constructed using InteractiVenn (Heberle et al., 2015) from a data matrix of presence (1) and absence (0) of putative toxin protein families identified in this study and in 11 previously published venom proteomes (Jaimes-Becerra et al., 2019) to reflect total similarity. This matrix is shown in Table S1.

Molecular phylogenetic analyses: validated protein sequences previously identified as venom Kunitz-type toxins and phospholipase A2 (PLA2) from five taxa (sea anemones, insects, scorpions, spiders and snakes) with extensively studied venom as well as protein sequences of some non-venomous taxa were downloaded from the UniProt database and several published cnidarian transcriptomes (Zapata et al., 2015) (Tables S2 and S3). These sequences are provided in FASTA format together with the sequences identified in the transcriptomes of our study (Figs. S2 and S3). Multiple alignments of these sequences, together with homologs of potential venom Kunitz-type toxins and PLA2 sequences identified in this study, were constructed (Figs. S4 and S5). Phylogenetic analyses from these alignments were then carried out using maximum likelihood and Bayesian approaches. Comparison of the substitution rates between free-living cnidarians and endocnidozoans was conducted using the program RRTree version 1.1 (Robinson-Rechavi & Huchon, 2000).

Localisation of putative toxins in *B. plumatellae*: whole mounts of *B. plumatellae* myxoworms were stained using customised Rabbit polyclonal antibodies raised against three potential toxins that were identified in both the transcriptome and proteome data; a serine peptidase inhibitor, a C-type lectin and a CRiSP allergen (Vertebrate Antibodies Ltd., Aberdeen, UK). Specificity and sensitivity of the polyclonal antibodies against the proteins were measured by ELISA and are shown in Table S4. Localisation of the potential toxins was made after counter staining with an Alexafluor-conjugated anti-rabbit antibody using a Nikon Eclipse upright microscope with A1-Si confocal microscope.

Data deposition: raw reads for the transcriptomes generated in this study are available at NCBI Sequence Read Archive (SRA) as Bioproject number PRJNA576367. The mass spectrometry proteomics data have been deposited to the ProteomeXchange Consortium via the PRIDE (Perez-Riverol et al., 2019) partner repository with the dataset identifier PXD016306.

## Results

### Annotation and validation of potential toxin-encoding transcripts

**Figure 2:**
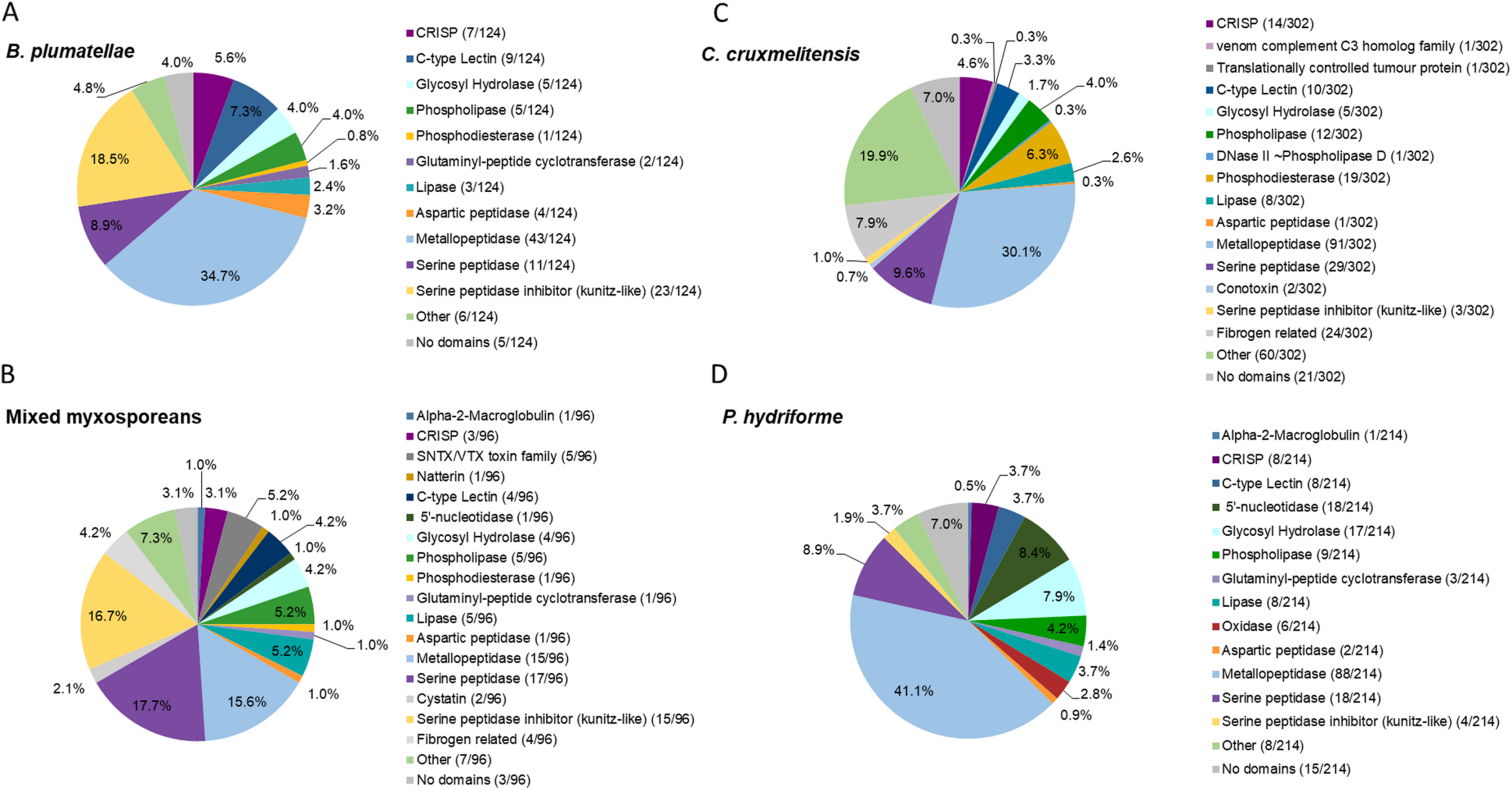
Comparative analysis of putative toxins identified from the transcriptomic datasets. Transcripts were identified as encoding for potential toxins in the transcriptomes of the myxozoans (A) *Buddenbrockia* *plumatellae* (*n* = 124) and (B) a mixed myxosporean dataset (*n* = 96 putative toxins) the free living cnidarian (C) *Calvadosia cruxmelitensis* (*n* = 302) and the free-living and parasitic (D) *Polypodium hydriforme* (*n* = 214). Toxins were identified from each transcriptome using a customised annotation pipeline based on known domain and sequence homology searching.

Summary statistics and host contamination results for the assembled transcriptomes are given in Table S5. All full-length coding regions were translated, and the predicted amino acid sequences (>30 amino acids in length) were compared to sequences in the UniProt/Swiss-Prot database and our customised toxin dataset (comprised of those in the Tox-Prot database supplemented with putative cnidarian toxin sequences not deposited in UniProt/Swiss-Prot or Tox-Prot databases; see Supplemental Methods). The putative toxins were screened for transcript redundancy and further manually validated by detecting recognised toxin domains and then grouped based on predicted functions (Fig. 2 and Tables S6–S9). A higher number of potential toxins were identified in the *C. cruxmelitensis* and *P. hydriforme* datasets in comparison to the myxozoans. Transcripts providing high-scoring BLAST matches to degradative enzymes (e.g. peptidases and lipases in putative toxin protein families) were most diverse in all four datasets, and toxins with predicted haemolytic or coagulation properties were found across all the transcriptomes. The predicted venom transcriptomes of myxozoans contained a notably high proportion of inhibitors of degradative enzymes (especially serine peptidases) (Fig. 2).

The transcriptome of *B. plumatellae*, obtained from individuals isolated from bryozoan hosts, encoded 124 potential toxins (Fig. 2A and Table S6), the largest group being peptidases (*n* = 58), including aspartic, serine and metallopeptidases. The metallopeptidases were astacins with and without ShK domains, endoplasmic reticulum aminopeptidases and carboxypeptidases. The next most diverse putative toxin protein family was the serine peptidase inhibitors (*n* = 23), followed by C-type lectin-like proteins (*n* = 9), CRiSP - cysteine rich, allergen like proteins (*n* = 7), and hydrolases including phospholipases (*n* = 5, with only phospholipase A-like proteins identified), lipases (*n* = 3), and a phosphatase like protein (*n* = 1).

The mixed myxosporean transcriptome (Fig. 2B and Table S7) also contained putative toxin peptidases (*n* = 33), uncharacterised toxin-like proteins (*n* = 7) and peptidase inhibitors (*n* = 17, both serine and cysteine inhibitors). We identified cytolytic and coagulation factors including natterins (*n* = 1) and stonustoxin-like toxins (*n* = 5), as well as C-type lectin like proteins (*n* = 4). Fibrogen related proteins (*n* = 4) and CRiSP—cysteine rich, allergen-like domain containing proteins (*n* = 3) were found. Toxin-like hydrolases were also identified including lipases (*n* = 5), phospholipases (*n* = 5), and a phosphatase (*n* = 1). One immune factor protein was identified with a homology to alpha-2-macroglobulin. Three transcripts had no recognised domains (see Table S7 for cnidarian toxin homology). It should be noted that as this dataset contained sequences from two different myxozoan species (*M. gasterostei* and *S. elegans*), these results represented the diversity of putative toxins shared between both species, rather than the complement of one myxozoan. Although there are some limitations to this interpretation, we surmise that at the level of class (Myxozoa vs other cnidarians) and within the Myxozoa (malacosporeans vs myxosporeans), this dataset provides useful insights into venom toxin diversity.

Transcriptomes of the other two cnidarians contained transcripts for more classical venom component enzymes, with serine proteases and metalloproteinases being the most diverse groups of transcripts. The toxin annotation pipeline identified 302 putative transcripts as potential toxins in the transcriptome of *C. cruxmelitensis* (Fig. 2C and Table S8). The most diverse putative toxin protein families were the peptidases (*n* = 121) comprising aspartic, serine and metallopeptidases. Other large groups included fibrogen-related toxins (*n* = 24, e.g., ficolin or ryncolin-like proteins), phosphatases (n=19), phospholipases including a phospholipase D/DNase like hydrolase (*n* = 13), and toxin proteins with C-type lectin domains (*n* = 10). The remaining putative toxins consisted of lipases (*n* = 8), peptidase inhibitors (*n* = 5), a “venom component C3” homolog (*n* = 1) and translationally controlled tumour protein (*n* = 1). Almost a quarter of the putative toxins (*n* = 60) had domains found commonly in animal toxins (e.g. SCP/CAP, chitin binding, ShK) but could not be characterised (classed as “Other” in Fig. 2C and see Table S8 for toxin homology). Additionally, 21 putative toxins did not encode any recognised toxin-like domains (7% of the 302).

Peptidases were also the most diverse of the putative toxin protein families annotated from the 214 potential toxin-encoding transcripts in the *P. hydriforme* transcriptome (Fig. 2D and Table S9). The largest group was the metallopeptidases which included astacins and neprilysin-like proteins. Some metallopeptidase transcripts encoded additional domains in a variety of orders and numbers (e.g. astacins with or without repeats of TSP, MAM and ShK domains). Other putative toxins, such as 5′ nucleotidases (*n* = 18), lipases (*n* = 8), both A and B phospholipases (*n* = 9), oxidases (*n* = 6), C-type lectin like proteins (*n* = 8) were also identified. As for *C. cruxmelitensis*, a large proportion of the putative toxin transcripts encoded recognised toxin domains, but were not characterised further (*n* = 23, ~13% of the flagged transcripts, see Table S8 for cnidarian toxin matches). Fifteen putative *P. hydriforme* toxins had no recognised domains. A higher proportion of putative toxin transcripts had predicted N-terminal signal peptides in *P. hydriforme* (42% (89/214)) than in *C. cruxmelitensis* (23% (70/302)) (Tables S8 and S9).

### Annotation of potential toxin-encoding peptides

Based on analyses of the myxozoan proteomes, only a small number of peptides were detected and identified as putative toxins compared to the number of toxins identified in the myxozoan transcriptomes (Fig. S6). Four of 124 putative toxin transcripts (3%) were translated in *B. plumatellae* and two of 96 in the mixed myxosporean material (2%) (see Tables S6 and S7). The four putative *B. plumatellae* toxins were assigned to three toxin protein families (CRiSP, C-type lectin and serine peptidase inhibitor) with potential neurotoxin, dysregulation of blood haemostasis and enzyme inhibitor biological functions (Table S6). The two putative toxins in the mixed myxosporean proteome were linked with similar functions, one being neurotoxin (verrucotoxin) and the other an inhibitor of blood haemostasis (Blarina toxin) (Table S7). These toxins may be shared between both *M. gasterostei* and *S. elegans* or unique to only one.

The numbers of potential toxin encoding transcripts translated and detected in the proteomes of *C. cruxmelitensis* and *P. hydriforme* were substantially higher than in the myxozoans (Fig. S6). In *C. cruxmelitensis* 64 of 302 transcripts (21%) were translated and in *P. hydriforme* 77 of 214 (35%) were translated (Table S10). The predicted toxins of *C. cruxmelitensis* venom had a broad range of predicted biological activities, the most prominent being cytotoxic (62 of 77 transcripts (81%)). Two putative neurotoxins, a phospholipase A2, and a serine protease inhibitor with homology to actitoxin of *Anemonia viridis* were detected. Eleven proteins lacked supporting data to ascribe a biological function or were involved in toxin maturation (e.g. glutaminyl-peptide cyclotransferase) (Tables S8 and S10). Peptides that could be annotated with predicted cytotoxic activity were part of either degradative enzymes or inhibitors of haemostasis and included metalloproteases (*n* = 25, including two proteins similar to snake venom metalloproteases, SVMPs), proteases (*n* = 9), lipases and phospholipases (*n* = 9), oxidases (*n* = 6), hydrolases (*n* = 5), and C-type lectins (*n* = 8). Most of the 77 translated putative toxins in the proteome of *P. hydriforme* (73%) (Tables S9 and S10) were annotated as degradative enzymes (peptidases (*n* = 33), lipases (*n* =10), glycoside hydrolase (*n* = 2) and 5′-nucleotidase (*n* = 2)) or inhibitors of blood haemostasis (C-type lectins (*n* = 8)). Peptides with predicted neurotoxin activity were considered minor components of the *P. hydriforme* venom proteome and included phospholipase A2 (Tables S9 and S10).

### Comparative venom profiles based on proteomics data

**Figure 3:**
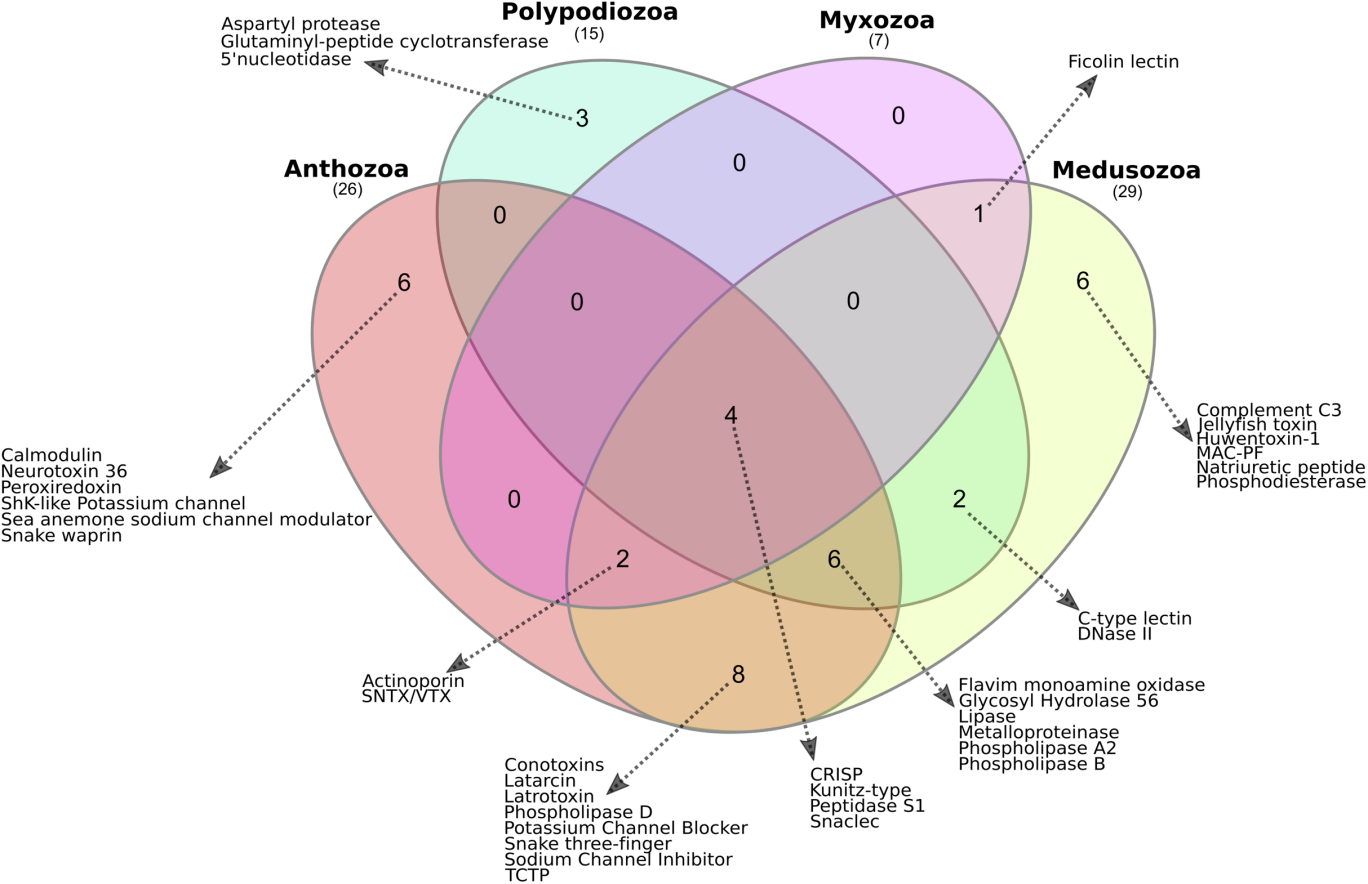
Comparison of the cnidarian putative venom proteomes. Venn diagram showing the number of putative toxin protein families shared among Polypodiozoa (*Polypodium hydriforme*), Myxozoa (*Buddenbrockia plumatellae*, *Myxobilatus gasterostei* and *Sphaerospora elegans*) and entirely free-living cnidarians (Anthozoa and Medusozoa). Overall, 10% (4/38) of the putative toxin protein families were shared by all groups. Only 39% (15/38) of the putative toxin protein families were unique to a single taxon, and Anthozoa and Medusozoa have 32% of unique putative toxin protein families (12/38), with no putative toxin protein families that were unique to Myxozoa. Aspartyl peptidase was unique to *Polypodium hydriforme* having never been described previously in the predicted venom of any cnidarian.

The presence or absence of translated proteins assigned to toxin families in Anthozoa, Medusozoa, *P. hydriforme* and myxozoans (Table S1) is summarised in Fig. 3. The Myxozoa had the smallest number of toxin protein families (*n* = 7), followed by *P. hydriforme* (*n* = 15), Anthozoa (*n* = 29) and Medusozoa (*n* = 29). Four toxin protein families were common to all groups (C-type lectins, serine peptidases, serine peptidase inhibitors, and CRiSP proteins). Six toxin protein families were unique to Medusozoa or Anthozoa, and an additional eight toxin protein families were shared only between these two lineages. No toxin protein families were unique to Myxozoa. Myxozoans shared six of their seven toxin protein families with both Anthozoa and Medusozoa, and one uniquely with Medusozoa. *Polypodium hydriforme* shared eight toxin protein families with Medusozoa and 10 jointly with Medusozoa and Anthozoa. Aspartyl peptidase was unique to *P. hydriforme*, having never been described previously in the predicted venom of any cnidarian (Fig. 3).

### Evolutionary analysis

**Figure 4:**
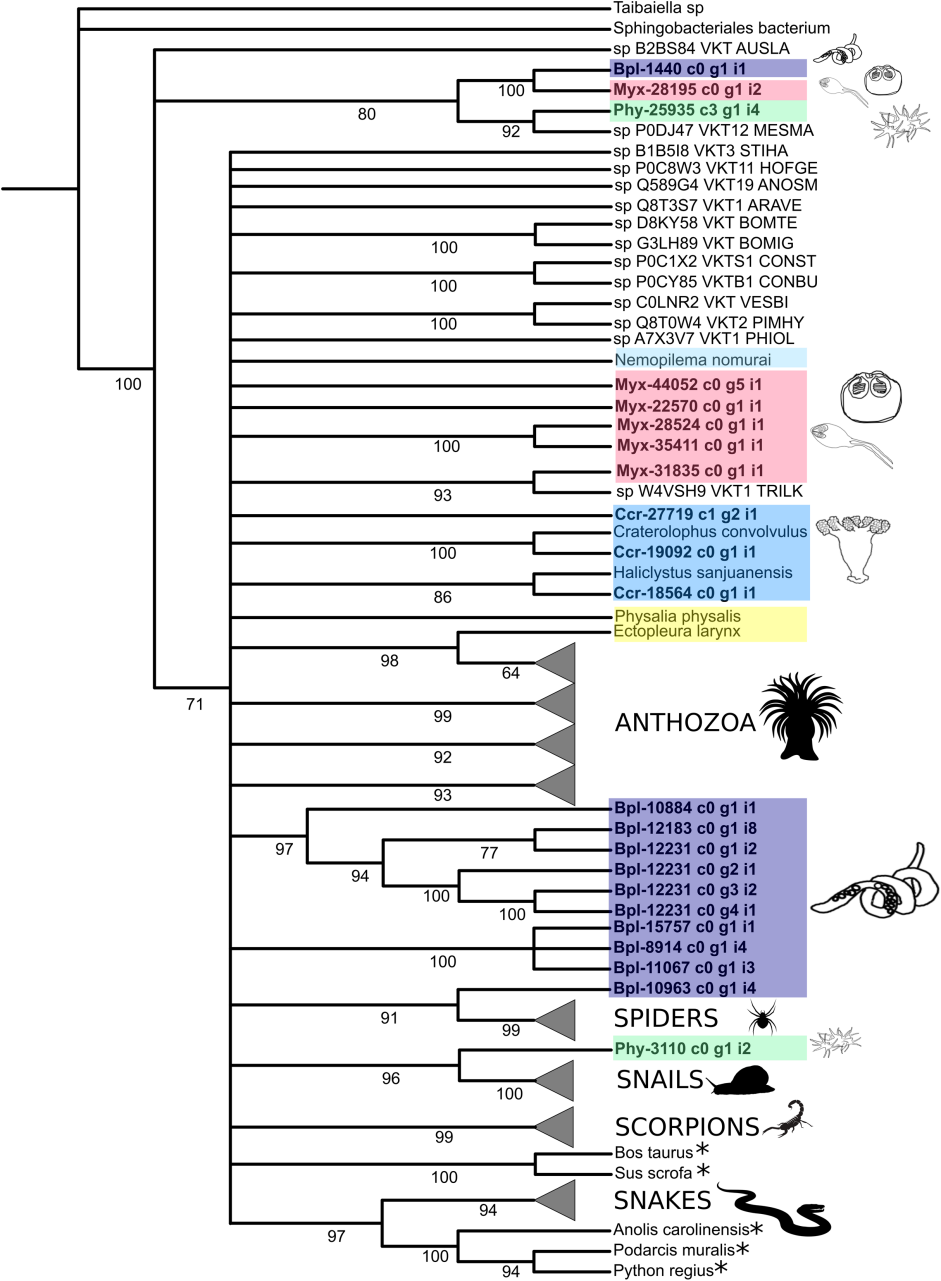
Phylogenetic tree of the Kunitz-type toxin gene family. The tree was constructed with maximum likelihood (ML) method using IQ-TREE version 2.1.1 software and with Bayesian inference using MrBayes version 3.2 based on amino acid sequences. Numbers below nodes show bootstrap support values from 1,000 replicates. Nodes that received ≤60% bootstrap support were collapsed. ML Phylogram was generated from amino-acid alignments of Kunitz-type homologs using WAG+I+G model. Abbreviations: An asterix (*) indicates non-toxin sequences, Bpl = *Buddenbrockia plumatellae* Ccr = *Calvadosia cruxmelitensis*; Myx = *Myxosporea*; Phy **= ***Polypodium hydriforme***. **A tree showing un-condensed groups is provided in Fig. S10.

**Figure 5:**
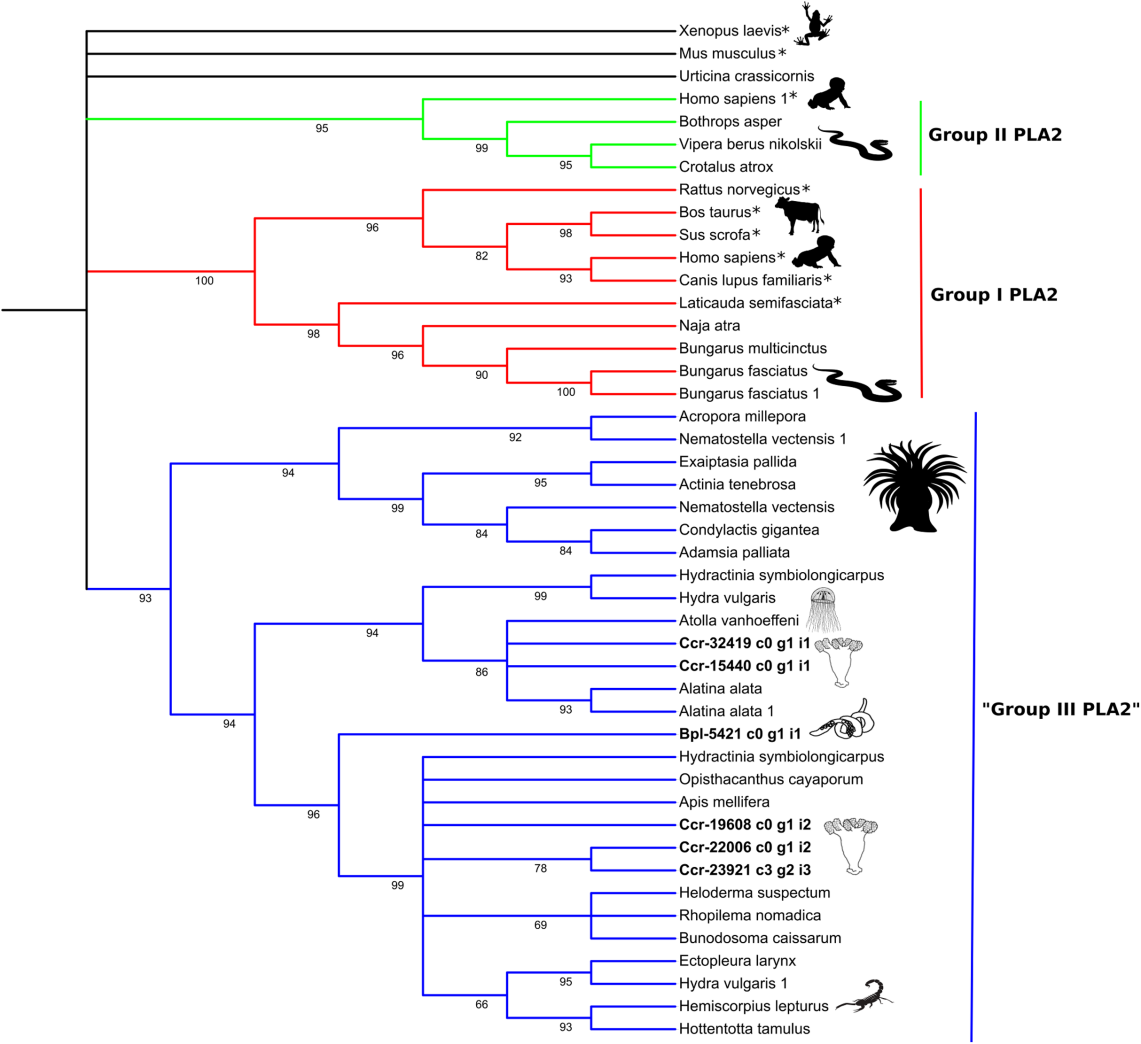
Phylogenetic tree of the Phospholipase A2 gene family. Numbers below nodes show bootstrap support values from 1,000 replicates. Nodes that received ≤60% bootstrap support were collapsed. ML Phylogram was generated from amino-acid alignments of Kunitz-type homologs using WAG+I+G model. Abbreviations: An asterix (*) indicates non-toxin sequences, Bpl = *Buddenbrockia plumatellae* Ccr = *Calvadosia cruxmelitensis*; Myx = Myxosporea; Phy = *Polypodium hydriforme*. ****

We used a molecular phylogenetic framework to clarify evolutionary trends and investigate the distribution of toxin-like genes found in Endocnidozoa and other animals. Phylogenetic hypotheses of the relationships between amino acid sequences of the putative venom Kunitz-type and phospholipase A2 were inferred in this study from a wide taxonomical range of venomous and non-venomous animals possessing these gene families (Tables S2 and S3). These two families were chosen because they have been frequently studied in the cnidarian literature and other venomous lineages, allowing reasonable comparative standards. Phylogenetic trees constructed from multiple alignments of amino acid sequences (Figs. S4 and S5) using maximum likelihood (venom Kunitz-type is shown in Fig. 4 and phospholipase A2 is shown in Fig. 5) and Bayesian inferences (Figs. S7 and S8) for both protein families have similar topology.

Phylogenetic analysis of the venom Kunitz-type protease inhibitors revealed an evolutionary pattern only partially congruent with the expected taxonomy, in which more inclusive groups (collapsed in Fig. 4) have low bootstrap support (≤60%). Cnidarian sequences are split in several less inclusive groups or as terminals along the tree (Fig. 4 and Table S11). Cnidarian groups were not resolved as monophyletic (the exception being Staurozoa whose outcome depended on resolution of the polytomy). Three endocnidozoan groups were supported (2 for *Buddenbrockia plumatellae* and 1 for mixed myxozoans). At least one sequence of each group of Endocnidozoa (i.e. *B. plumatellae*, mixed myxozoans, and *Polypodium hydriforme*) grouped with a venomous taxon (scorpions, spiders and conid snails), and, they never grouped together with other cnidarians. The substitution rates of the amino acid sequences of the venom Kunitz-type are significantly different when comparing Anthozoa and Endocnidozoa (*P* < 0.05; *P* = 0.023), with endocnidozoan lineages evolving slightly faster (cf. RRTree version 1.1; (Robinson-Rechavi & Huchon, 2000)) than those of free-living cnidarians.

The high bootstrap values in the phospholipase A2 tree (Fig. 5) support three distinct groups. Sequences in Group I (in red) and Group II (in green) derived from venomous snakes and non-venomous mammals. The third group (Group III; in blue) contains a basal sub-group composed of anthozoans which is sister to a sub-group comprising medusozoans (all four classes) and another sub-group with sequences of medusozoans, anthozoans, venomous non-cnidarian taxa (three scorpions, one honey bee, one lizard) and a sequence of *B. plumatellae*.

### Localisation of putative toxins in *B. plumatellae*

**Figure 6:**
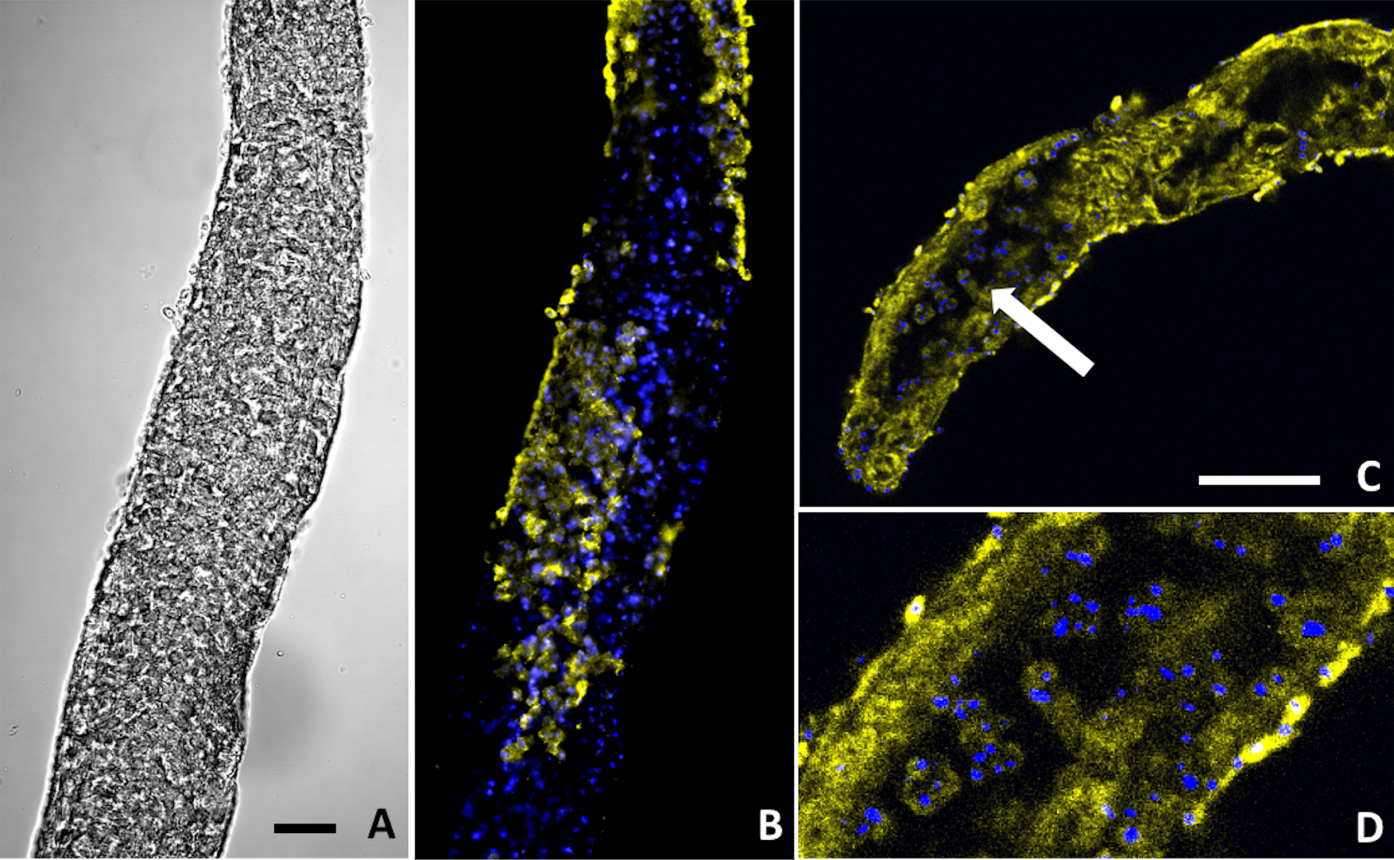
*Buddenbrockia plumatellae* myxoworms stained with polyclonal antibody raised towards lectin toxin. (A) Light microscopy image of presporogonic myxoworm. Scale 25 µm; (B) Confocal microscopy image of (A), with nuclei (blue) and lectin toxin localised (yellow) in cytoplasm of peripheral cells of presporogonic myxoworm; (C) Lectin (yellow) localised in cells to the inside of the muscle layer wall and cells in the early stages of sporogony (arrow) with DAPI stained nuclei (blue). Scale 50 µm; (D) Closer view of presporogonic cells in (C), with nuclei (blue) and lectin toxin (yellow) localised within myxoworm. ****

**Figure 7:**
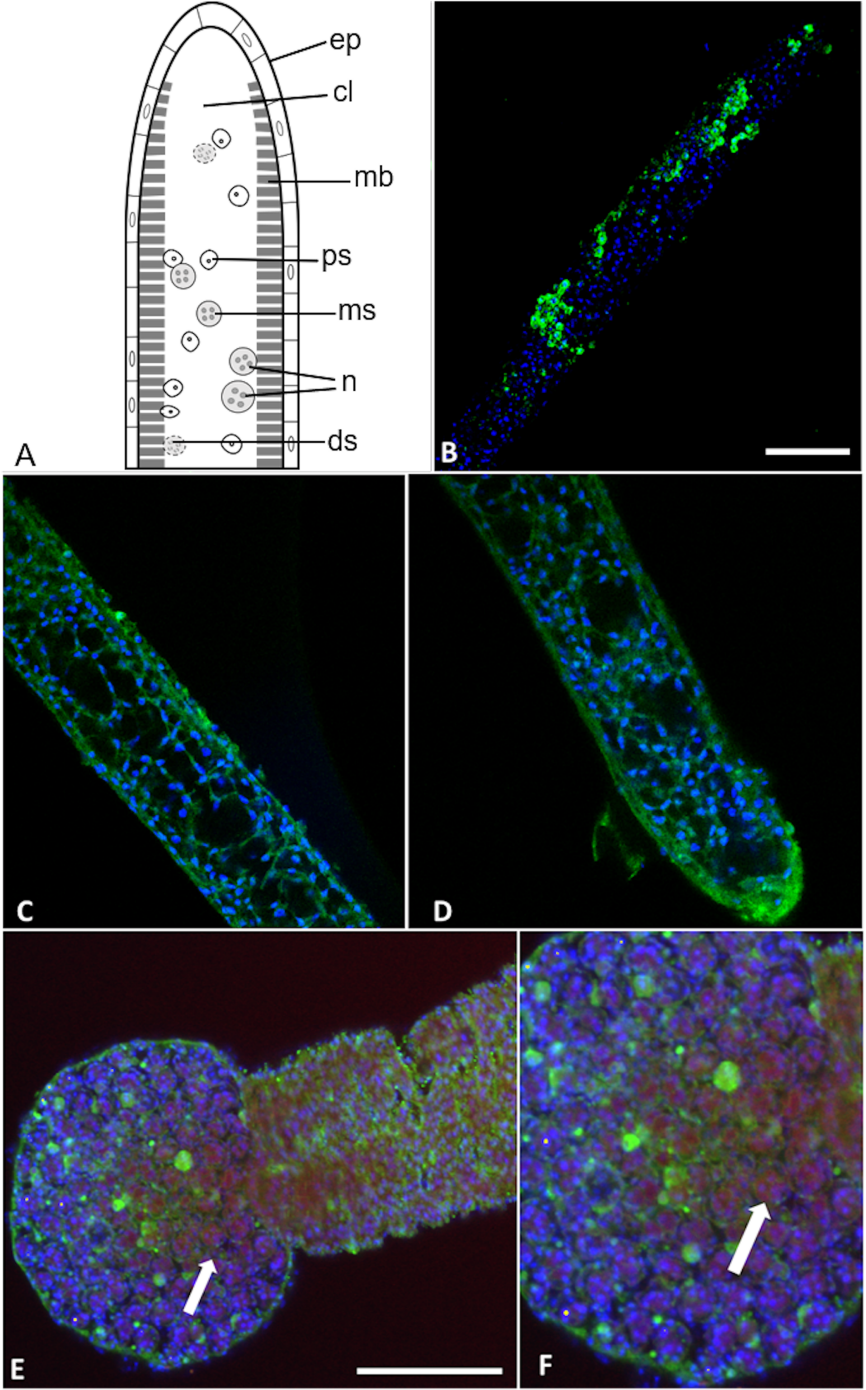
*Buddenbrockia plumatellae* myxoworms stained with polyclonal antibody raised towards serine peptidase inhibitor toxin. (A) Schematic of *Buddenbrockia plumatellae* vermiform stage indicating morphological organisation, ep, epithelium; cl, coelomic cavity; mb, muscle block; ds, developing spores; ms, malacospores; n, nematocyst; ps, presporogonic cells. (B) Serine peptidase inhibitor localised in peripheral cells (green) of presporogonic myxoworm, nuclei stained with DAPI (blue). Scale 60 µm; (C) and (D). Serine peptidase inhibitor (green) localised in cells to the inside of the muscle layer wall, cell nuclei (blue). (E) Spores in distal end of myxoworm with nuclei (blue) and actin (red). Four distinct spherical nematocysts are associated with mature spores. Note, serine peptidase inhibitor (green) localised near body wall surface and not with nematocysts of mature spores (arrow indicating cluster of malacospores). Scale 100 µm; (F). Closer view of (E) showing mature spores with actin (red) in cytoplasm and nuclei (blue), revealing slight staining of serine peptidase inhibitor (green) on surface of spores (arrow).

Two out of the three polyclonal antibodies raised against custom synthesised putative toxins identified from both transcriptomic and proteomic data for the myxoworm *B. plumatellae* isolated from bryozoan hosts were localised in early presporogonic stages (worms lacking spore development) (Figs. 6 and 7). Fluorescence corresponding to antibodies raised against a C-type lectin (Fig. 6) and to a serine peptidase inhibitor (Figs. 7C, 7D) was observed in the cytoplasm of cells internal to the muscle layer. Sporogony in *B. plumatellae* occurs internal to the muscle sheet, filling the inner cavity with malacospores (see Fig. 7A). Cells in this region are therefore assumed to be presporogonic stages. Both C-type lectin- and serine peptidase inhibitor expression was also observed in early sporogonic cells (Figs. 6C and 6D; Figs. 7C and 7D). Additionally, the serine peptidase inhibitor antibody was expressed in the cytoplasm of outer epithelial cells (Figs. 7B) and possibly on the external surface of malacospores (Figs. 7E and 7F). No signal was observed for the putative *B. plumatellae* CRiSP allergen in any of the developmental stages examined.

## Discussion

### Conservation, co-option and divergence of toxins

We present evidence that toxin genes have been conserved and repurposed in endoparasitic cnidarians, in support of H1. In particular, multiple bioinformatic analyses of transcriptomic and proteomic data infer that endocnidozoans utilise proteins with sequence homology to toxins found in venoms of free-living cnidarians and other animal taxa. In addition, localisation studies provide evidence for expression of selected toxins in cell populations involved in spore development and sub-epithelial cells but not within nematocyst-containing spores. The latter observation suggests a function other than venom delivery but further study is required to discount alternate explanations such as lack of detection (e.g. ultrastructural investigations to localise toxins in nematocysts or in sporoplasmosomes in spore cells), stage of maturity (e.g. toxins not yet transferred), or lack of dye penetration. Nevertheless, demonstration of toxin expression in itself illustrates a repurposing of venom activity because it is not associated with prey capture or defence as in free-living cnidarians. Notably, *P. hydriforme* achieves transmission using a specialised gonadophore that has been observed to attach to juvenile (pre-larvae) sterlet with nematocysts (Raikova, 1994) but whether toxins are involved is unknown.

Molecular phylogenetic analyses of the two expressed protein families show that the Endocnidozoa sequences may be closer to sequences from other cnidarians (like for PLA2, Fig. 5), although the molecular phylogenetic analyses of the Kunitz-type amino acid sequences have not elucidated the evolutionary relationships between both groups (Fig. 4). The resolution of the topology recovered with Kunitz-type is low and mainly supports deep nodes. Low bootstrap values can indicate conflicting or little signal in the data set. The latter may arise when sequences are very conserved, resulting in a data set with few informative sites and lack of information—a problem that can be exacerbated when analysing a limited and small functional domain such as Kunitz-type. Such sequence homogeneity can occur through processes such as natural selection, randomness or by intrinsic biases in the production of the molecular variability (Storz, 2016).

Interestingly, however, some endocnidozoan Kunitz-type sequences of *B. plumatellae*, mixed myxozoans and *P. hydriforme* group with sequences of venomous animals (other than cnidarians) providing evidence for divergence of venoms from ancestral free-living cnidarians (addressing H2) and potential convergent evolution of venoms in diverse animal groups. This contrasts with other myxozoan inhibitors (non-toxic) that group by taxonomy rather than physiological role (Eszterbauer et al., 2020). Fuzzy relationships with low bootstrap values and poor resolution were corroborated in a much broader analysis encompassing 356 sequences presenting the BPTI/Kunitz inhibitor mature domain obtained from UniProtKB/Swiss-Prot and including several families of Kunitz-type proteins (Figs. S9 and S11). The various phylogenetic analyses of the Kunitz-type toxin family suggested sequences were highly conserved which may account for lineage-specific gene duplications in many groups, including Endocnidozoa. Duplications may enhance the number of paralogs that then diversify in association with different biological functions, potentially further supporting H2.

### Endocnidozoan toxin diversity

The transcriptome for *B. plumatellae* stages developing in invertebrate hosts revealed an array of toxin-encoding transcripts similar to venom toxins of free-living cnidarians and a notably high percentage predicted to encode for serine protease inhibitors. However, relatively fewer toxin-encoding transcripts were demonstrated in myxozoans (124 in *B. plumatellae*; 96 shared between the mixed myxosporeans) than in *C. cruxmelitensis* (302, Fig. 2) and other free-living cnidarians. For example, some 170, 218 and 508 potential toxin transcripts were identified in transcriptomes of the cubozoan *Chironex fleckeri*, the scyphozoan *Stomolophus meleagris*, and the anthozoan *Stichodactyla haddoni*, respectively, on the basis of homology to known toxins in publicly available sequence databases (Brinkman et al., 2015; Madio, Undheim & King, 2017; Li et al., 2014). The detection of fewer toxin genes in myxozoans supports H3 and is concordant with reduced genomes sizes of myxozoans (Chang et al., 2015). In this case, loss may relate to relinquishing a predatory lifestyle. No myxozoan specific putative venom toxins were identified in the mixed myxosporean transcriptome or proteome datasets.

The predicted venom proteome of *P. hydriforme* contained a comparable number of putative toxins to those in the *C. cruxmelitensis* venom proteome (77 and 64, respectively). These results and the lack of detection of novel toxins in *B. plumatellae* suggest that toxin diversity is not linked with parasitism per se. In addition, they are consistent with evidence that life cycle complexity is not associated with an increased number of genes in cnidarians (Gold et al., 2019) (*P. hydriforme* has both free-living and parasitic stages while *C. cruxmelitensis* has a free-living, benthic stage only).

### Toxin demonstration, translation and function

Adopting a stringent approach to annotate putative toxins in transcriptomes and proteomes is crucial because toxin encoding gene families derive from housekeeping gene families. Expression of non-toxin homologues makes it notoriously difficult to identify toxins in homology-based annotations (Li et al., 2014; Hargreaves et al., 2014; Macrander, Broe & Daly, 2016). One approach to confirm toxin identity is to assess whether high expression levels are associated with venom glands (Smith & Undheim, 2018). This approach is precluded for Cnidaria which package toxins into intracellular organelles (nematocysts). Consequently, methods to distinguish transcripts encoding toxin from non-toxin homologs using sequence and domain homology searching methods are now standard in cnidarian venom research and were included here (see (Macrander et al., 2018) and references therein). In addition, we undertook molecular phylogenetic analyses of selected putative venom toxins analysing proteins in both venomous and non-venomous animals. The clustering of mainly Endocnidozoa sequences in both trees with homologs from other venomous animals provided further evidence that the sequences are toxins. These combined approaches greatly strengthen the evidence for the presence of genes that encode for venom toxins. Further substantiation of this scenario could include support for involvement in host interactions (e.g. localisation to excretory vesicles (sporoplasmosomes)) or demonstration that housekeeping homologs in venom-free tissues are distinct from putative toxins in molecular phylogenetic analyses.

There were surprisingly few matches between proteomic and transcriptomic sequences in the myxozoan data compared to their free-living counterparts (Fig. S6). Although 124 transcripts were predicted to encode for toxins in *B. plumatellae*, only four were translated (3%; Table S6). Similarly, only 2 of 96 mixed myxosporean transcripts (2%; Table S7) matched to the proteome data highlighting the importance of using proteomics to validate identification of putative toxins from transcriptome sequences, whilst also minimizing false positive identification of non-toxin homologs identified in transcriptomes (Madio, Undheim & King, 2017). Myxozoan proteomes revealed toxins in families with variable functions. These included procoagulant and anticoagulant toxins (C-type lectins in *B. plumatellae*; function demonstrated in snakes (Morita, 2004)) and neurotoxins such as verrucotoxin and blarina-like toxin from the mixed myxosporeans (shown to have vasodilation and paralysis properties in fish and shrews (Kita et al., 2004; Yazawa et al., 2007)). Additionally, cysteine-rich secretory proteins (CRiSPs) and serine peptidase inhibitors identified in *B. plumatellae* are associated with neurological and muscular dysfunction as ion channel blockers (Harvey, 2001; Yang et al., 2014) and with immune system modulation (Falcón et al., 2014; Ranasinghe et al., 2015).

Our localisation studies indicate translation of putative toxins in *B. plumatellae*. We found both the C-type lectin and serine peptidase inhibitor in presporogonic cells. Some epithelial cells forming the outer wall of a myxoworm also exhibited expression of serine peptidase inhibitor suggesting the possibility of toxin redeployment, for example in the breakdown of the muscle tissues (Canning et al., 2002) enabling spore release. There was also evidence for serine peptidase inhibitor on the outside of mature malacospores. These collective observations suggest that venom toxins are uniquely deployed in these endoparasites and further localisation studies in different life stages are now warranted to investigate possible repurposing of toxin function.

In the case of *P. hydriforme* there was a high proportion of matches between the proteome and transcriptome datasets (Table S9). Degradative enzymes (peptidases, lipases, hydrolases) made up the largest complement of putative toxins, representing 62% (48/77) of the toxin repertoire. These enzymes may assist the free-living stolon stage of *P. hydriforme* to digest remaining yolk reserves that fuel adoption of benthic life or they may be used for prey capture once yolk reserves are depleted and a mouth has formed (Raikova, 1994).

### Expanded understanding of cnidarian toxins and toxin families

Our three endocnidozoan venom proteomes expand the current picture of cnidarian venom composition while our collective evidence suggests that myxozoans produce complex and varied toxins that could have functioned as venoms in free-living ancestors. We observed one such toxin in cell populations of the outer body wall and the same toxin and another in cells involved in spore development. The former observation suggests repurposing of function from prey capture/defence via venom-delivering nematocysts to another function such as tissue degradation that facilitates release of spores from myxoworms (Canning et al., 2002). Whether toxins produced during spore development are eventually stored in nematocysts within spores requires further investigation.

Proteomic data suggest that 23 toxin protein families are shared amongst cnidarians (Fig. 3) and patterns of sharing between Endocnidozoa and other cnidarians imply an early diversity and long evolutionary history of a common venom complement (Jaimes-Becerra et al., 2019; Doonan et al., 2019). Notably, with the exception of ficolin lectin, all of the putative toxins detected in the myxozoan transcriptomes and proteomes have been reported variously in anthozoans (Fig. 3, Tables S1 and S6–S9). Our data thus provide evidence for the conservation of many core toxin families across free-living and endoparasitic cnidarians and raise questions about the early evolution of the venom trait.

## Conclusions

Our detection of relatively few venom-candidate proteins in myxozoans suggests that the general reduction in genome size in myxozoans includes reduced toxin diversity. At least some of these retained toxins have diverged. Future work is required to demonstrate how endocnidozoan toxins are deployed and if they are presented collectively as mixtures. Our localisation studies suggest that venom toxins may be uniquely used in endoparasites during development within hosts. For example, serine peptidase inhibitors may have been repurposed for self-destruction to liberate transmission stages. It would be of particular interest to determine sites and sources of toxin localisation, including from membrane-bound intracellular bodies (sporoplasmosomes) that likely fuse with cell membranes to secrete contents (Canning & Okamura, 2004), and by discharge from nematocysts. The latter would suggest deployment of ancestral function for host attachment using these nematocysts. The former may reflect repurposing of venom toxins for functions such as defence against the host environment or transmission (e.g. dissolution of parasite or host membranes). Confirmation of venom secretion from sporoplasmosomes in myxozoan cells would extend observations of venom secretion from cells in anthozoans (Moran et al., 2012) and a scyphozoan (Ames & Macrander, 2016) and would raise further questions about the nature of the ancestral venom-secreting cell type and diversification of secretory processes in Cnidaria (Mariscal, 1974; Beckmann & Özbek, 2012). We predict that venom repurposing has been critical in the adoption of endoparasitism and that cnidarian venom traits will eventually be viewed as a composite of ancestral and derived processes.

## Supplemental Information

10.7717/peerj.11208/supp-1 Supplemental Information 1 Detailed Materials and Methods.

10.7717/peerj.11208/supp-2 Supplemental Information 2 List of Supplementary Materials.

10.7717/peerj.11208/supp-3 Supplemental Information 3 Matching peptide fragments (highlighted in grey) from proteomics that match with translated transcriptomes of putative toxins.

10.7717/peerj.11208/supp-4 Supplemental Information 4 Kunitz venom type protein sequences used in the analysis in FASTA format.

10.7717/peerj.11208/supp-5 Supplemental Information 5 PLA2 protein sequences used in the analysis in FASTA format.

10.7717/peerj.11208/supp-6 Supplemental Information 6 Multiple alignment of Kunitz protein sequences.

10.7717/peerj.11208/supp-7 Supplemental Information 7 Multiple alignment of PLA2 protein sequences.

10.7717/peerj.11208/supp-8 Supplemental Information 8 Percentage of transcripts with MS/MS spectral matches vs percentage toxin transcripts with MS/MS spectral matches. Percentage of transcripts with MS/MS spectral matches vs percentage toxin transcripts with MS/MS spectral matches. Global MS/MS matches range from ~3.5-6%, however the subset of toxins with MS/MS greatly differs from 2-3.5% in parasitic vs free-living cnidarians.

10.7717/peerj.11208/supp-9 Supplemental Information 9 Phylogenetic tree of the Kunitz-type venom gene family. Phylogenetic tree of the Kunitz-type venom gene family. The tree was constructed with Bayesian inference using MrBayes version 3.2 based on amino acid sequences. Numbers in the nodes show posterior probability values.

10.7717/peerj.11208/supp-10 Supplemental Information 10 Phylogenetic tree of the Phospholipase A2 gene family. The tree was constructed with Bayesian inference using MrBayes version 3.2 based on amino acid sequences. Numbers in the nodes show posterior probability values.

10.7717/peerj.11208/supp-11 Supplemental Information 11 Phylogenetic tree of the Kunitz mature domain constructed with maximum likelihood (ML) method using IQ-TREE version 2.1.1 software. This tree was rooted with two plants sequences from I3A kunitz family.

10.7717/peerj.11208/supp-12 Supplemental Information 12 Phylogenetic Tree of the Kunitz-type venom gene family showing the un-condensed clades.

10.7717/peerj.11208/supp-13 Supplemental Information 13 Kunitz mature domain sequences of validated proteins that contain the Kunitz domain used in the analysis in FASTA format.

10.7717/peerj.11208/supp-14 Supplemental Information 14 Presence/absence matrix of toxin protein families in major cnidarian classes.

10.7717/peerj.11208/supp-15 Supplemental Information 15 Accession numbers for UNIPROT/genbank Kunitz sequences.

10.7717/peerj.11208/supp-16 Supplemental Information 16 Accession numbers for UNIPROT/genbank PLA2 sequences.

10.7717/peerj.11208/supp-17 Supplemental Information 17 ELISA measurements of specificity and sensitivity of the polyclonal antibodies raised against various putative *B. plumatellae* toxins.

10.7717/peerj.11208/supp-18 Supplemental Information 18 Transcriptome sequencing and assembly statistics.

10.7717/peerj.11208/supp-19 Supplemental Information 19 Putative venom toxins identified from the transcriptome and proteome of *B. plumatellae*.

10.7717/peerj.11208/supp-20 Supplemental Information 20 Putative venom toxins identified from the transcriptome and proteome of the mixed myxospreans.

10.7717/peerj.11208/supp-21 Supplemental Information 21 Putative venom toxins identified from the transcriptome and proteome of *C. cruxmelitensis*.

10.7717/peerj.11208/supp-22 Supplemental Information 22 Putative venom toxins identified from the transcriptome and proteome of *P. hydriforme*.

10.7717/peerj.11208/supp-23 Supplemental Information 23 Putative toxins identified from the proteomes of free-living cnidarians.

10.7717/peerj.11208/supp-24 Supplemental Information 24 Clade composition from the phylogenetic analysis of the venom Kunitz-type protease inhibitors given in Results figure 4.

## Additional Information and Declarations

### Field Study Permissions

The following information was supplied relating to field study approvals (i.e., approving body and any reference numbers): Collection sites did not require permissions to collect.

### Data Availability

The following information was supplied regarding data availability: Raw reads for the transcriptomes generated in this study are available at NCBI Sequence Read Archive (SRA), Bioproject number PRJNA576367. The mass spectrometry proteomics data are available at the ProteomeXchange Consortium via the PRIDE partner repository: PXD016306.
