# Supplementary material for: Recruitment of toxin-like proteins with ancestral venom function supports endoparasitic lifestyles of Myxozoa
Source: PeerJ. 2021 Apr 26;9:e11208. doi: 10.7717/peerj.11208 (PMC8083181; doi:10.7717/peerj.11208)
Supplement: Supplemental Information 1 [file peerj-09-11208-s001.docx]

**Supplementary Methods**

**Animal collections:** All permits necessary for collection and shipping of the below animals were obtained prior to the start of this study from the relevant government authorities.

*Malacosporea*: Colonies of the freshwater bryozoan, *Plumatella emarginata,* were collected from the River Aabach, Switzerland (47°23’35’’N, 8°23’63’’E) in August 2016. Bryozoans were examined using a stereomicroscope for infections of the vermiform malacosporean, *Buddenbrockia* *plumatellae,* by dissection. *B. plumatellae* worms recovered from dissected colonies and saved in RNAlater for transport on ice to the UK, where they were transferred to −80 °C for storage. Identification of *Buddenbrockia* and the bryozoan host was confirmed by mining ribosomal DNA sequences from the transcriptome obtained as part of this study.

*Myxosporea*: Adult individuals of three spine sticklebacks (*Gasterosteus aculeatus*) were caught using hand-held nets in Winterbourne Houghton (50°50’26’’N, -2.2°15’02’W), Dorset, UK in August 2016. Kidneys were aseptically dissected from the fish using a stereomicroscope and tissue smears were immediately inspected for myxozoan infections by light microscopy (x 200 magnification). Kidneys harboured mixed infections of *Sphaerospora elegans* and *Myxobilatus gasterostei* which could be identified by their distinct myxospore morphologies (henceforth referred to as ‘mixed myxosporeans’). Infected kidneys were then fixed in RNAlater (Thermo Fisher Scientific, Ashford, UK) and stored at −80 °C.

*Polypodium hydriforme*: *P. hydriforme* was collected from paddlefish (*Polyodon spathula*) caught in the river systems associated with Grand Lake o’ the Cherokees in northeast Oklahoma during the paddlefish fishing season in late March/early April 2017. We examined roe for *P. hydriforme* infections in fish transported for processing at the Paddlefish Research Center (Oklahoma Department of Wildlife Conservation). Free-living stolon stages of P. hydriforme that had emerged from infected eggs were collected. Specimens were fixed in RNAlater and held on ice for transport to the UK where they were then stored at −80 °C.

*Staurozoa*: Specimens of *Calvadosia cruxmelitensis* were collected during low tide in rock pools at Albert Pier Reef (50°12’07’’N, 5°53’04’’W) near Penzance, UK in December 2016. Species identity was confirmed by morphology [1]. Whole animals were preserved in RNAlater and maintained on ice for transfer to London, where they were then kept at -80 °C for long-term storage.

**RNA extraction:** The material used to isolate RNA was as follows: 50 stickleback kidneys of varying sizes with mixed myxosporean infections; approximately 500 *B. plumatellae* worms; 20 stolons of P. hydriforme, and the secondary tentacle clusters from each of 2 arms of 20 individual C. cruxmelitensis. RNA was isolated from all material using the same protocol. Briefly, samples were thawed on ice and RNAlater was removed and replaced with TRIzol Reagent. Samples were pooled and then homogenised 4 times for 5 mins using a Qiagen Tissue lyser II at a frequency of 30 Hz with 5 mm stainless steel beads (Qiagen). A 0.1 volume of 1-bromo-3-chloropropane was then added and the samples were centrifuged at 11, 000 rpm for 20 mins at 4 °C. RNA was extracted from the aqueous phase using isopropanol and MagMax magnetic beads (ThermoFisher Scientific, Hemel Hempstead, UK). The quality and quantity of the isolated RNA was determined using a NanoDrop ND-1000 and an Agilent RNA 6000 Nano Kit on an Agilent 2100 Bioanalyzer. RNA stocks were stored at −80 °C.

**Transcriptomics:** RNA with an RNA Integrity Number (RIN) above 7.0 was used for polyadenylation selected RNA library preparation with a NEBNext Poly(A) mRNA Magnetic Isolation Module (New England Biolabs Ltd., Hitchin, UK) using 100-200 ng total RNA as the starting material. Fragments with an average size of 300 bp were selected using the NEBNext Ultra Directional RNA Library Prep Kit. All samples were quantified by qPCR and Qubit. *B. plumatellae* was first sequenced on an Illumina MiSeq platform (150 cycle, 75bp paired-end reads) to assess sequence quality. Subsequently, RNA libraries of *B. plumatellae,* the mixed myxosporeans, P. hydriforme and *C. cruxmelitensis* were sequenced on an Illumina HiSeq 2500 sequencer (rapid mode) with paired-end 75 bp reads. Illumina reads were visualised using the fastqc v0.11.5 program to assess quality scores and to search for adapter and other over-represented sequences (https://www.bioinformatics.babraham.ac.uk/projects/fastqc/). Based on the fastqc report, the first 10 bases from sequences with fluctuating GC content (often caused by random hexamer priming [2], low quality leading and trailing bases with a quality score < 20, and Illumina adapters were removed from the sequence library with trimmomatic v0.36 [3]. Low abundance k-mers were corrected using Rcorrector with default settings [4].

**Removal of host contaminating sequences:** To filter the myxozoan and *P. hydriforme* raw transcriptomic reads from potentially contaminating fish sequences, the raw reads were mapped to the cDNA sequences of *Gasterosteus aculeatus* (<https://www.ensembl.org/Gasterosteus_aculeatus/Info/Index>) [5], *Rutilus rutilus* transcriptome GEBE00000000.1 and sequences from the *P. spathula* lateral-line transcriptome (*Polypodium* only) [6] (bio-project PRJNA357629) with bowtie2 [7] using the ‘very-sensitive’ option. Paired reads that did not align to the host datasets were used for transcriptome assembly. Additionally, the RNA from roughly 0.5 µg of 14-day-old colonies of *Plumatella vaihiriae* was extracted to generate a transcriptome for use as an indicator of potential host contamination in the *B. plumatellae* transcriptome. *P. emarginata* material was deemed to be unsuitable for host filtering due to the potential of covert infections which are typical of many malacosporeans (e.g. [8]). The *P. vaihiriae* material was originally sourced from Petchaburi, Thailand and was grown from statoblasts (dormant resting stages) in the laboratory (by Prof. Tim Wood at Wright State University, Dayton, Ohio). Microscopic inspection of the material did not indicate infections prior to fixation in RNAlater for our study, nor did later querying of the transcriptome identify any myxozoan infections. The alien index pipeline [9] was used to remove bryozoan sequences from the *Buddenbrockia* (invertebrate host) transcriptome. The ‘alien’ database was constructed using the assembled transcriptome of *P. vaihiriae*, the transcriptome of the bryozoan *Bugula neritina* [10], and bryozoan nucleotide sequences from the NCBI non-redundant database (taxid: 10205). A second database was also created, which contained cnidarian sequences amassed from the gene models of *Hydra vulgaris* [11], *Nematostella vectensis* [12] and *Acropora digitifera* [13]; the transcriptomes of *Aurelia aurita* [14] and *Anthopleura elegantissima* [15]; and the gene models and transcriptomes of the myxozoans *Enteromyxum leei*, *Kudoa iwatai* and *Myxobolus cerebralis* [16, 17]. The ‘alien’ and cnidarian sequences were combined to form a single database against which the nucleotide sequences of the *Buddenbrockia* transcriptome were aligned using blastn (max_target_seqs 1000, e-value 0.001). An alien index (AI) score was calculated for each alignment and any *Buddenbrockia* sequences that derived an AI score greater than 45 (20 orders of magnitude between the bryozoan and cnidarian e-values), or 0 < AI < 45 (known as ‘indeterminate’ matches) that had no hit to a cnidarian sequence were considered contaminants and were removed from the *Buddenbrockia* non-redundant transcriptome.

**Transcriptome assembly:** Each of the four transcriptomes was assembled with Trinity v2.6.6 using default settings [18]. For each assembly, ‘--max-memory’ was selected based on number of reads used (90-110GB). Assembled transcriptomes were next translated with OrfPredictor v3.0 [19] using default settings and all sequences greater than 30 amino acids in length were retained. Redundant proteins sequences were removed with CD-HIT v4.6 [20] using a cut off of 1.0 (i.e. 100 % identity). Further searches were also conducted to eliminate any fish sequences possibly contaminating the now assembled myxozoan and *P. hydriforme* transcriptomes. Specifically, nucleotide alignments of the non-redundant transcripts were constructed using the NCBI BLAST v2.2.31+ ‘blastn’ program with e-value 1e-10 [21]. All sequences with a sequence identity > 80% over 80% of the transcript length were considered to be contaminants and were removed. Gene completeness for each of the now non-redundant transcriptomes was assessed with BUSCO v3.0.2 using the metazoan odb 9 sequence database (<http://busco.ezlab.org/>) [22].

**Proteomics:** Soluble proteins were extracted from: 100 kidneys infected with the mixed myxosporeans, approximately 500 *B. plumatellae* worms, 20 individuals of P. hydriforme and the tentacle clusters from 20 individuals of *C. cruxmelitensis.* Tissues from each sample were thawed on ice and collected by centrifugation to remove RNAlater. The material was homogenised in microcentrifuge tubes using a microtube pestle in an equivalent weight of tissue to ice cold lysis buffer consisting of 100 mM Tris-HCl pH 7.5, 1 % (w/v) Triton X-100, 0.2 % (w/v) SDS, 300 mM NaCl, 0.2 % (w/v) sodium deoxycholate, 2 x protease inhibitors and 2 x phosphatase inhibitors (Halt Cocktail, Thermo Fisher Scientific Inc., Hemel Hempstead, UK). Samples were homogenised for 1 min, then incubated on ice for 4 mins. This was repeated over the course of 1hr. After 1 hr of protein extraction, the homogenate was centrifuged at 14000 x g for 20 mins at 4 ^O^C. The supernatant (soluble protein extract) was transferred to a fresh tube. An equivalent volume of lysis buffer was added to the collected pellet and the pellet was re-homogenised for 30 mins. After protein extraction, the homogenate was centrifuged at 14000 x g for 20 mins at 4 ^O^C. The supernatant was transferred to the protein extract collected previously. Extracted proteins were precipitated using a mixture of methanol, chloroform and water. The protein pellet was collected by centrifugation and dried to remove excess methanol. The pellet was solubilised in a buffer consisting of 100 mM Triethylammonium bicarbonate, 0.1 % (w/v) SDS and 10 mM Tris(2-carboxyethyl)phosphine hydrochloride. The pellet was then disrupted using a water bath sonicator and allowed to solubilise at room temperature. An aliquot of sample was taken to measure protein concentration by the Bradford assay. A total of 300 µg of protein was incubated for 1 hr at 55 ^O^C to reduce proteins. Iodoacetamide was then added to give a final concentration of 18 mM and samples were incubated for 30 mins protected from light to enable alkylation of proteins. In-solution digestion was performed by adding trypsin at a weight ratio of 1:40 trypsin to protein and the reaction mixture was then incubated overnight at 37 ^O^C. Trifluoroacetic acid was added to the sample to give a final concentration of 0.1 % (v/v). Prior to mass spectrometry analysis each sample was separated into 12 fractions either using off gel electrophoresis or high pH reversed phase chromatography. Hence, the extent of peptide separation was comparable between the samples with each dataset ultimately comprising of 12 LC-MS/MS raw files. Peptides fractions were then analysed by LC-MS/MS using an Ultimate 3000 nano-LC system in line with an Orbitrap Velos mass spectrometer (Thermo Scientific).  In brief, peptides in 1% (v/v) formic acid were injected onto an Acclaim PepMap C18 nano-trap column (Thermo Scientific). After washing with 0.5% (v/v) acetonitrile 0.1% (v/v) formic acid peptides were resolved on a 250 mm × 75 μm Acclaim PepMap C18 reverse phase analytical column (Thermo Scientific) over a 150 min organic gradient, using 7  gradient segments (1-6% solvent B over 1min., 6-15% B over 58 min., 15-32% B over 58 min., 32-40% B over 5 min., 40-90 % B over 1 min., held at 90 % B for 6 min and then reduced to 1 % B over 1min.) with a flow rate of 300 nL min^−1^. Solvent A was 0.1% formic acid and Solvent B was aqueous 80% acetonitrile in 0.1% formic acid. Peptides were subject to nano-electrospray ionization at 2.2 kV using a stainless-steel emitter with an internal diameter of 30 μm (Thermo Scientific) and a capillary temperature of 250°C. All spectra were acquired using an Orbitrap Fusion Tribrid mass spectrometer controlled by Xcalibur 2.0 software (Thermo Scientific) and operated in data-dependent acquisition mode.  FTMS1 spectra were collected at a resolution of 120,000 over a scan range (m/z) of 350-1550, with an automatic gain control (AGC) target of 400,000 and a max injection time of 100 ms. The Data Dependent mode was set to Cycle Time with 3 sec between master scans.  Precursors were filtered according to charge state (to include charge states 2-7), with monoisotopic precursor selection and using an intensity range of 5E3 to 1E20. Previously interrogated precursors were excluded using a dynamic window (40s +/-10ppm). The MS2 precursors were isolated with a quadrupole mass filter set to a width of 1.6 m/z. ITMS2 spectra were collected with an AGC target of 5000, maximum injection time of 50 ms and HCD collision energy of 35%.

**Identification of putative toxin encoding transcripts and potential toxins from proteomic data:** Putative toxin proteins were identified in-line with previously described toxin annotation pipelines, with minor modifications [23, 24, 25]. Briefly, translated non-redundant proteins from each transcriptome were matched with BLASTp (e-value 1e-05, bit score > 50) against the Uniprot Swissport database and a customised toxin dataset which consisted of the ToxProt database [26] (downloaded 12/07/2017) supplemented with putative cnidarian toxin sequences reported in the literature but not deposited in either the Uniprot Swissprot or ToxProt databases [23, 24, 25, 27]. A custom Perl script was used to identify and compare matches from both BLAST analyses. A transcript was deemed to be a potential toxin sequence if the custom toxin database BLAST hit had a bit score equal to, or higher than, the corresponding Uniprot hit. This acted as an initial screen to for putative toxin identification. For each set of putative toxins, conserved domains were predicted using InterProScan5 [28], and these predictions were used to assign sequences to the most likely toxin protein family and remove potential false positives. The assignments were then checked by a manual search of the scientific literature to validate the putative toxin function. Unique MS/MS spectral events were visualised using PEAKS Studio software v8.5 [29]. Peptides for each animal were back translated and paired against the corresponding non-redundant transcriptome using the PEAKS proprietary matching algorithm. A protein identification score -10lgP of >20 was used as the default cut off setting. Peptide-spectral matches to putative toxins previously annotated from the transcriptome were extracted and manually validated for unbroken series of overlapping b-type and y-type sequence specific fragments ions, where losses consistent with the sequence were acceptable quality. Alignments of peptide-spectral matches to putative toxins previously annotated from the transcriptome are given in figure S1.

**Comparative toxin profiles:** To compare toxin diversities a Venn diagram was constructed using InteractiVenn [30] from a matrix of presence and absence of putative toxin protein families identified in this study and in 11 previously published venom proteomes (*Anemonia viridis*, *Tubastraea coccinea*, *A. digitifera*, *Ectopleura crocea*, *Olindias sambaquiensis*, *Tamoya haplonema*, *Chiropsalmus quadrumanus*, *Haliclystus antarcticus*, *Chrysaora lactea*, *Aurelia aurita* and *Hydra magnipapillata* to reflect total similarity [31]. The previously published venom proteomes were selected to ensure identical bioinformatics approaches for annotating unique MS/MS events were employed across studies. The toxins from the combined data set were assigned to venom toxin protein families using established KEGG ontology. Data were coded in a matrix as presence (1) or absence (0) of each toxin protein family in each species and is shown in electronic supplementary material, table S1.

**Evolutionary analysis:** Phospholipase A2 (PLA2) and venom Kunitz-type protein sequences from 5 taxa (sea anemones, snails, insects, scorpions, spiders, and snakes) with extensively studied venom as well as protein sequences of some non-venomous taxa were obtained from the Uniprot database and from published cnidarian transcriptomes (electronic supplementary material, tables S2 and S3). For Kunitz-type family analyses, all protein sequences reviewed and categorized as venom Kunitz-type were recovered from the Uniprot database (255 total). These sequences are provided in FASTA format in electronic supplementary material, figures S2 and S3, together with the sequences generated in this study. Only the Endocnidozoa and *C. cruxmelitensis* protein sequences derived from unique genes were included in the data set. Sequences derived from mRNA splice variants were excluded. Likewise, only the Endocnidozoa and *C. cruxmelitensis* protein sequences in which a signal peptide was predicted were used. Signal peptide prediction was performed using SignalP-5.0 [32]. Evolutionary divergence of endocnidozoan toxins in relation to free-living cnidarians was inferred by constructing trees for the two toxin families and using the program RRTree version 1.1 [33] that compares substitution rates between protein sequences grouped or not in phylogenetically defined lineages. The comparison of the substitution rates was conducted between the groups free-living cnidarians and endocnidozoans only for Kunitz-type family.

For the phylogenetic analyses, protein sequences were aligned using the algorithm L-INS-I in MAFFT [34]. A best-fit model of amino acid replacements was determined according to Akaike’s information criterion using Prottest 3.0 [35]. For the PLA2 family the best-fit model was WAG+I+G. *Xenopus laevis* PLA2 sequence was used as outgroup. For the venom Kunitz-type toxin family the best-fit model was WAG+I+G. Two prokaryotes BPTI/Kunitz inhibitor domain-containing protein sequences were used as outgroups. Phylogenetic relationships were deduced using a maximum likelihood (ML) approach. Optimal ML phylogenetic trees were constructed using IQ-TREE version 2.1.1 [36] and node support was obtained with 1000 ultrafast bootstrap [37]. Searches with multiple 500 initial parsimony trees were performed. Bootstrap support values were assigned on the branches of the best maximum likelihood tree. Similarly, phylogenetic trees were reconstructed by Bayesian analysis using MrBayes v 3.2.2 [38]. The analysis was run twice, each run used four Markov chains, sampling every 1,000th generations for 20,000,000 generations, and used a default burn-in value of 25%, with lset rates = invgamma and prset aamodel = fixed (Wag).

The command line used in IQ-TREE to construct the PLA2 family tree with supports was:

./iqtree2 -s PLA2.phy -m WAG+I+G -allnni -ninit 500 -nbest 25 -ntop 100 --bb 1000 -pre PLA2

The command line used in IQ-TREE to construct the venom Kunitz-type protein family tree with supports was:

./iqtree2 -s Kunitz.phy -m WAG+I+G -allnni -ninit 500 -nbest 25 -ntop 100 --bb 1000 -pre Kunitz

**Localisation of putative toxins in *B. plumatellae*:** Custom peptides were synthesised for 3 putative toxins identified from the *B. plumatellae* transcriptome with additional proteomics evidence that supported translation. Rabbit polyclonal antibodies were raised against these three proteins (Vertebrate Antibodies Limited., Aberdeen, UK). Specificity and sensitivity of the polyclonal antibodies against the proteins was measured by ELISA and are shown in electronic supplementary material, table S4. Vermiform worm stages of *B. plumatellae* were isolated from their bryozoan hosts and fixed in 4 % (v/v) paraformaldehyde in 0.1 M PBS for 24 hrs and then maintained at -20 ºC in methanol. Whole mount specimens were permeabilized using 0.1 % (v/v) Triton in 0.1 M PBS (1 hr room temperature), blocked using 0.1 % (v/v) BSA (3 hrs room temperature), washed and then incubated with one of the three primary polyclonal antibodies (1:500) overnight at 4 ºC. Samples were then washed for at least 6 hrs and then incubated with a secondary Alexafluor-conjugated anti-rabbit antibody (1:500) overnight at 4 º C or 3 hrs at room temperature. Tissues were then washed, and the nuclei stained with DAPI Fluoroshield (Invitrogen). Stained tissue preparations were visualised using a Nikon Eclipse upright microscope with A1-Si confocal microscope. Images were analysed and adjusted using NIS-Elements software.

**Acknowledgments**

The authors thank Drs Han Lu and Sucharitha Balu from the National Institute for Health Research (NIHR) Biomedical Research Genomics Centre at King’s College London for equipment access. Additional sequencing was performed at the Biomedical Research Genomics Centre at Imperial London. The authors thank Dr Ayham Alnabulsi at Vertebrate Antibodies Limited in Aberdeen, for raising and purifying the antibodies used in the localisation studies. The authors acknowledge use of the research computing facility at King’s College London, *Rosalind* (<https://rosalind.kcl.ac.uk>), which is delivered in partnership with the National Institute for Health Research (NIHR) Biomedical Research Centres at South London & Maudsley and Guy’s & St. Thomas’ NHS Foundation Trusts, and part-funded by capital equipment grants from the Maudsley Charity (award 980) and Guy’s & St. Thomas’ Charity (TR130505). The views expressed in the manuscript are those of the author(s) and not necessarily those of the NHS, the NIHR, King’s College London, or the Department of Health and Social Care.

**References**

1. L. S. Miranda, Y. M. Hirano, C. E. Mills, A. Falconer, D. Fenwick, *et al*., Systematics of stalked jellyfishes (Cnidaria: Staurozoa). *PeerJ*. **4**, e1951, (2016).
2. K. D. Hansen, S. E. Brenner, S. Dudoit, Biases in Illumina transcriptome sequencing caused by random hexamer priming. *Nucleic Acids Res*. **38**, e131, (2010).
3. A. M. Bolger, M. Lohse, B. Usadel, Trimmomatic: a flexible trimmer for Illumina sequence data. *Bioinformatics*. **30**, 2114‐2120, (2014).
4. L. Song, L. Florea, Rcorrector: efficient and accurate error correction for Illumina RNA-seq reads. *GigaSci* **4,**48, (2015).
5. F. C. Jones, Y. F. Chan, J. Schmutz, J. Grimwood, S. D. Brady, *et al*., A genome-wide SNP genotyping array reveals patterns of global and repeated species-pair divergence in sticklebacks. *Curr. Biol.* **22**, 83-90, (2012).
6. M. S. Modrell, M. Lyne, A. R. Carr, H. .H Zakon, D. Buckley D, *et al*., Insights into electrosensory organ development, physiology and evolution from a lateral line-enriched transcriptome. *Elife*. **6**, e24197, (2017).
7. B. Langmead, S. L. Salzberg, Fast gapped-read alignment with Bowtie 2. *Nat. Methods*. **9**, 357-359, (2012).
8. I. Fontes, H. Hartikainen, C. Williams, B. Okamura, Persistence, impacts and environmental drivers of covert infections in invertebrate hosts. *Parasit. Vectors*. **10**, 542, (2017).
9. J. F. Ryan, Alien_Index: Identify Potential Non-Animal Transcripts in Animal Transcriptomes. Available online: <https://github.com/josephryan/alien_index,> (2014).
10. Wong YH, Ryu T, Seridi L, Ghosheh Y, Bougouffa S, *et al*., Transcriptome analysis elucidates key developmental components of bryozoan lophophore development. *Sci Rep.* **4**, 6534, (2014).
11. I. Chapman, E. Kirkness, O. Simakov, S. E. Hampson, T. Mitros *et al.* The dynamic genome of *Hydra*. *Nature.* **464,**592–596 (2010).
12. Putnam NH, Srivastava M, Hellsten U, Dirks B, Chapman J, *et al*., Sea anemone genome reveals ancestral eumetazoan gene repertoire and genomic organization. *Science*. **317**, 86-94, (2007).
13. C. Shinzato, E. Shoguchi, T. Kawashima, T. M. Hamada, K. Hisata *et al.* Using the *Acropora digitifera* genome to understand coral responses to environmental change. *Nature.* **476,**320–323, (2011).
14. V. Brekhman, A. Malik, B. Haas, N. Sher, T. Lotan, Transcriptome profiling of the dynamic life cycle of the scypohozoan jellyfish *Aurelia aurita*. *BMC Genomics.* **16,**74 (2015).
15. S. A. Kitchen, C. M. Crowder, A. Z. Poole, V. M. Weis, E. Meyer, *de novo* Assembly and Characterization of Four Anthozoan (Phylum Cnidaria) Transcriptomes. *G3 (Bethesda)*. **5**, 2441-2452, (2015).
16. E. S. Chang, M. Neuhof, N. D. Rubinstein, A. Diamant, H. Philippe, D. Huchon, P. Cartwright. Genomic insights into the evolutionary origin of Myxozoa within Cnidaria. *Proc. Natl. Acad. Sci. U. S. A.* **112**, 14912-14917, (2015).
17. Shpirer E, Chang ES, Diamant A, Rubinstein N, Cartwright P, Huchon D. Diversity and evolution of myxozoan minicollagens and nematogalectins. *BMC Evol. Biol.* **14**, 205–217, (2014).
18. B. J. Haas, A. Papanicolaou, M. Yassour, M. Grabherr, P. D. Blood, *et al*., *de novo* transcript sequence reconstruction from RNA-seq using the Trinity platform for reference generation and analysis. *Nat. Protoc.* **8**, 1494-1512, (2013).
19. X. J. Min, G. Butler, R. Storms, A. Tsang, OrfPredictor: predicting protein-coding regions in EST-derived sequences. *Nucleic Acids Res.* **33** (Web Server issue), W677-80, (2005).
20. W. Li, A. Godzik, Cd-hit: a fast program for clustering and comparing large sets of protein or nucleotide sequences. *Bioinformatics*. **22**, 1658-1659, (2006).
21. C. Camacho, G. Coulouris, V. Avagyan, N. Ma, J. Papadopoulos, *et al*., BLAST+: architecture and applications. *BMC Bioinformatics*. **10**, 421, (2009).
22. F. A. Simão, R. M. Waterhouse, P. Ioannidis, E. V. Kriventseva, E. M. Zdobnov, BUSCO: assessing genome assembly and annotation completeness with single-copy orthologs. *Bioinformatics*. **31**, 3210-3212, (2015).
23. D. L. Brinkman, X. Jia, J. Potriquet, D. Kumar, D. Dash, *et al*., Transcriptome and venom proteome of the box jellyfish *Chironex fleckeri*. *BMC Genomics*. **16**, 407 (2015).
24. R. Gacesa, R. Chung, S. R. Dunn, A. J. Weston, A. Jaimes-Becerra, *et al*., Gene duplications are extensive and contribute significantly to the toxic proteome of nematocysts isolated from *Acropora digitifera* (Cnidaria: Anthozoa: Scleractinia). *BMC Genomics*. **16**, 774, (2015).
25. D. Ponce, D. L. Brinkman, J. Potriquet, J. Mulvenna, Tentacle Transcriptome and Venom Proteome of the Pacific Sea Nettle, *Chrysaora fuscescens* (Cnidaria: Scyphozoa). *Toxins (Basel)*. **8**, 102, (2016).
26. F. Jungo, L. Bougueleret, I. Xenarios, S. Poux, The UniProtKB/Swiss-Prot Tox-Prot program: A central hub of integrated venom protein data. *Toxicon*. **60**, 551-557, (2012).
27. J. Macrander, M. Broe, M. Daly, Multi-copy venom genes hidden in *de novo* transcriptome assemblies, a cautionary tale with the snakelocks sea anemone *Anemonia sulcata* (Pennant, 1977). *Toxicon.* **108**, 184-188, (2015).
28. P. Jones, D. Binns, H. Y. Chang, M. Fraser, W. Li, *et al*., InterProScan 5: genome-scale protein function classification. *Bioinformatics*. **30**, 1236-1240, (2014).
29. J. Zhang, L. Xin, B. Shan, W. Chen, M. Xie, *et al*., PEAKS DB: *de novo* sequencing assisted database search for sensitive and accurate peptide identification. *Mol. Cell. Proteomics*. **11**, M111.010587, (2012).
30. H. Heberle, G. V. Meirelles, F. R. da Silva, G. P. Telles, R. Minghim, InteractiVenn: a web-based tool for the analysis of sets through Venn diagrams. *BMC Bioinformatics*. **16**, 169, (2015).
31. A. Jaimes-Becerra, R. Gacesa, L. B. Doonan, A. Hartigan, A. C. Marques, *et al*., Beyond primary sequence—proteomic data reveal complex toxins in cnidarian venoms. *Integr. Comp. Biol.* **59**, 777-785, (2019).

J. J. Almagro Armenteros, K. D. Tsirigos, C. K. Sønderby, T. Nordahl Petersen, O. Winther, S. Brunak, G. von Heijne, H. Nielsen, SignalP 5.0 improves signal peptide predictions using deep neural networks. *Nat. Biotech.* **37**, 420–423, (2019).

M. Robinson-Rechavi, D. Huchon, RRTree: Relative-Rate Tests between groups of sequences on a phylogenetic tree. *Bioinformatics* **16**, 296-297, (2000).

1. D. Darriba, G. L. Taboada, R. Doallo, D. Posada, ProtTest 3: fast selection of best-fit models of protein evolution. *Bioinformatics*. **27**, 1164-1165, (2011).
2. A. Stamatakis, RAxML version 8: a tool for phylogenetic analysis and post-analysis of large phylogenies. *Bioinformatics*. **30**, 1312-1313, (2014).
3. L. -T. Nguyen, H. A. Schmidt, A. von Haeseler, B. Q. Minh, IQ-TREE: A fast and effective stochastic algorithm for estimating maximum likelihood phylogenies. *Mol. Biol. Evol.* **32**, 268-274, (2015).
4. D. T. Hoang, O. Chernomor, A. von Haeseler, B. Q. Minh, L. S. Vinh, UFBoot2: Improving the ultrafast bootstrap approximation. *Mol. Biol. Evol.* **35**, 518–522, (2018).

J. P. Huelsenbeck, F. Ronquist, MRBAYES: Bayesian inference of phylogeny. *Bioinformatics*. **17**, 754-755 (2001).
