# Supplementary material for: Recruitment of toxin-like proteins with ancestral venom function supports endoparasitic lifestyles of Myxozoa
Source: PeerJ. 2021 Apr 26;9:e11208. doi: 10.7717/peerj.11208 (PMC8083181; doi:10.7717/peerj.11208)
Supplement: Supplemental Information 2 [file peerj-09-11208-s002.docx]

**List of electronic supplementary materials**

figure S1: Matching peptide fragments (highlighted in grey) from proteomics that match with translated transcriptomes of putative toxins.

figure S2: Kunitz venom type protein sequences used in the analysis in FASTA format

figure S3: PLA2 protein sequences used in the analysis in FASTA format

figure S4: Multiple alignment of Kunitz protein sequences

figure S5: Multiple alignment of PLA2 protein sequences

figure S6: Percentage of transcripts with MS/MS spectral matches vs percentage toxin transcripts with MS/MS spectral matches. Global MS/MS matches range from ~3.5-6%, however the subset of toxins with MS/MS greatly differs from 2-3.5% in parasitic vs free-living cnidarians.

figure S7: Phylogenetic tree of the Kunitz-type venom gene family. The tree was constructed with Bayesian inference using MrBayes version 3.2 based on amino acid sequences. Numbers in the nodes show posterior probability values.

figure S8: Phylogenetic tree of the Phospholipase A2 gene family. The tree was constructed with Bayesian inference using MrBayes version 3.2 based on amino acid sequences. Numbers in the nodes show posterior probability values.

figure S9: Phylogenetic tree of the Kunitz mature domain constructed with maximum likelihood (ML) method using IQ-TREE version 2.1.1 software. This tree was rooted with two plants sequences from I3A kunitz family.

figure S10: Phylogenetic Tree of the Kunitz-type venom gene family showing the un-condensed clades.

figure S11: Kunitz mature domain sequences of validated proteins that contain the Kunitz domain used in the analysis in FASTA format

table S1: Presence/absence matrix of toxin protein families in major cnidarian classes

table S2: Accession numbers for UNIPROT/genbank Kunitz sequences

table S3: Accession numbers for UNIPROT/genbank PLA2 sequences

table S4: ELISA measurements of specificity and sensitivity of the polyclonal antibodies raised against various putative *B. plumatellae* toxins.

table S5: Transcriptome sequencing and assembly statistics

table S6: Putative venom toxins identified from the transcriptome and proteome of *B. plumatellae*

table S7: Putative venom toxins identified from the transcriptome and proteome of the mixed myxospreans

table S8: Putative venom toxins identified from the transcriptome and proteome of *C. cruxmelitensis*

table S9: Putative venom toxins identified from the transcriptome and proteome of *P. hydriforme*

table S10: Putative toxins identified from the proteomes of free-living cnidarians

table 11: Clade composition from the phylogenetic analysis of the venom Kunitz-type protease inhibitors given in Results figure 4.
