# Supplementary material for: Recruitment of toxin-like proteins with ancestral venom function supports endoparasitic lifestyles of Myxozoa
Source: PeerJ. 2021 Apr 26;9:e11208. doi: 10.7717/peerj.11208 (PMC8083181; doi:10.7717/peerj.11208)
Supplement: Supplemental Information 3 [file peerj-09-11208-s003.docx]

Bpl-12231_c0_g3_i2


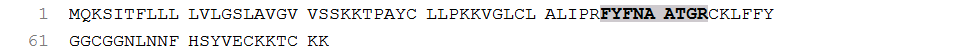


Bpl-10897_c0_g1_i1


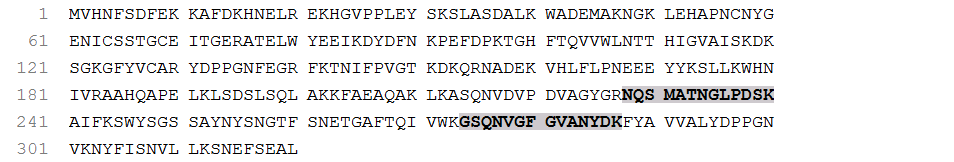


Bpl-10897_c0_g2_i2


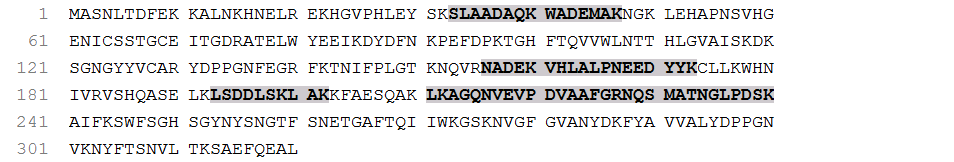


Bpl-13734_c0_g2_i5


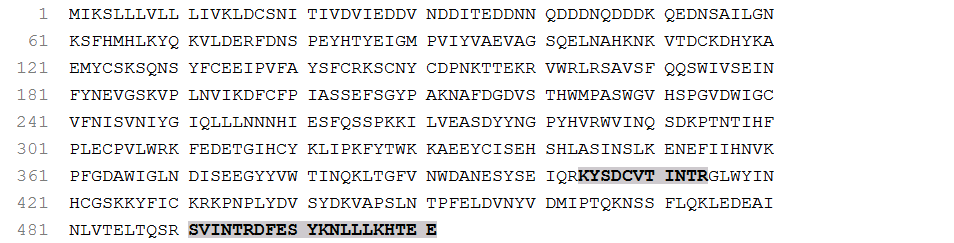


Ccr-20068_c0_g1_i1


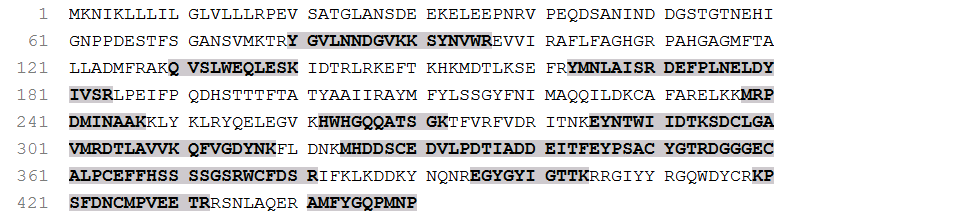


Ccr-21624_c0_g1_i1


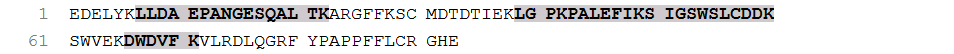


Ccr-21624_c0_g2_i1


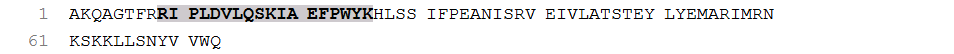


Ccr-22147_c7_g1_i1


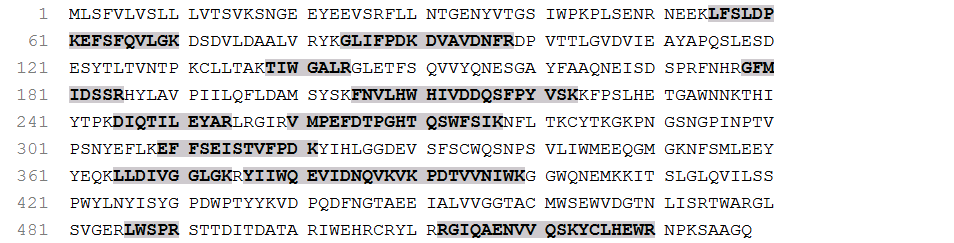


Ccr-22960_c1_g1_i4


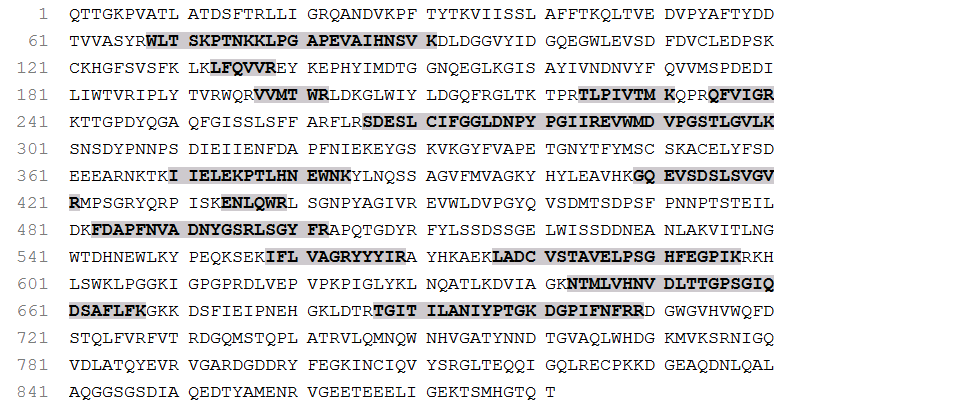


Ccr-23322_c6_g1_i2


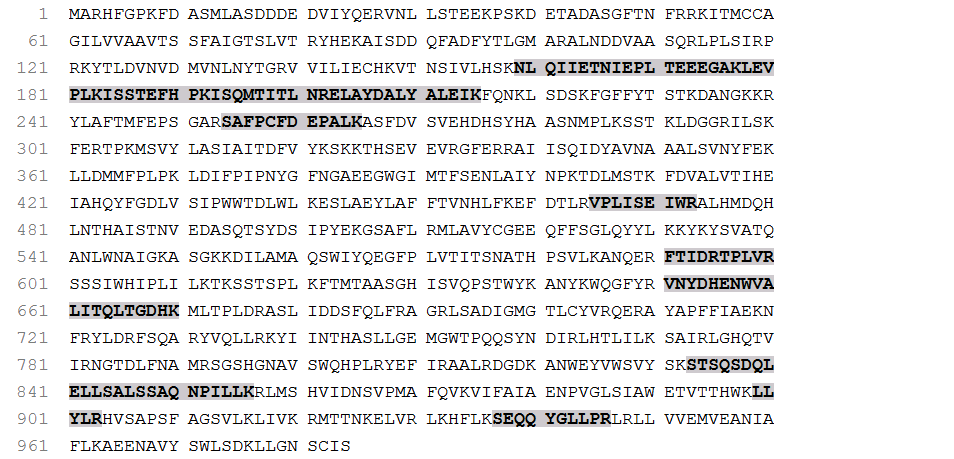


Ccr-23655_c0_g2_i1


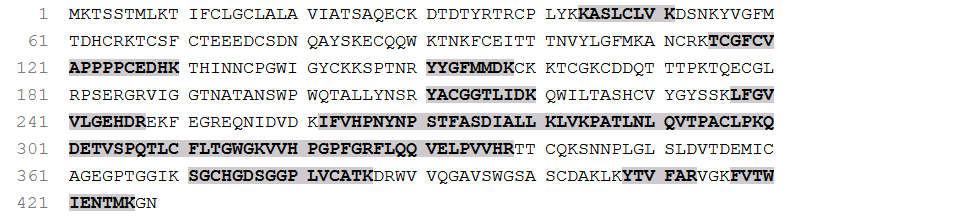


Ccr-24550_c2_g2_i2


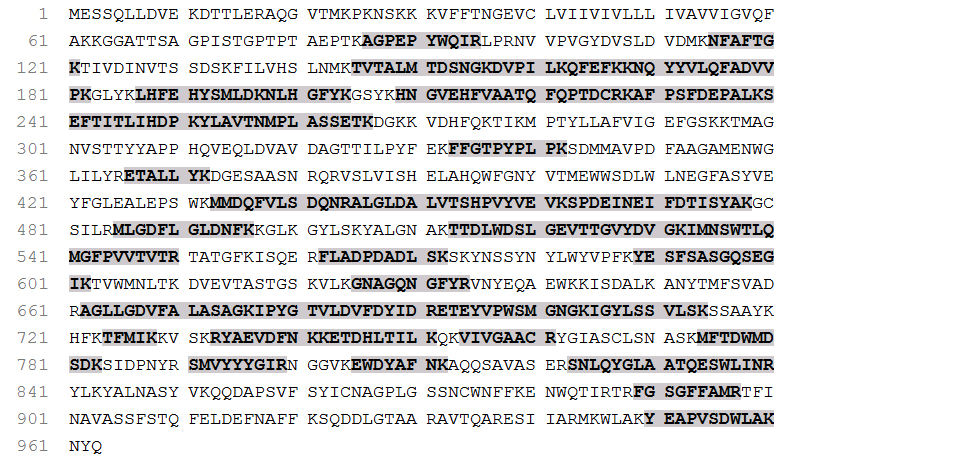


Ccr-25132_c0_g3_i1


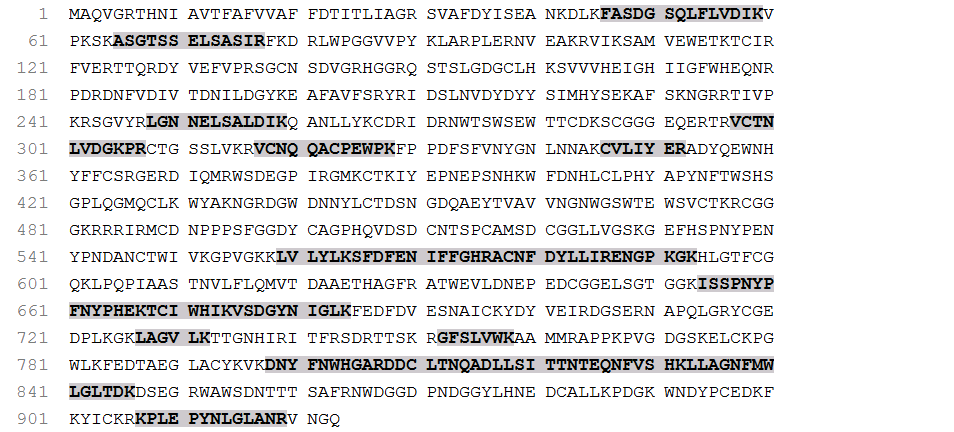


Ccr-25345_c0_g1_i2


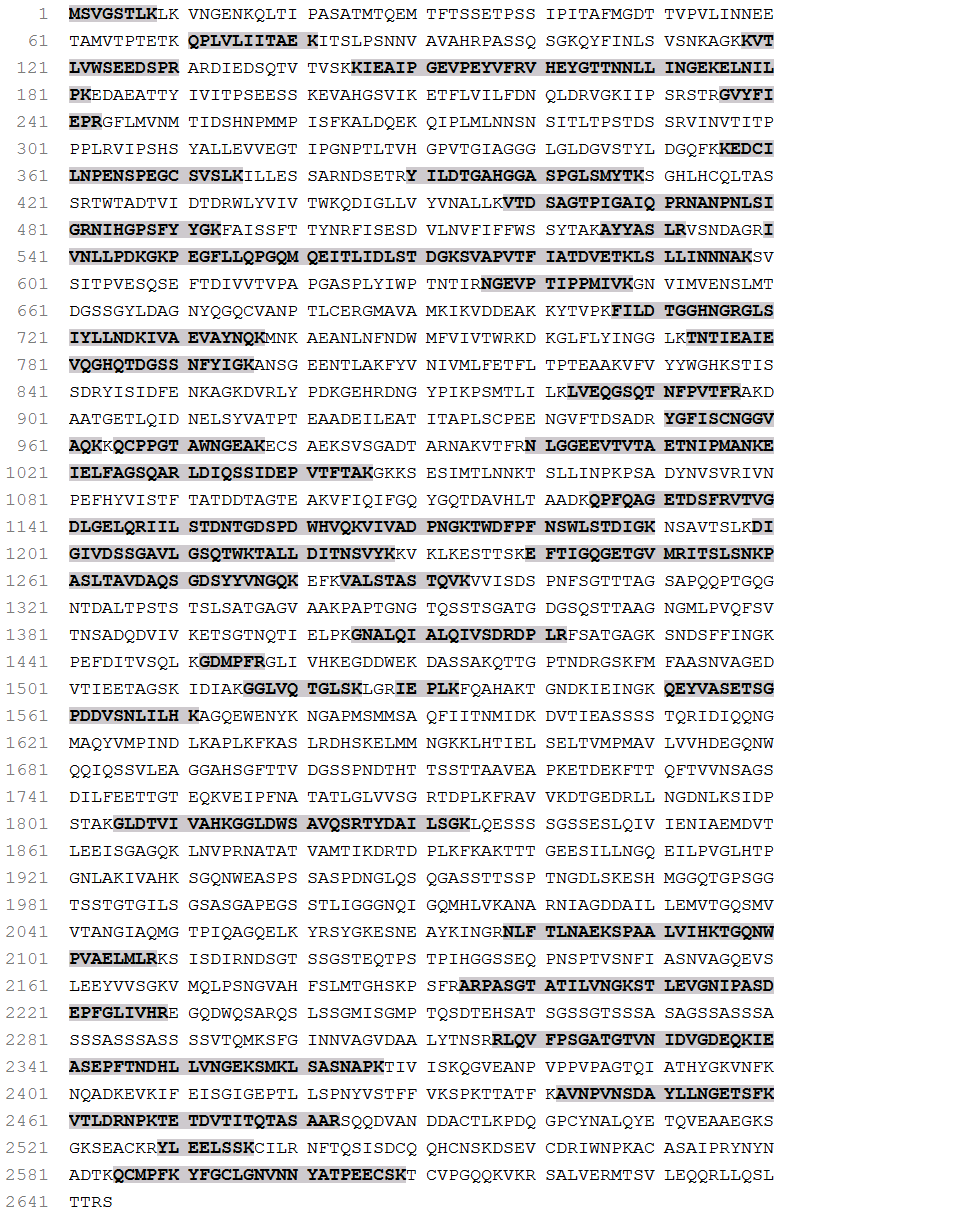


Ccr-25345_c0_g1_i4


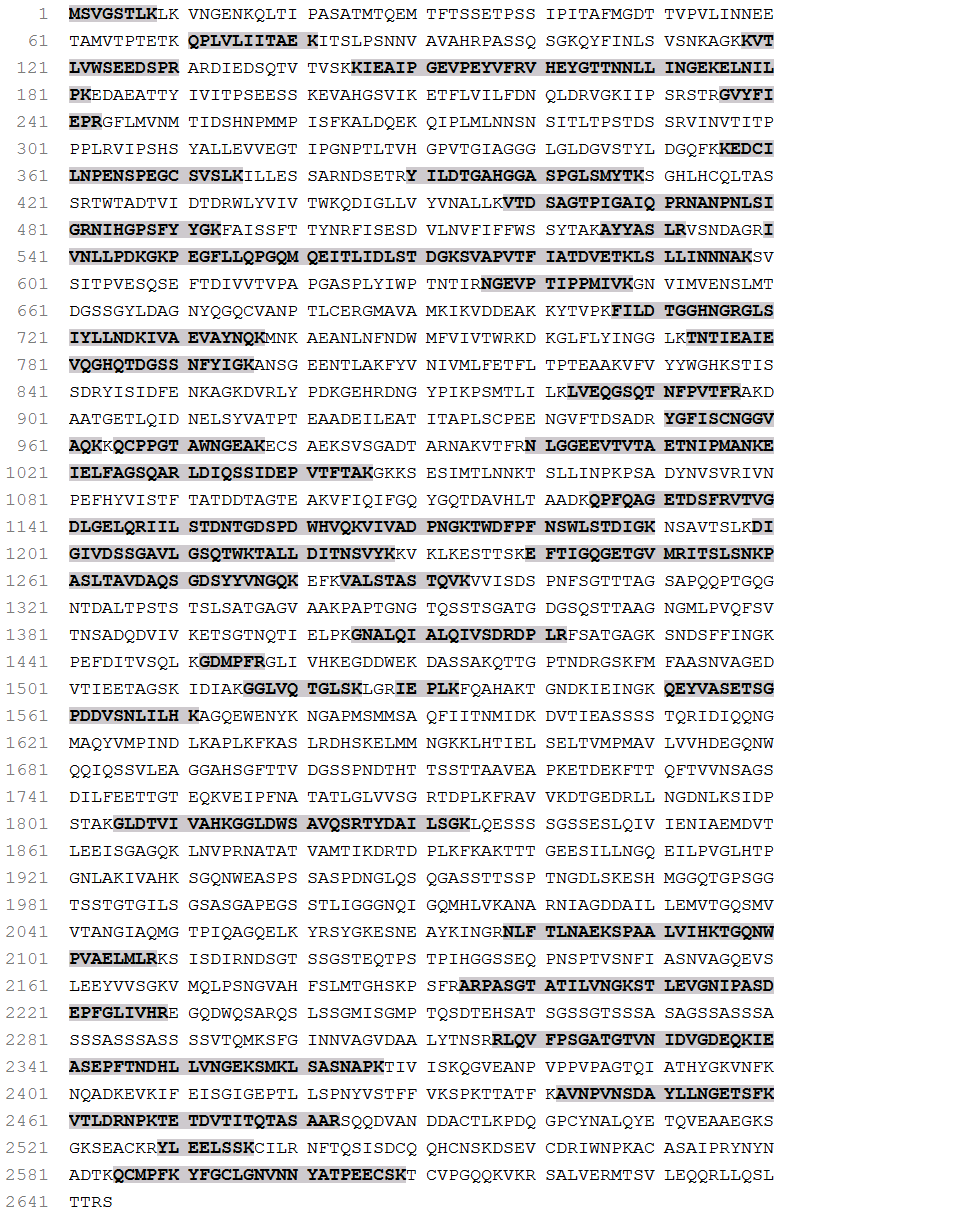


Ccr-25694_c0_g1_i1


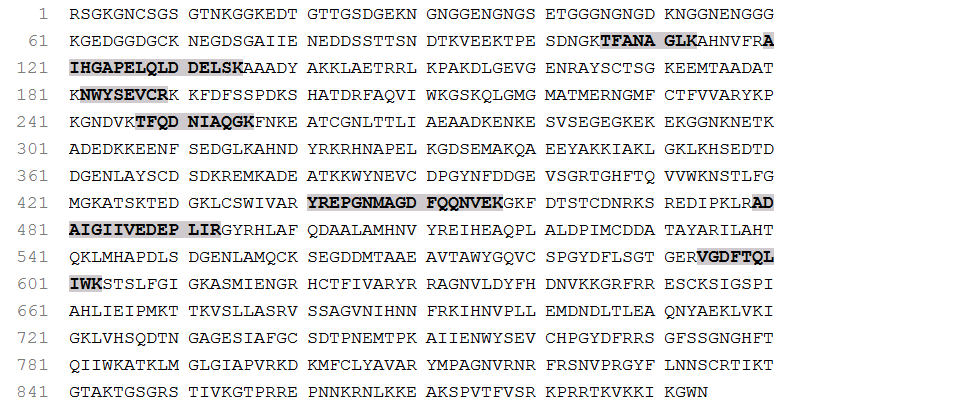


Ccr-25829_c1_g2_i1


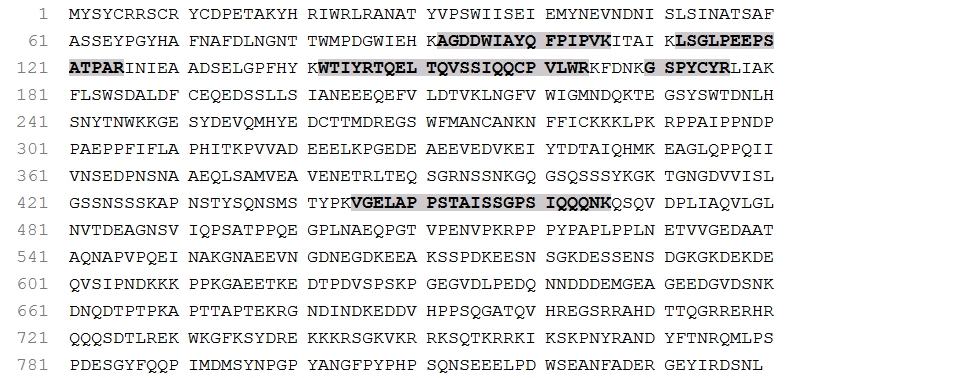


Ccr-25829_c1_g2_i3


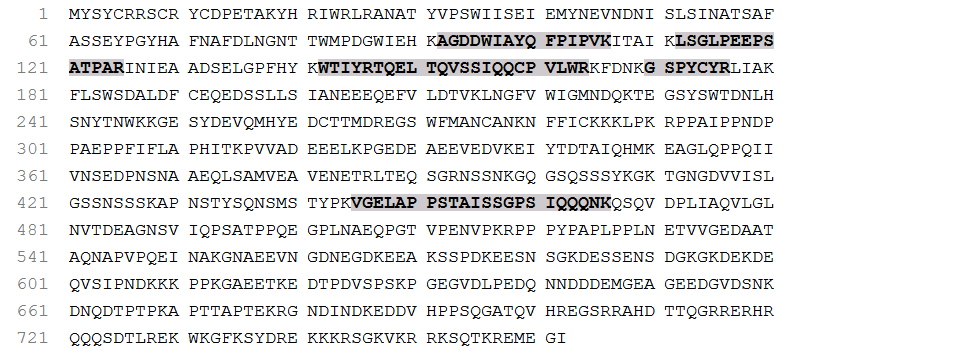


Ccr-26146_c0_g1_i2


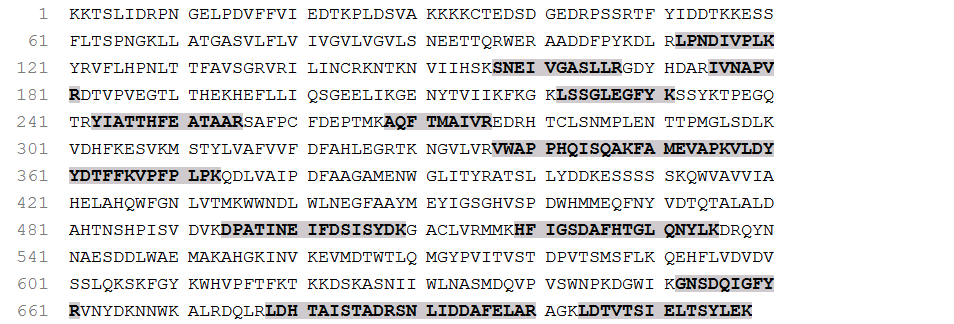


Ccr-26146_c0_g1_i3


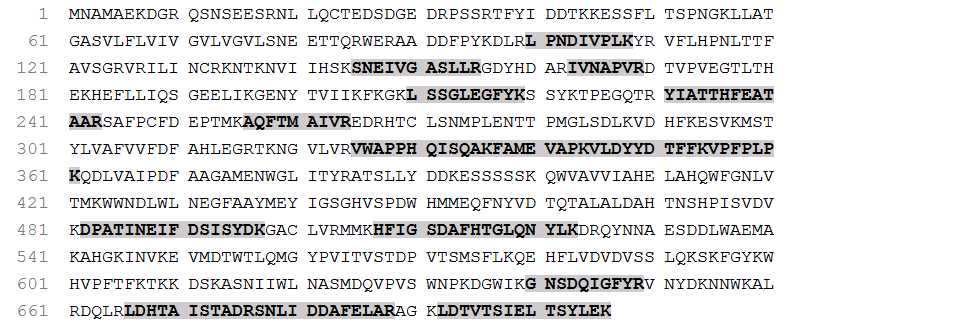


Ccr-26310_c2_g2_i1


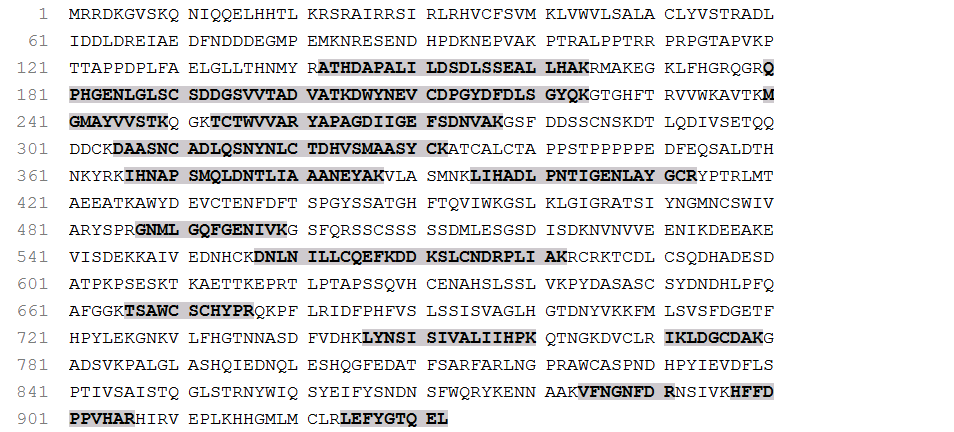


Ccr-26310_c2_g2_i4


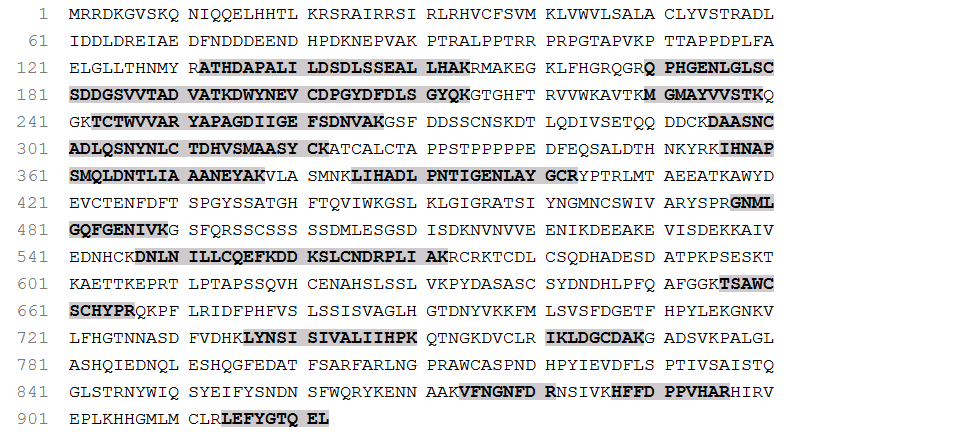


Ccr-26441_c0_g1_i1


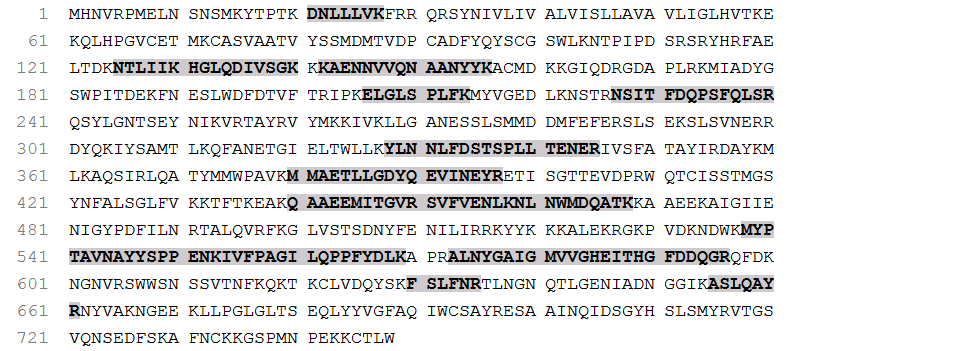


Ccr-26441_c0_g1_i2


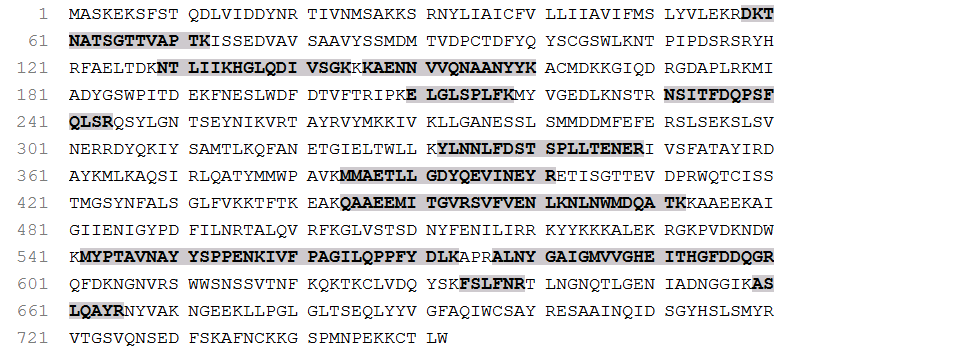


Ccr-26469_c1_g1_i1


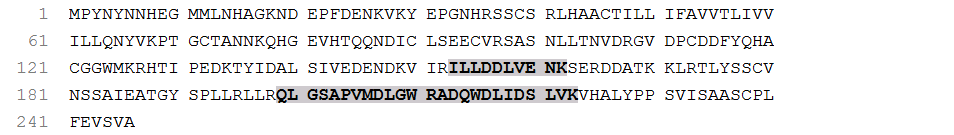


Ccr-26526_c0_g1_i6


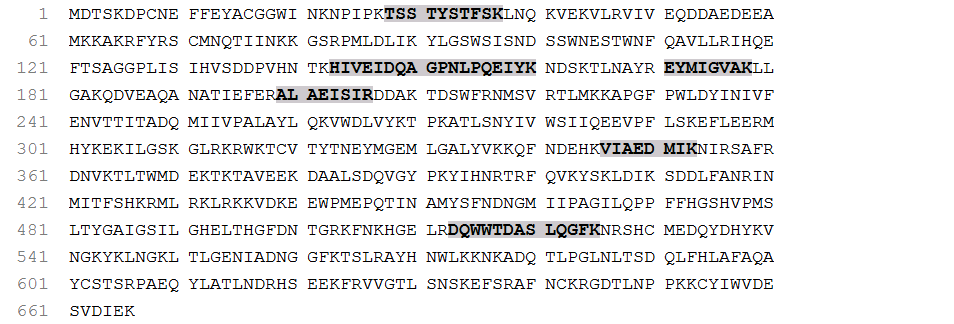


Ccr-26526_c0_g2_i3


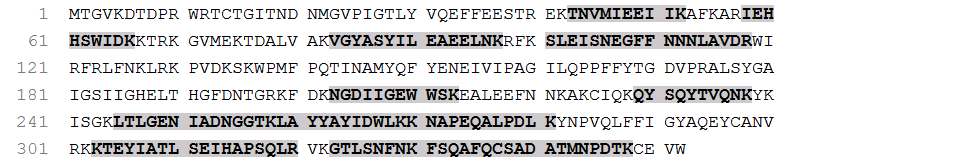


Ccr-26740_c2_g1_i11


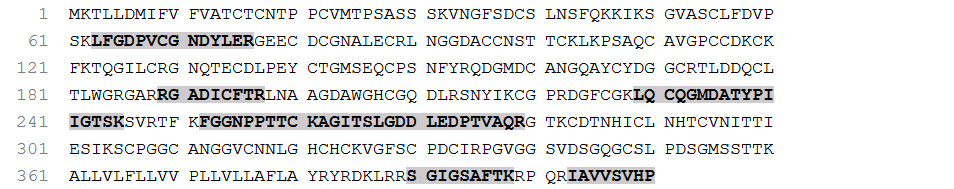


Ccr-27551_c3_g4_i1


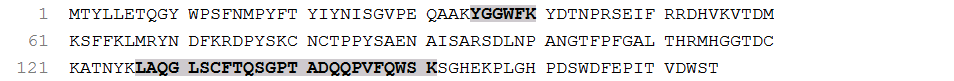


Ccr-27708_c0_g1_i1


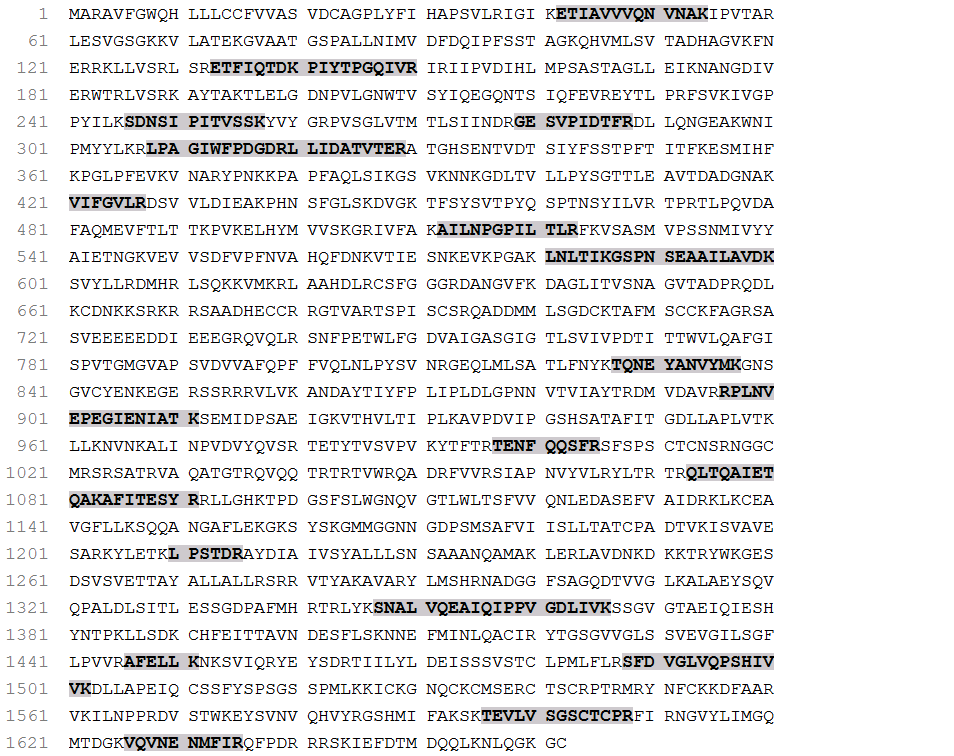


Ccr-28505_c8_g1_i11


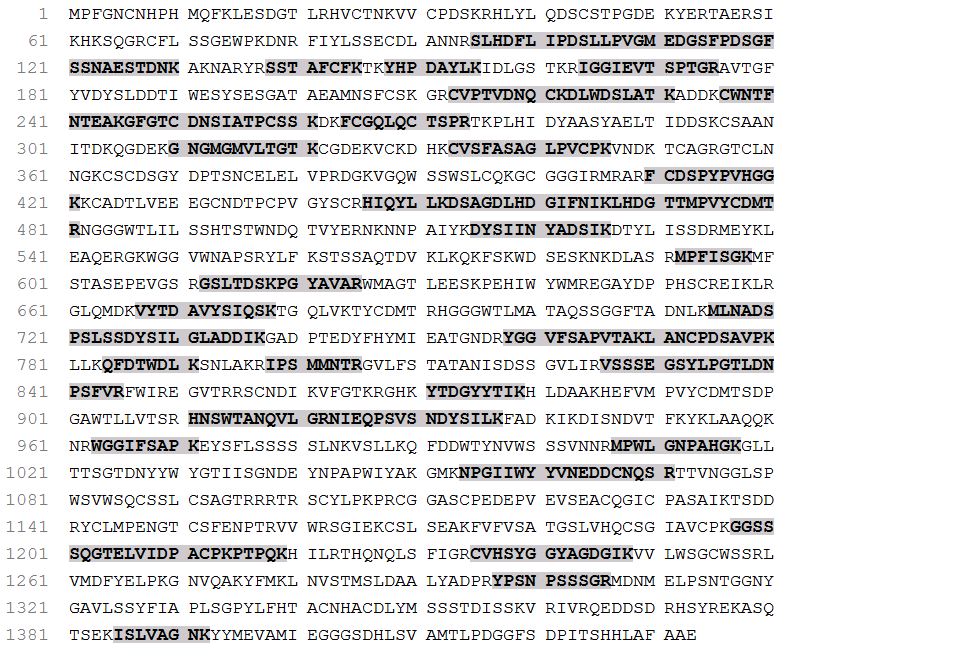


Ccr-28540_c1_g1_i1


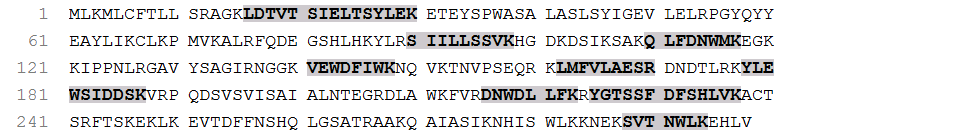


Ccr-14907_c0_g1_i1


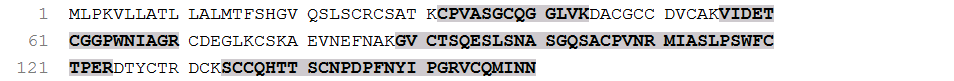


Ccr-15440_c0_g1_i1


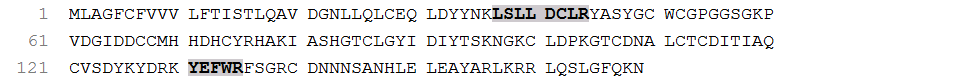


Ccr-17639_c0_g1_i1


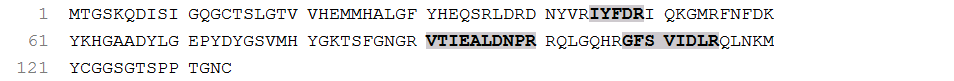


Ccr-17712_c0_g1_i2


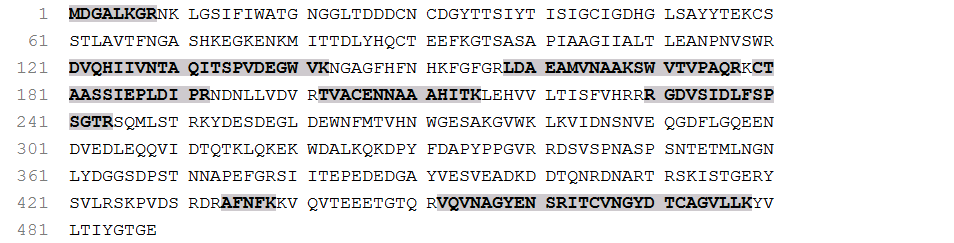


Ccr-19953_c0_g2_i1


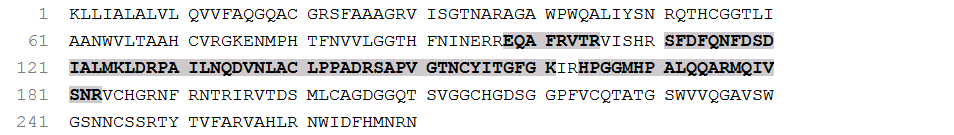


Ccr-20386_c0_g1_i1


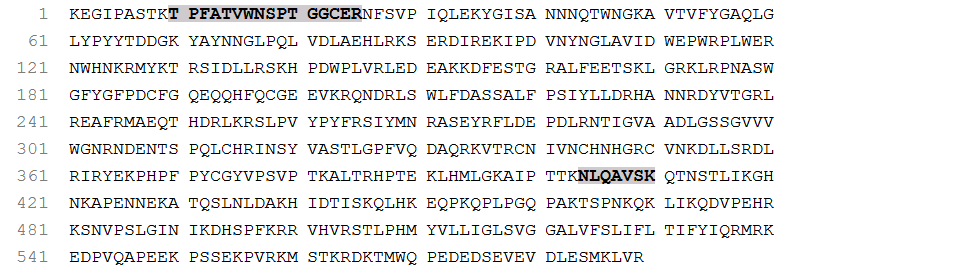


Ccr-20474_c0_g1_i6


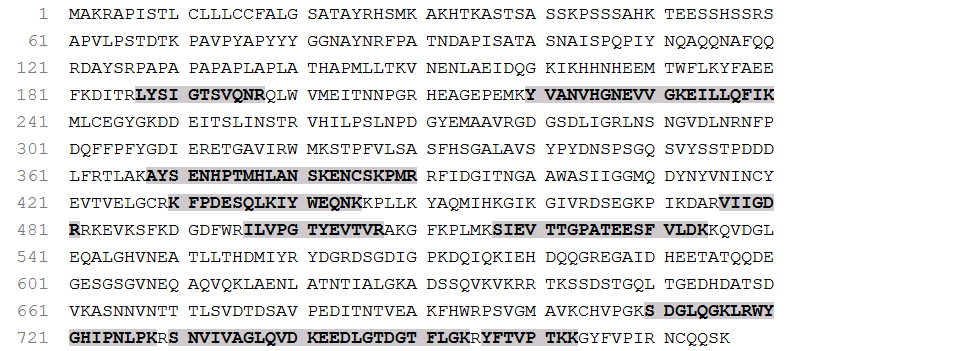


Ccr-21280_c0_g1_i2


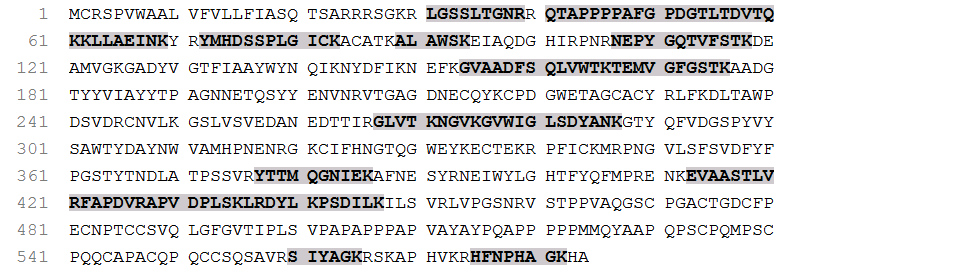


Ccr-21774_c0_g1_i1


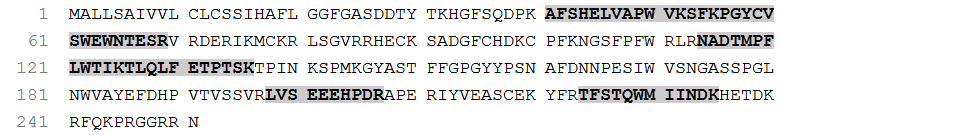


Ccr-22270_c1_g4_i3


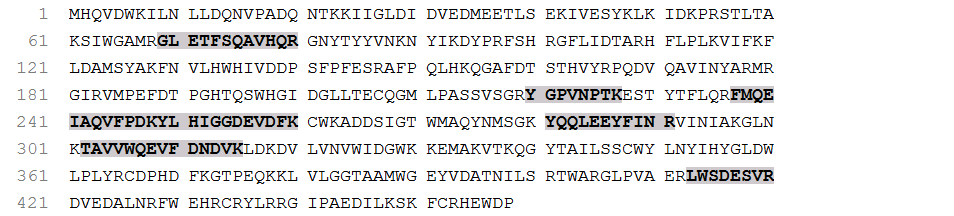


Ccr-22867_c1_g1_i1


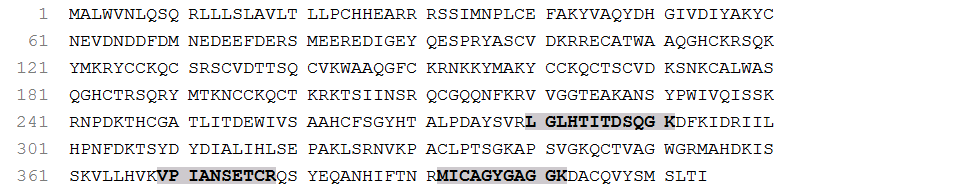


Ccr-22960_c1_g1_i1


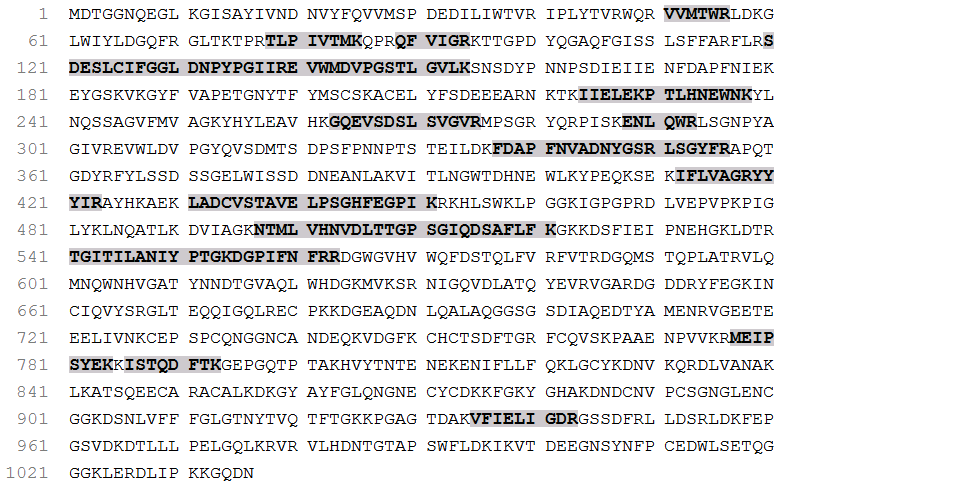


Ccr-22960_c1_g1_i2


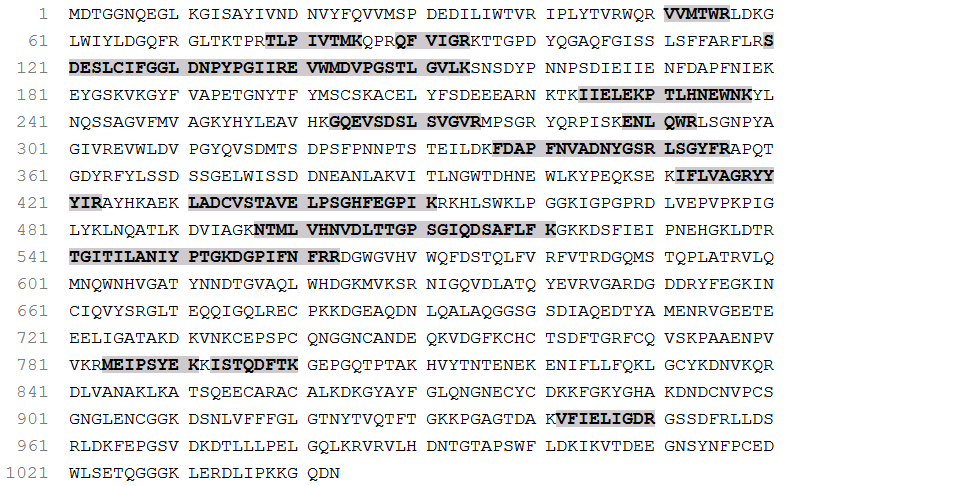


Ccr-23410_c1_g3_i3


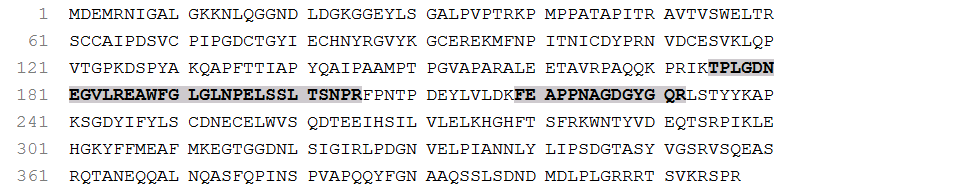


Ccr-23859_c2_g1_i6


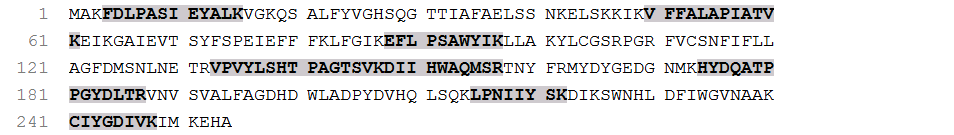


Ccr-24923_c0_g1_i2


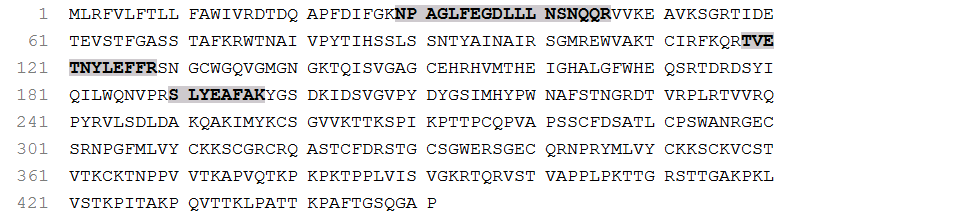


Ccr-24923_c0_g1_i3


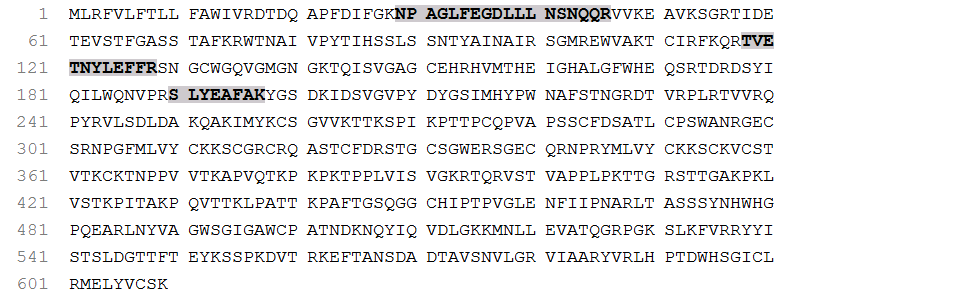


Ccr-25236_c0_g1_i1


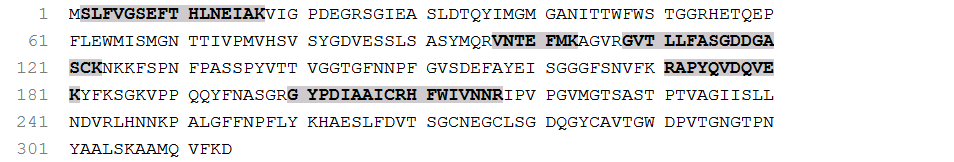


Ccr-25236_c0_g1_i2


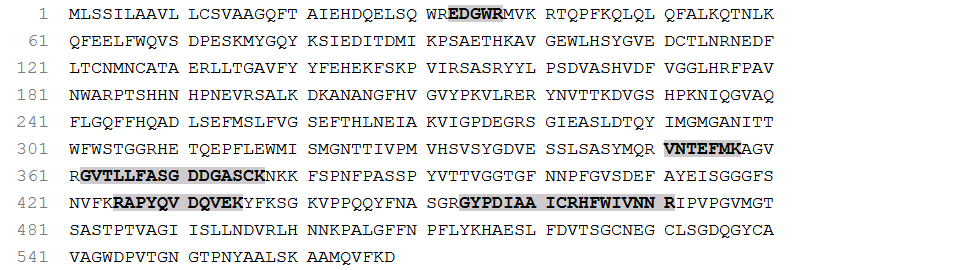


Ccr-25821_c0_g1_i2


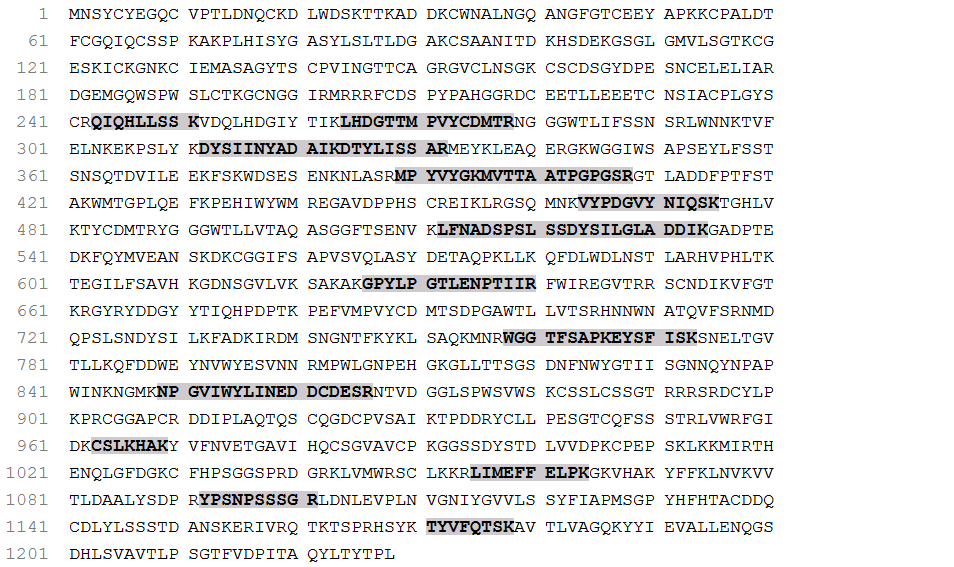


Ccr-25825_c2_g2_i1


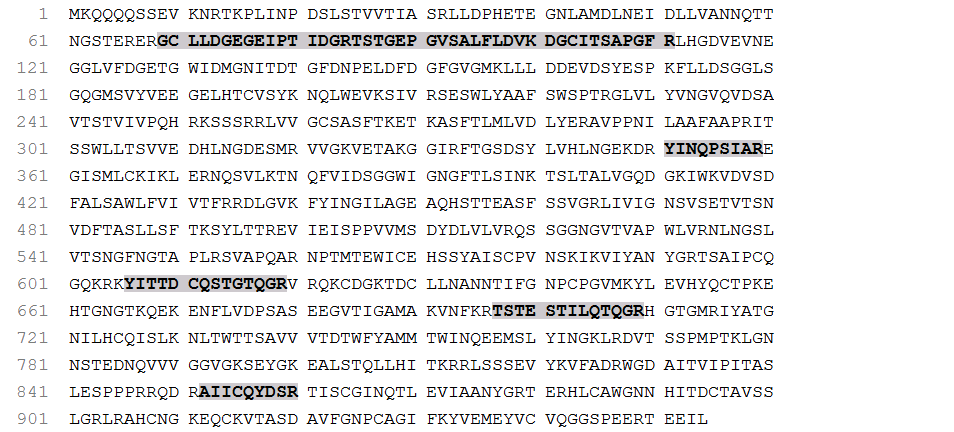


Ccr-26049_c0_g1_i6


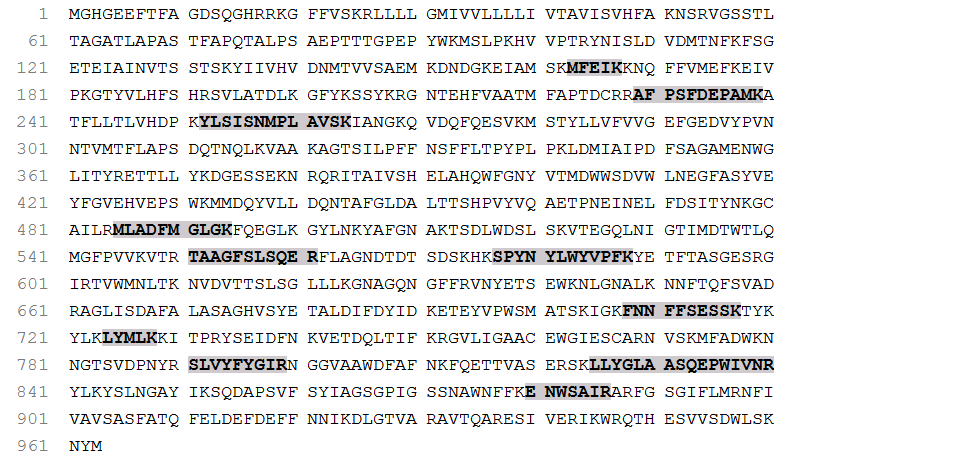


Ccr-26072_c0_g1_i6


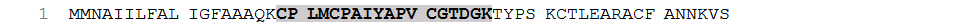


Ccr-26072_c0_g1_i7


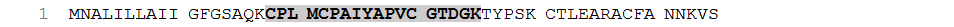


Ccr-26153_c0_g1_i4


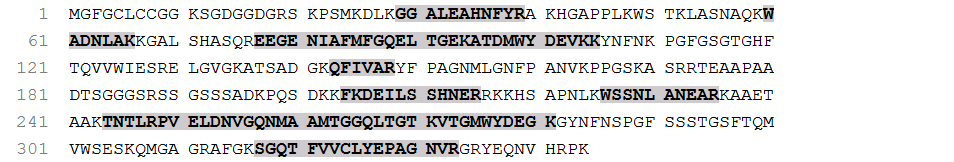


Ccr-26432_c1_g2_i4


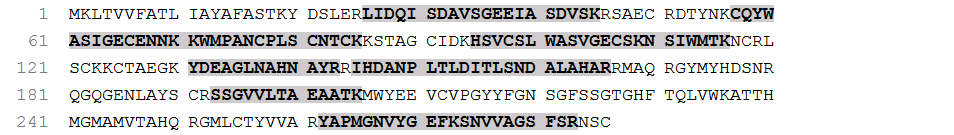


Ccr-26526_c0_g1_i13


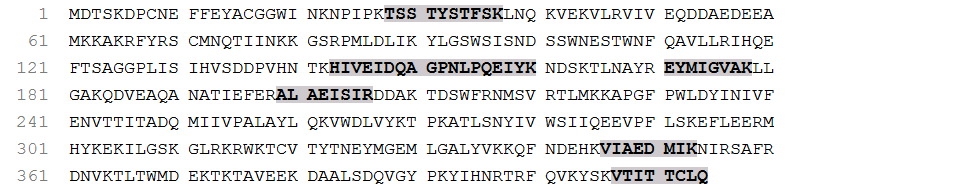


Ccr-26526_c0_g1_i7


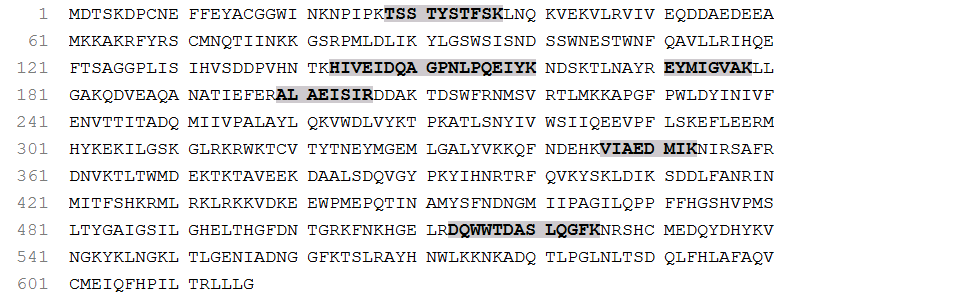


Ccr-26776_c2_g1_i2


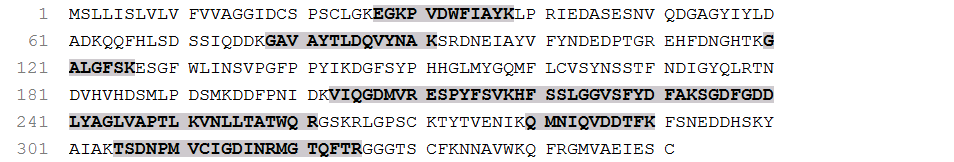


Ccr-27169_c0_g2_i11


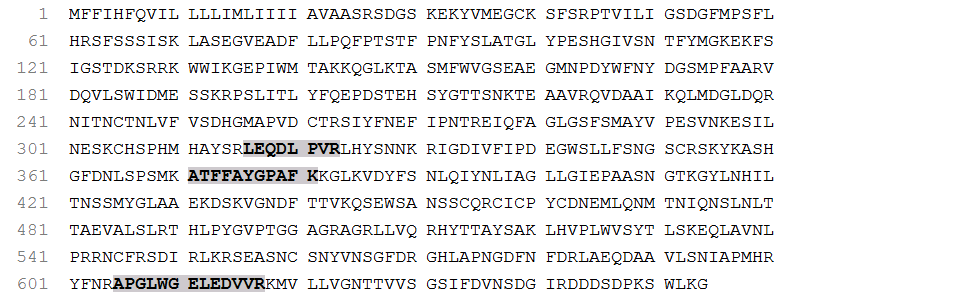


Ccr-27169_c0_g2_i12


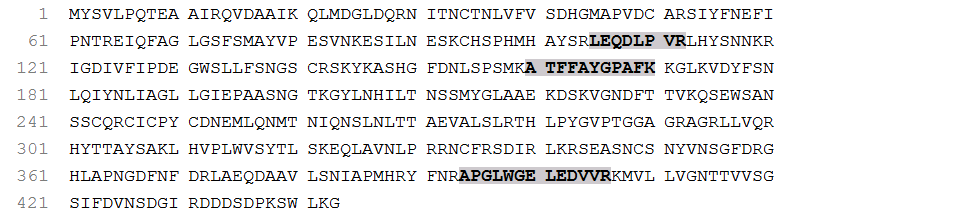


Ccr-27169_c0_g2_i3


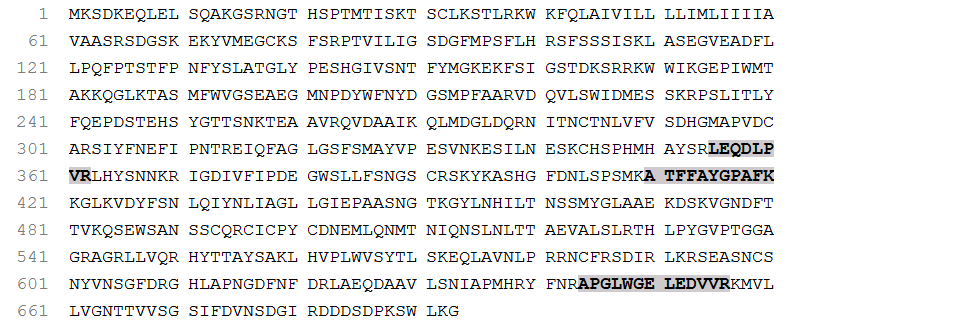


Ccr-27719_c1_g2_i1


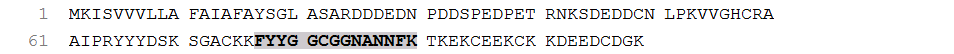


Ccr-28128_c2_g2_i2


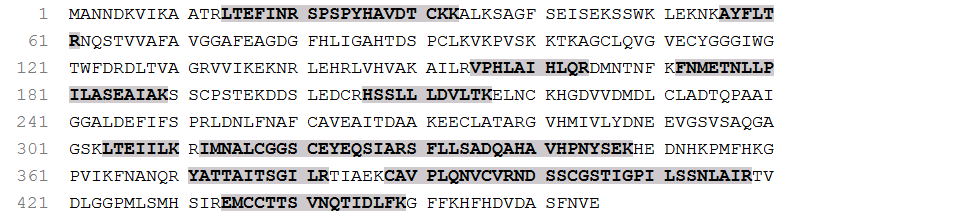


Ccr-28505_c8_g2_i5


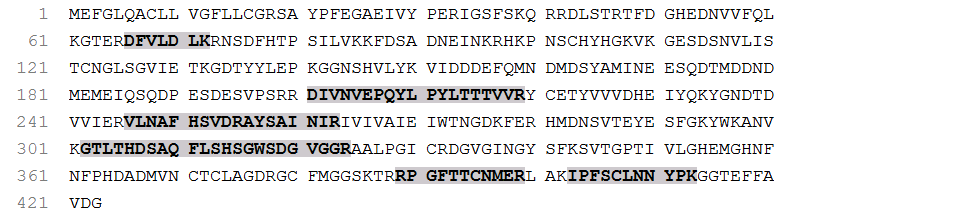


Ccr-8523_c0_g1_i1


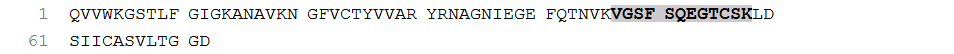


Myx-42630_c0_g1_i4


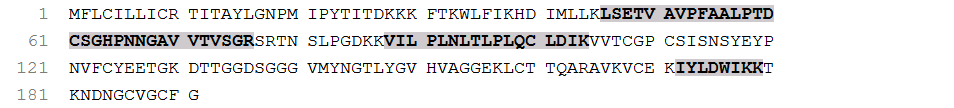


Myx-42946_c0_g1_i3


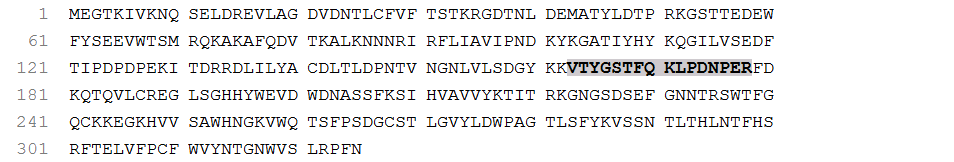


Phy-24914_c1_g1_i5


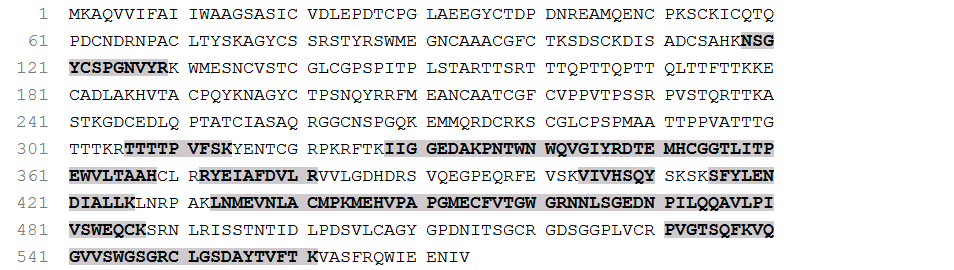


Phy-43066_c0_g1_i1


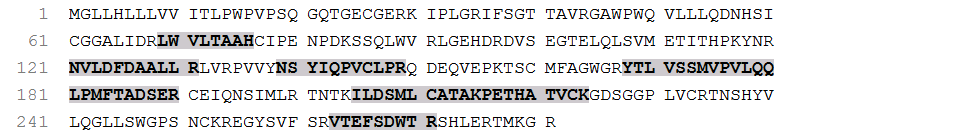


Phy-9231_c0_g1_i1


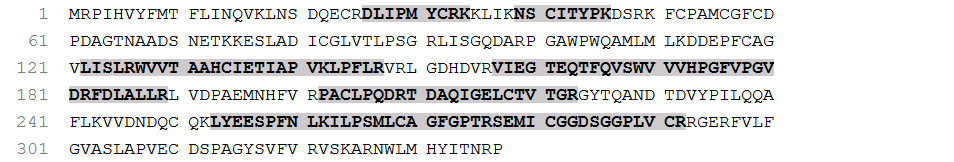


Phy-17519_c0_g1_i1


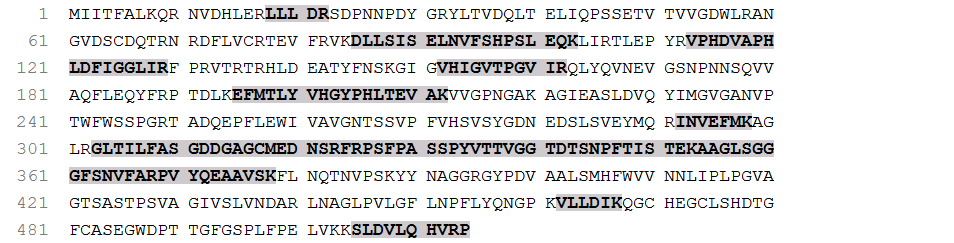


Phy-24812_c0_g1_i4


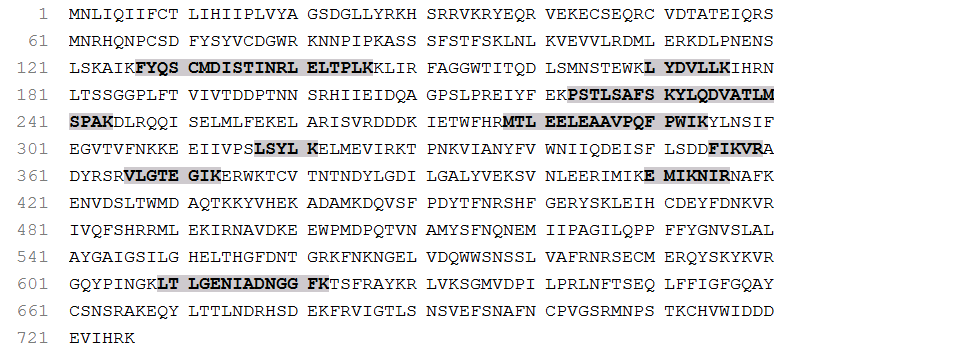


Phy-26874_c0_g1_i4


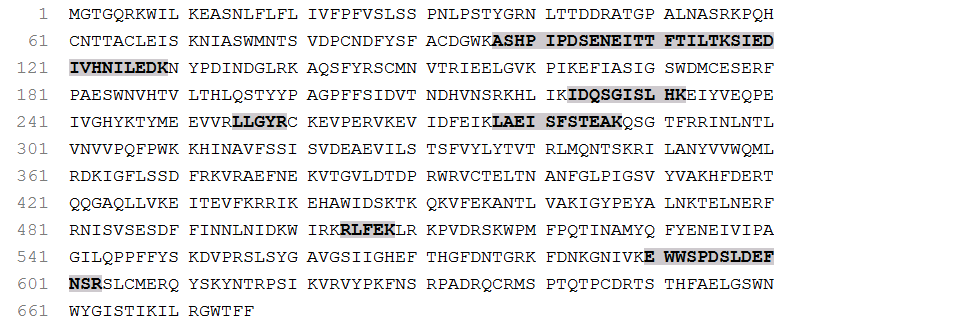


Phy-24812_c0_g1_i11


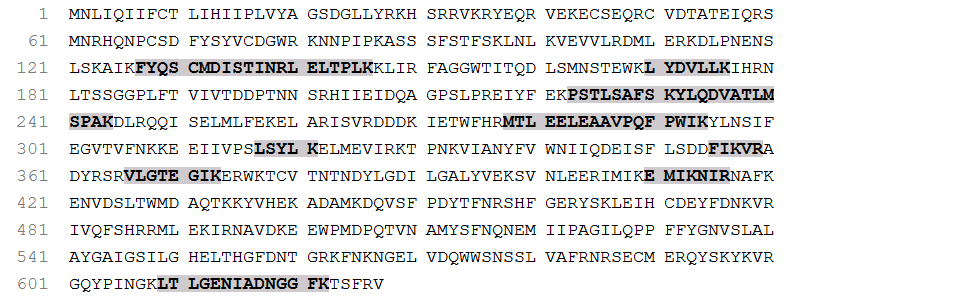


Phy-26874_c0_g1_i8


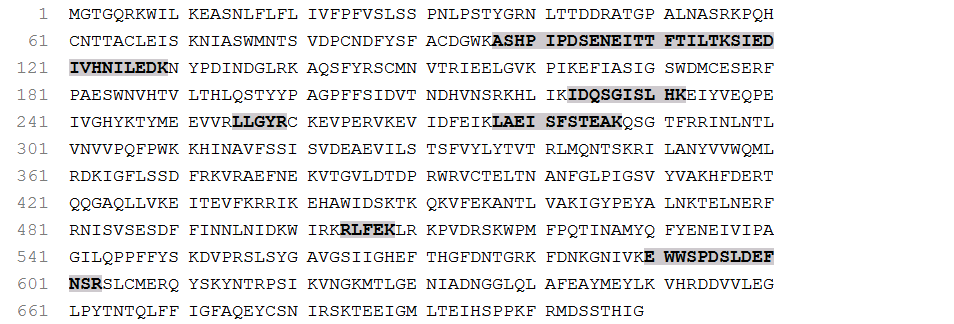


Phy-26874_c0_g1_i7


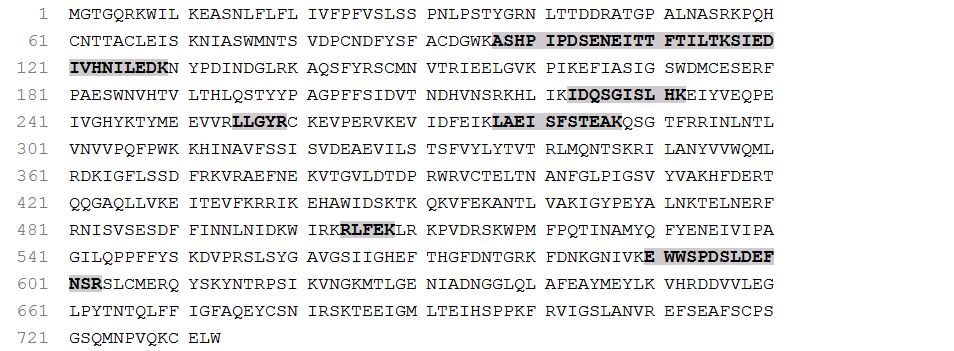


Phy-25018_c0_g1_i7


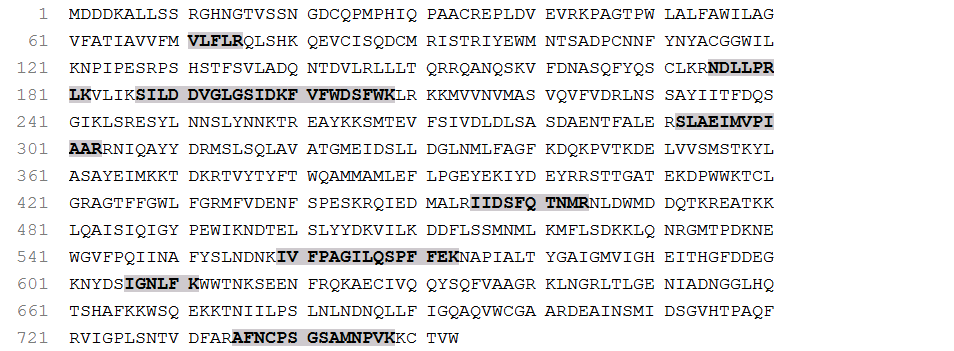


Phy-24539_c0_g2_i19


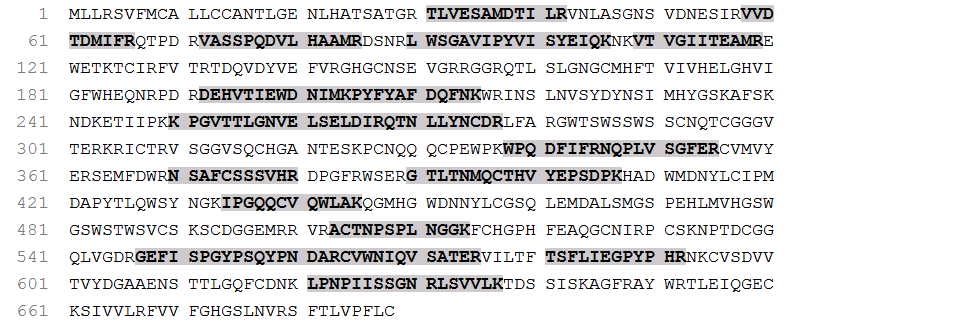


Phy-25249_c1_g3_i12


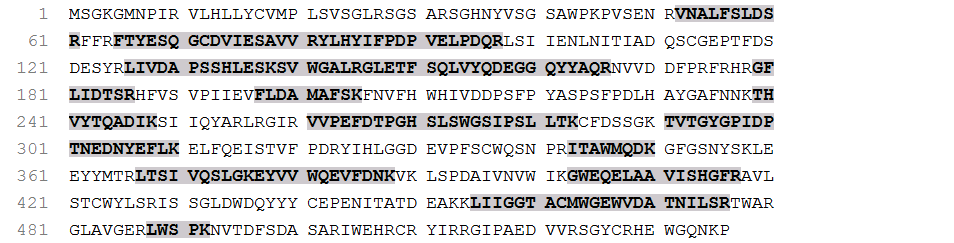


Phy-25249_c1_g3_i18


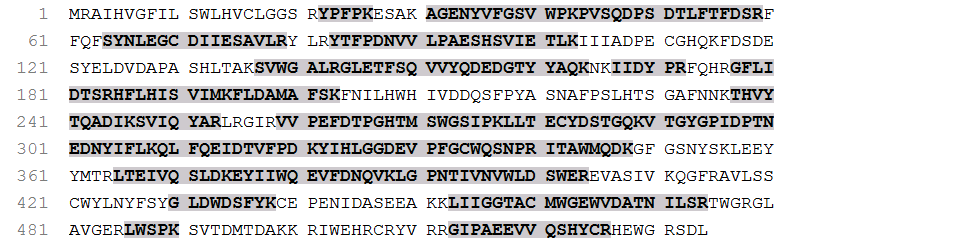


Phy-25249_c1_g3_i20


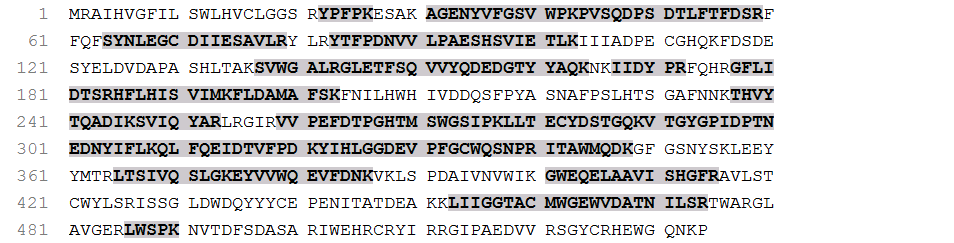


Phy-23951_c0_g1_i2


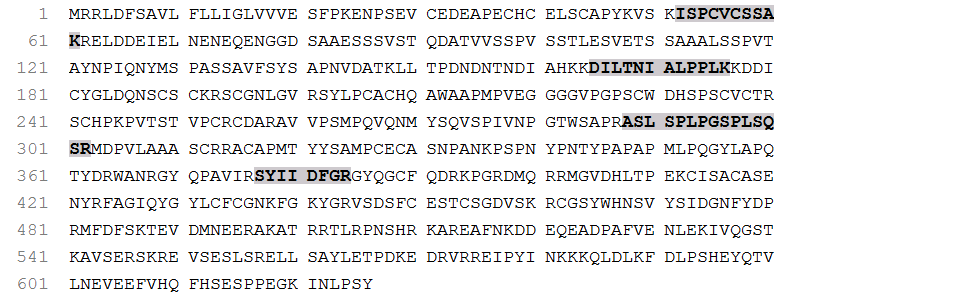


Phy-23951_c0_g1_i7


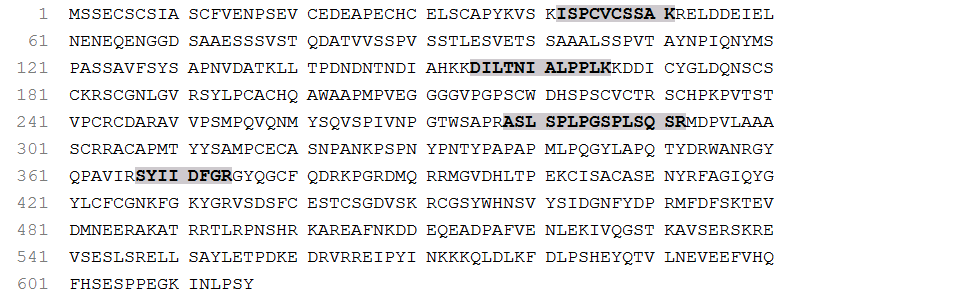


Phy-26261_c0_g1_i1


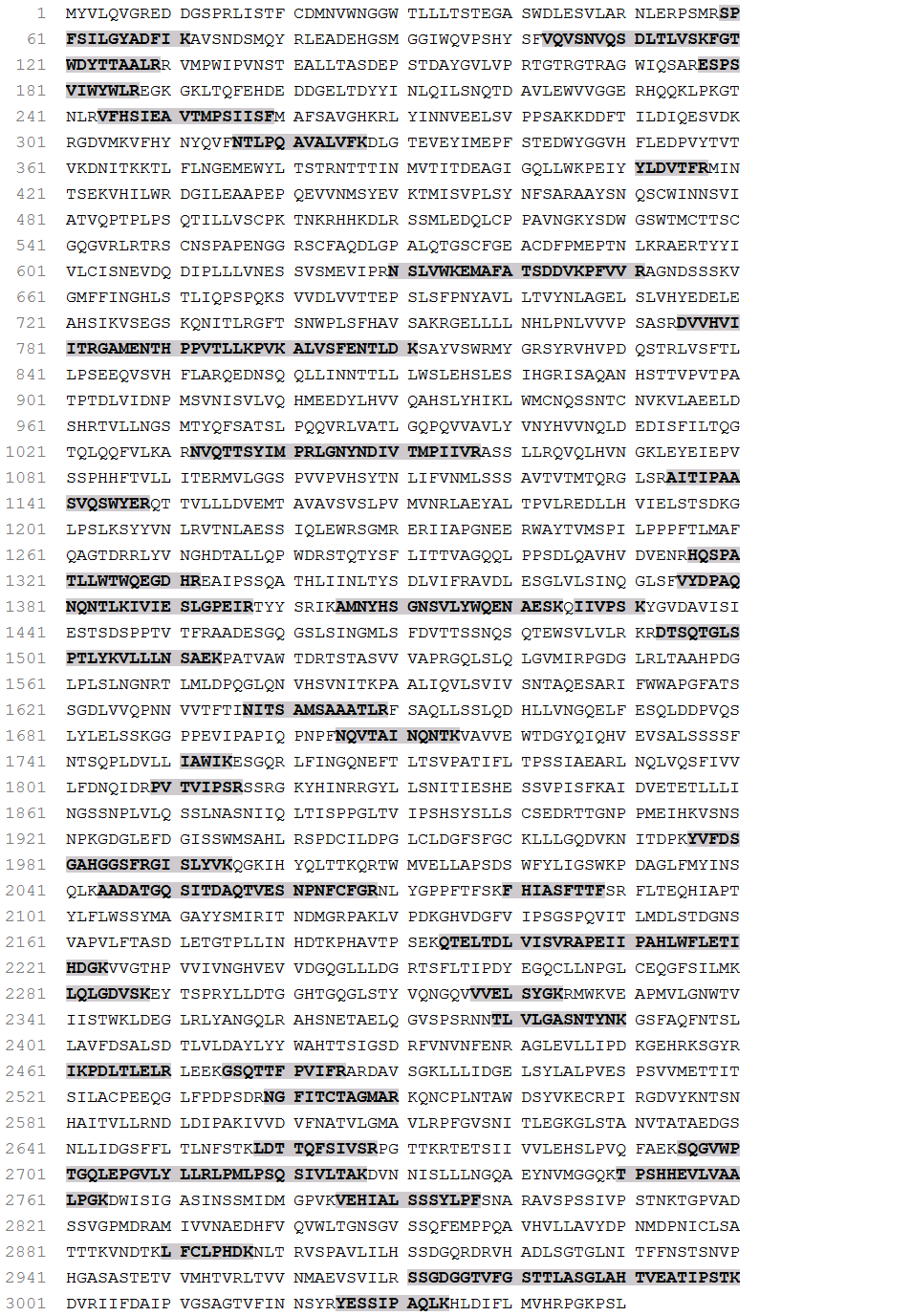


Phy-25185_c14_g1_i6


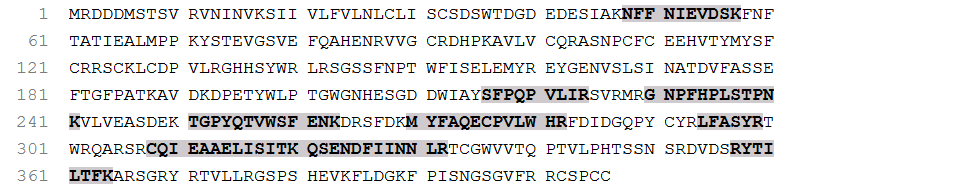


Phy-20553_c0_g1_i1


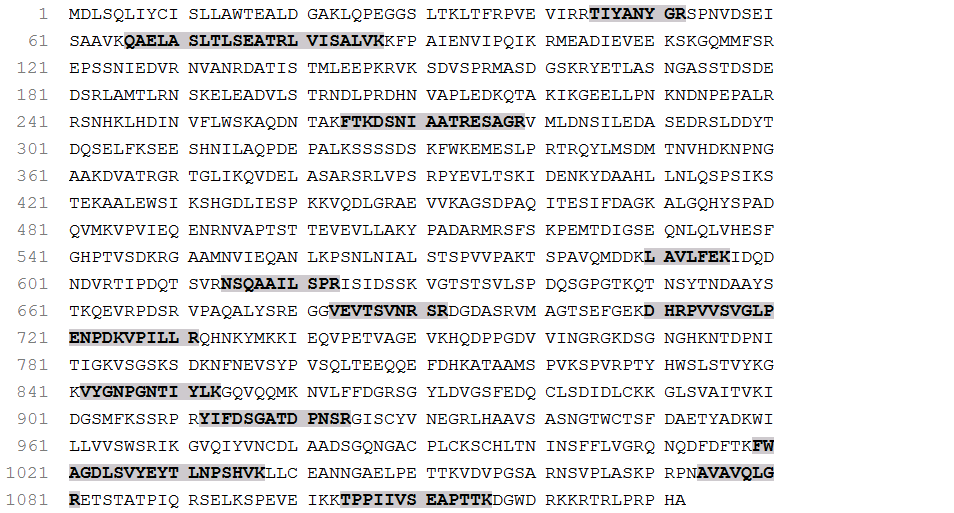


Phy-24539_c0_g2_i6


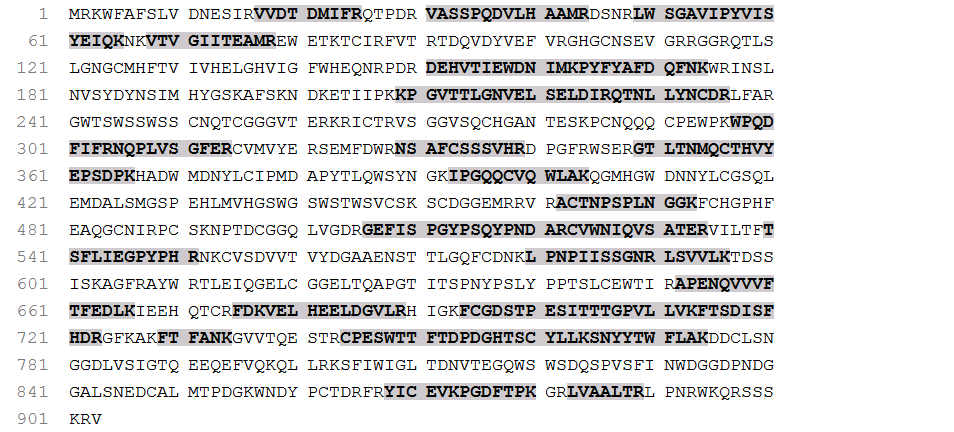


Phy-24539_c0_g2_i8


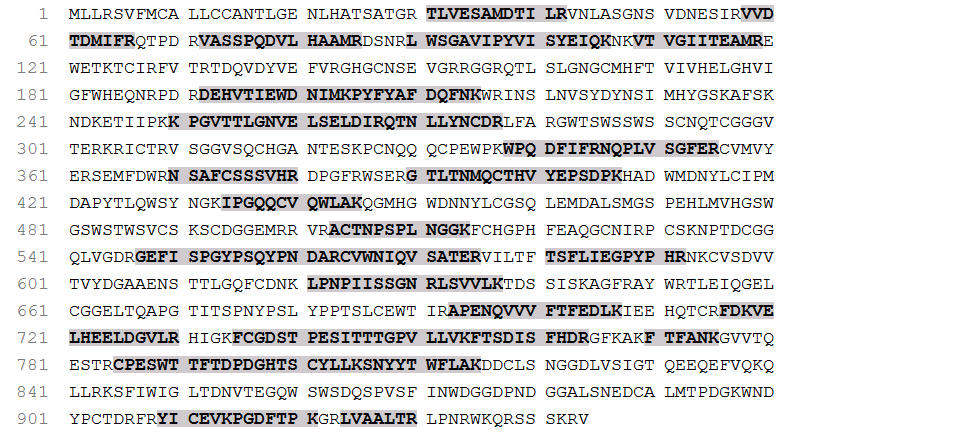


Phy-24539_c0_g1_i15


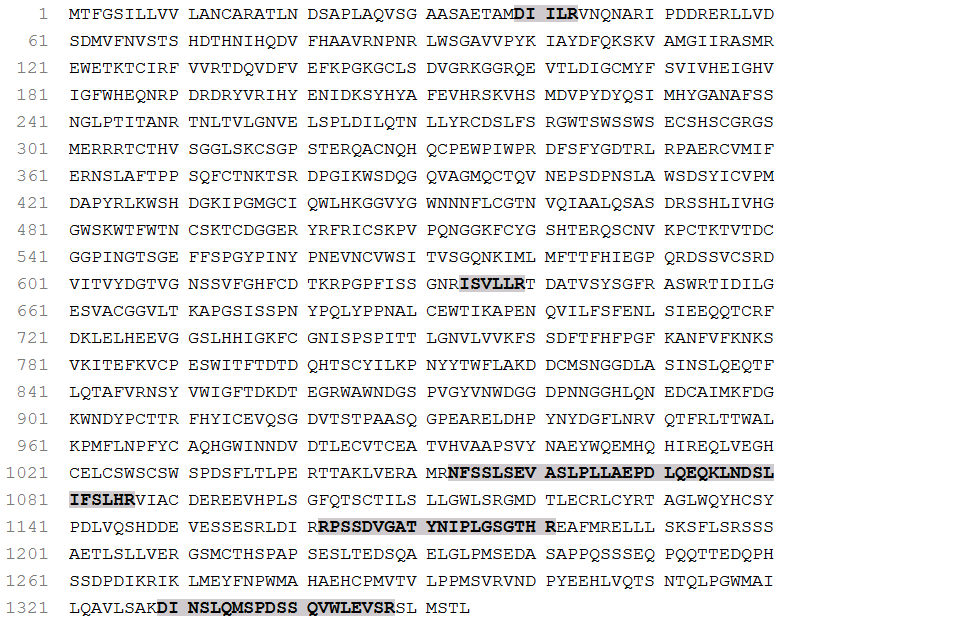


Phy-25185_c14_g1_i9


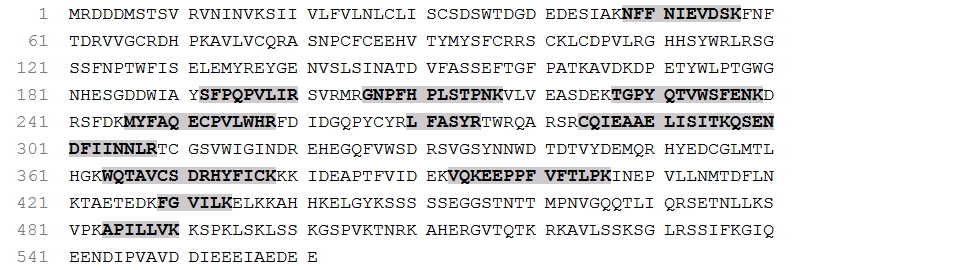


Phy-25185_c14_g1_i13


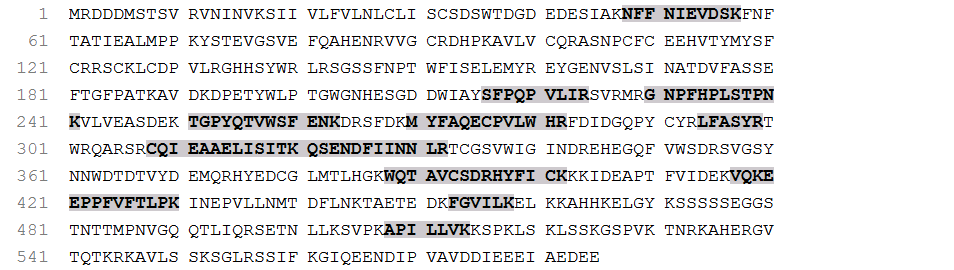


Phy-25443_c2_g3_i1


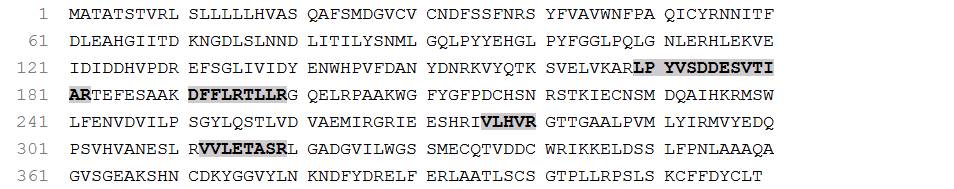


Phy-25443_c2_g3_i2


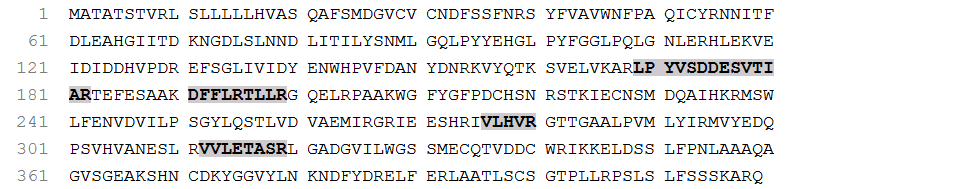


Phy-25624_c2_g4_i2


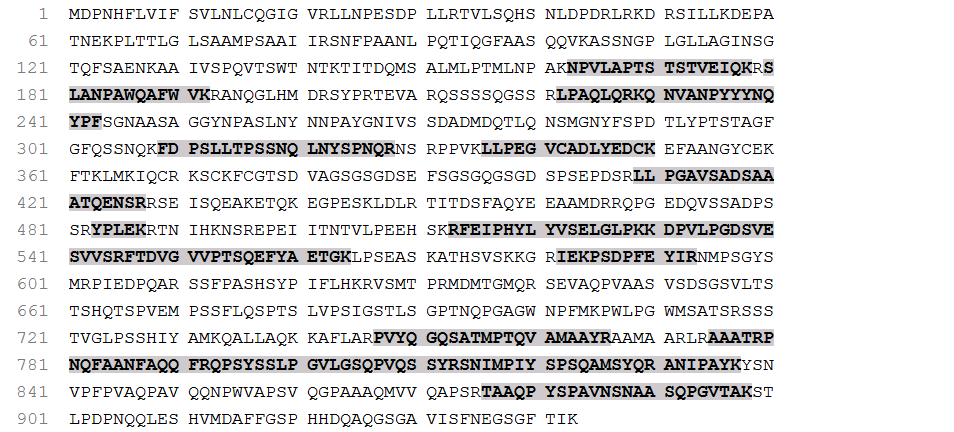


Phy-16682_c0_g1_i1


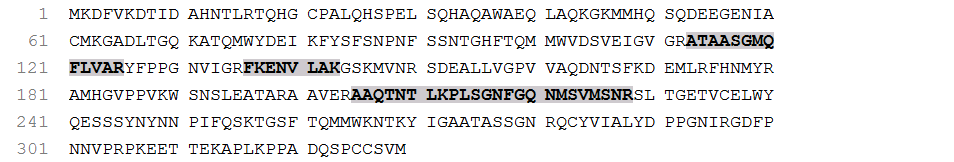


Phy-23555_c0_g1_i1


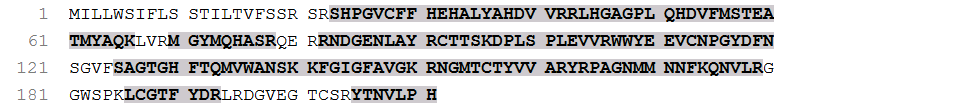


Phy-27031_c2_g1_i16


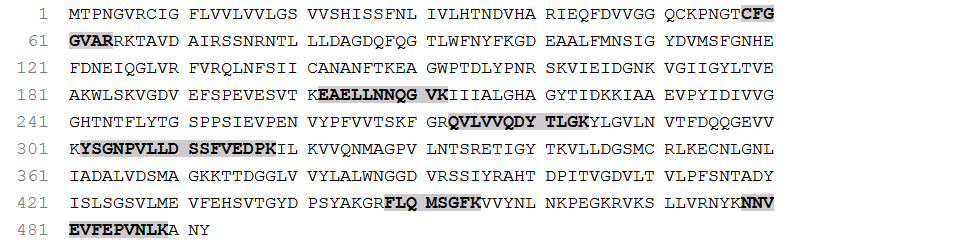


Phy-27031_c2_g1_i9

Phy-24372_c1_g9_i1

Phy-25841_c4_g1_i11

Phy-24914_c1_g6_i1

Phy-24914_c1_g1_i6

Phy-26587_c3_g2_i8

Phy-20919_c0_g1_i2

Phy-26681_c3_g2_i3

Phy-25410_c0_g1_i7

Phy-25410_c0_g1_i15

Phy-24578_c4_g1_i2

Phy-26489_c1_g1_i15

Phy-26489_c1_g1_i12

Phy-26489_c1_g1_i10

Phy-26489_c1_g1_i9

Phy-26489_c1_g2_i4

Phy-24578_c4_g1_i7

Phy-26489_c1_g1_i39

Phy-24825_c0_g1_i6

Phy-24825_c0_g1_i1

Phy-24825_c0_g1_i10

Phy-24825_c0_g1_i3

Phy-24825_c0_g1_i2

Phy-24825_c0_g1_i7

Phy-3110_c0_g1_i2

Phy-25018_c0_g1_i21

Phy-25690_c5_g1_i19

Phy-25690_c5_g1_i16

Phy-25262_c5_g2_i3

Phy-23951_c0_g1_i1

Phy-23951_c0_g1_i5

Phy-23951_c0_g1_i3

Phy-26681_c3_g3_i2

Phy-26034_c6_g2_i6

Phy-25492_c4_g1_i4

Phy-26034_c6_g2_i7

Phy-26046_c2_g1_i5

Phy-26046_c2_g1_i2

Phy-25841_c4_g1_i8

Phy-25841_c4_g1_i2

Phy-25841_c4_g1_i14

Phy-24865_c3_g1_i1

Phy-24865_c3_g1_i2

Phy-22952_c0_g1_i1

Phy-22952_c0_g1_i2

Phy-34426_c0_g1_i1

Phy-26261_c0_g1_i8
