# Supplementary material for: Recruitment of toxin-like proteins with ancestral venom function supports endoparasitic lifestyles of Myxozoa
Source: PeerJ. 2021 Apr 26;9:e11208. doi: 10.7717/peerj.11208 (PMC8083181; doi:10.7717/peerj.11208)
Supplement: Supplemental Information 8 — Percentage of transcripts with MS/MS spectral matches vs percentage toxin transcripts with MS/MS spectral matches. Global MS/MS matches range from ~3.5-6%, however the subset of toxins with MS/MS greatly differs from 2-3.5% in parasitic vs free-living cnidarians. [file peerj-09-11208-s008.pptx]

## Slide 1
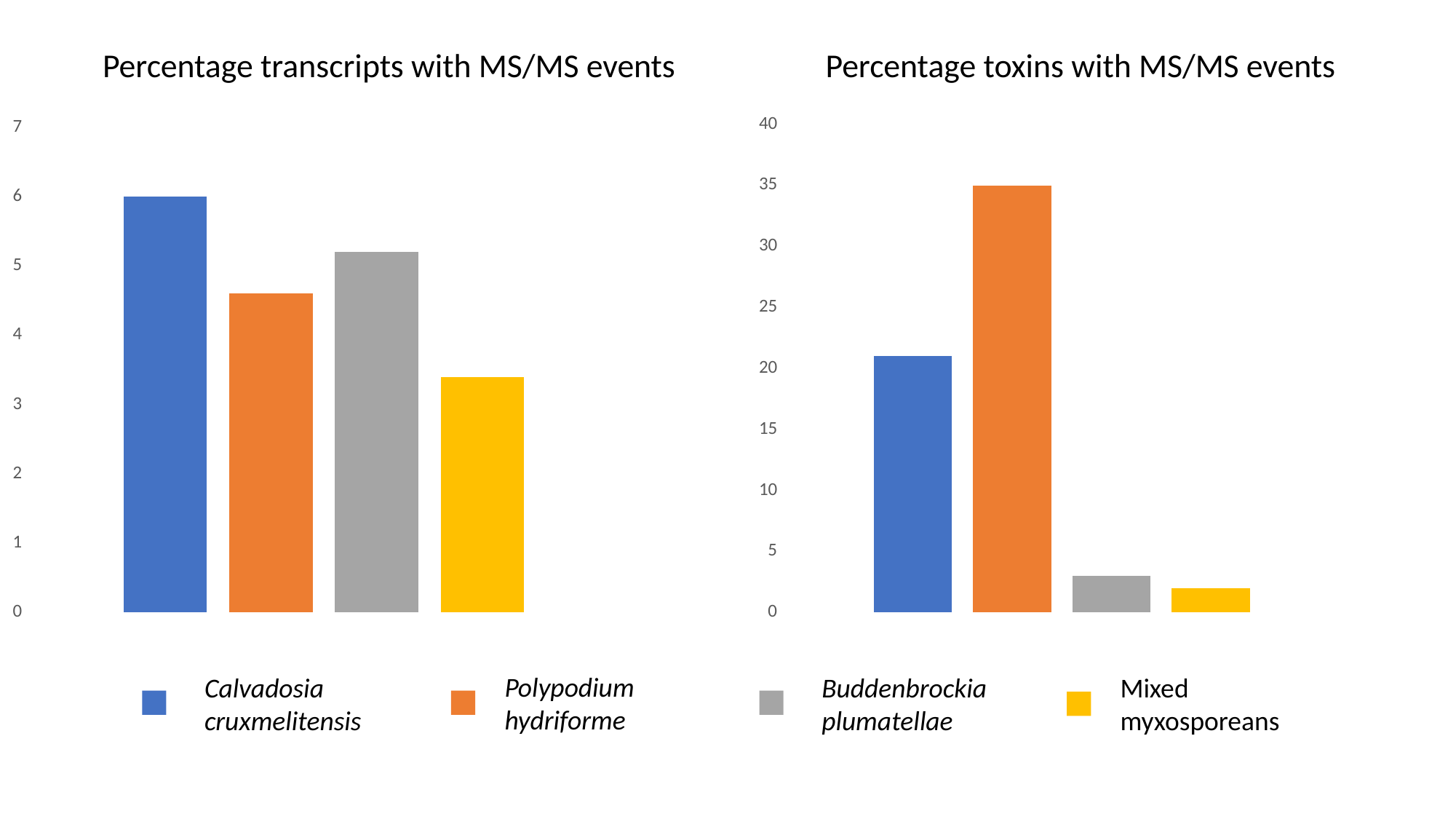

Percentage toxins with MS/MS events
Percentage transcripts with MS/MS events
### Chart
| Category | C. crux | P. hydr | B.plum | Mixed myx |
|---|---|---|---|---|
| Category 1 | 21.0 | 35.0 | 3.0 | 2.0 |
### Chart
| Category | C. crux | P. hydr | B.plum | Mixed myx |
|---|---|---|---|---|
| Category 1 | 6.0 | 4.6 | 5.2 | 3.4 |Polypodium
hydriforme
Calvadosia
cruxmelitensis
Buddenbrockia
plumatellae
Mixed
myxosporeans

## Slide 2
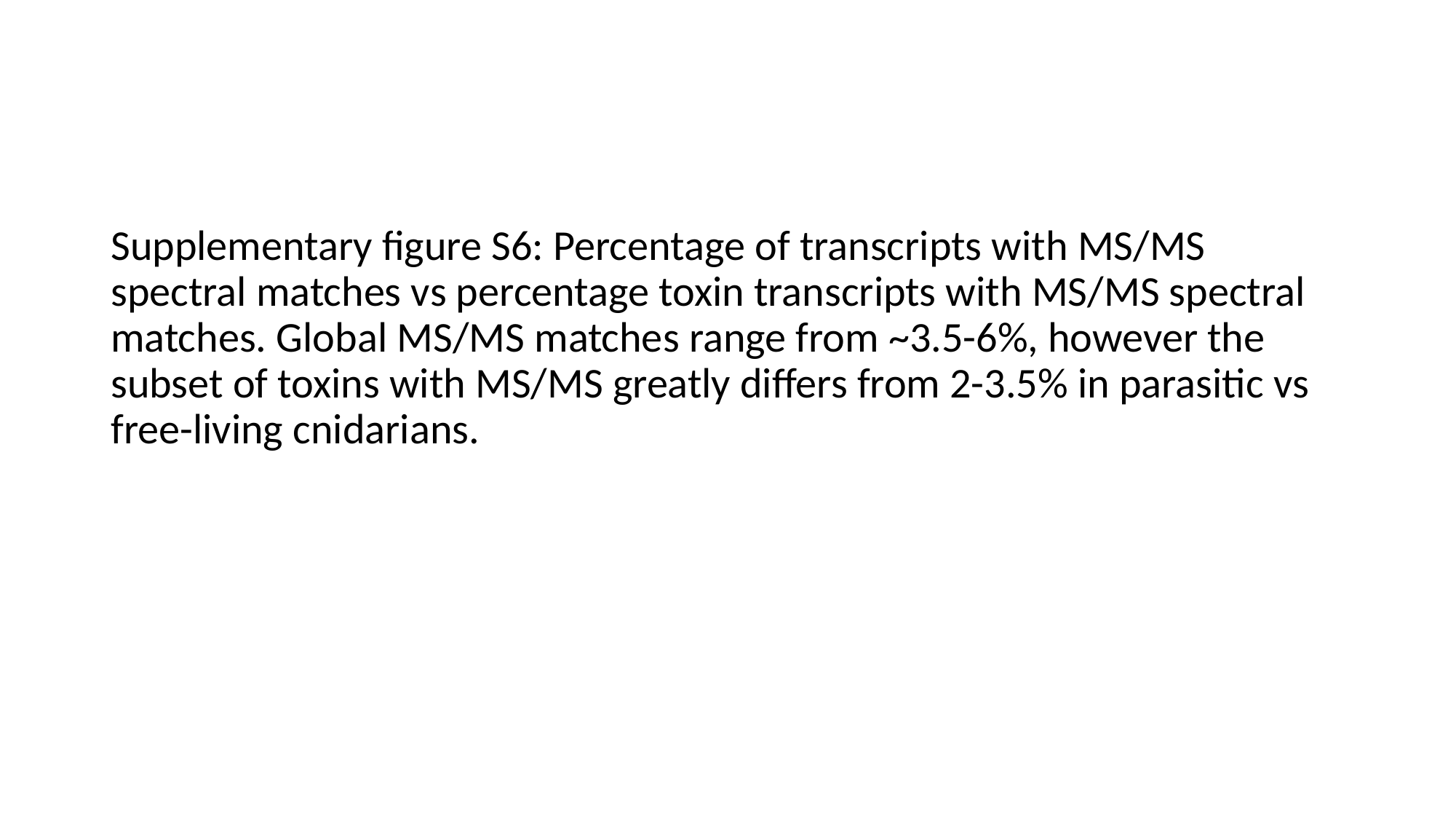

Supplementary figure S6: Percentage of transcripts with MS/MS spectral matches vs percentage toxin transcripts with MS/MS spectral matches. Global MS/MS matches range from ~3.5-6%, however the subset of toxins with MS/MS greatly differs from 2-3.5% in parasitic vs free-living cnidarians.
