# Supplementary figures and images for: Recruitment of toxin-like proteins with ancestral venom function supports endoparasitic lifestyles of Myxozoa
Source: PeerJ. 2021 Apr 26;9:e11208. doi: 10.7717/peerj.11208 (PMC8083181; doi:10.7717/peerj.11208)

Tree scale: 0.1

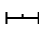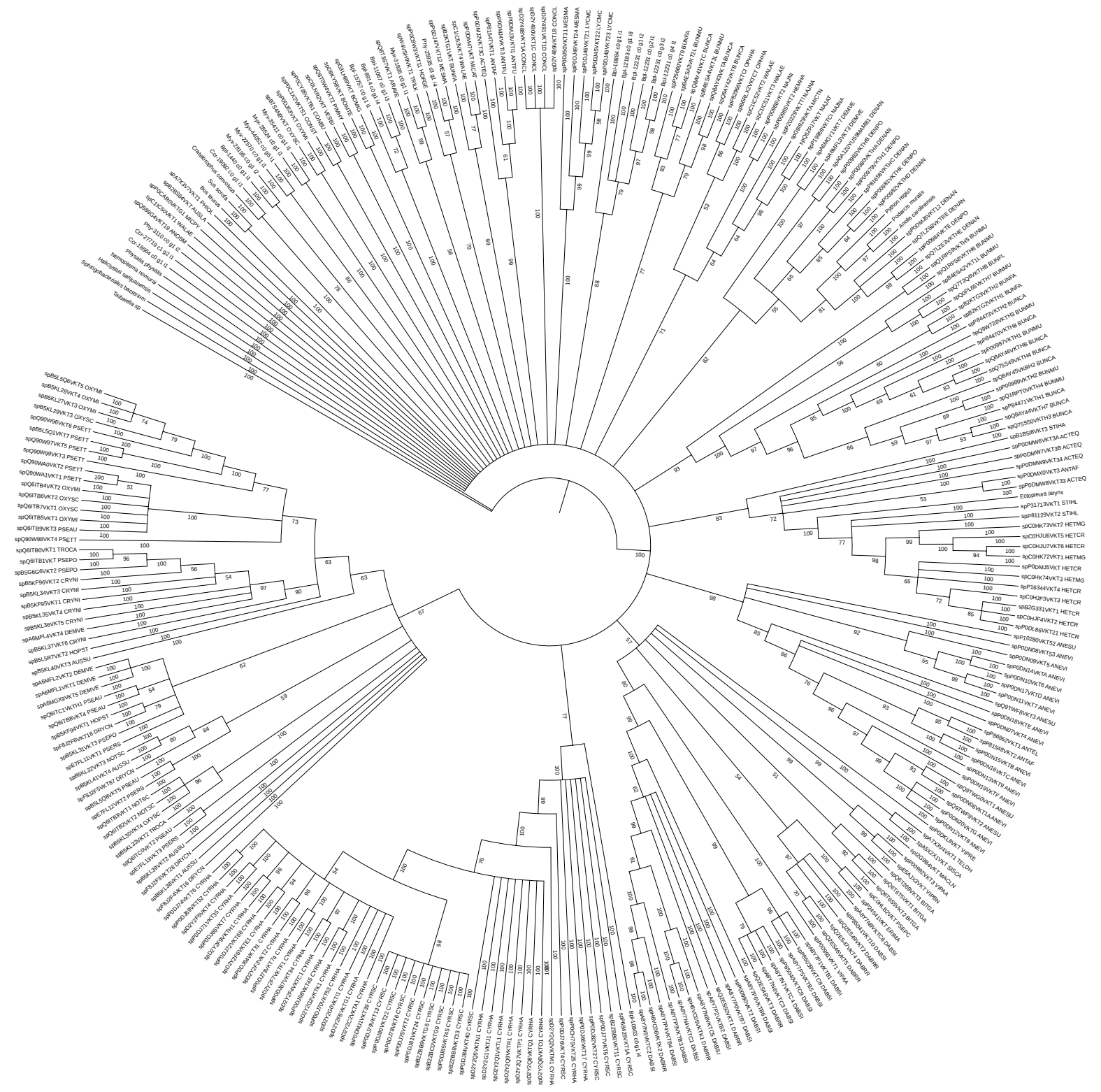

Supplement: Supplemental Information 9 — Phylogenetic tree of the Kunitz-type venom gene family. The tree was constructed with Bayesian inference using MrBayes version 3.2 based on amino acid sequences. Numbers in the nodes show posterior probability values. [file peerj-09-11208-s009.pdf]

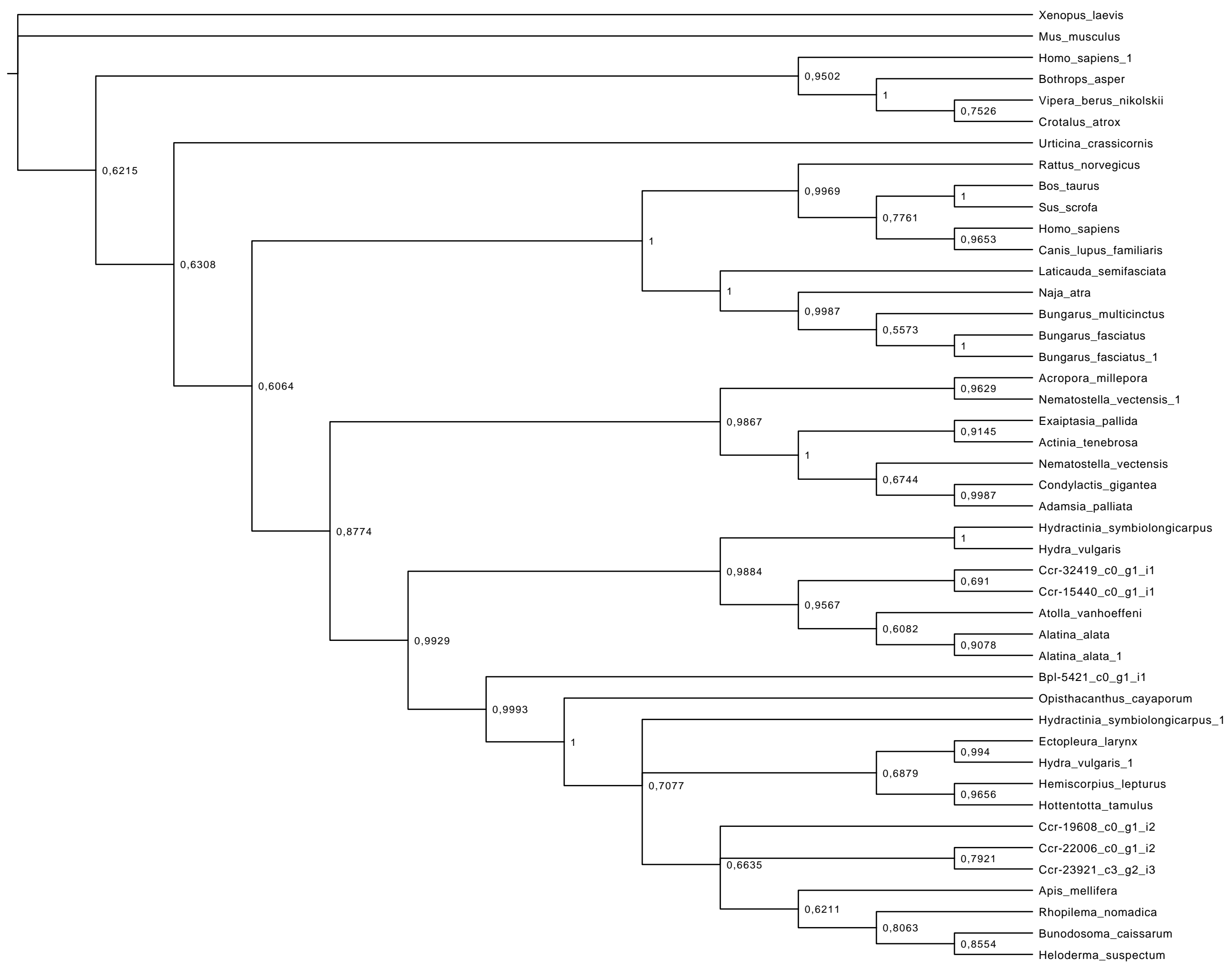

Supplement: Supplemental Information 10 — The tree was constructed with Bayesian inference using MrBayes version 3.2 based on amino acid sequences. Numbers in the nodes show posterior probability values. [file peerj-09-11208-s010.pdf]

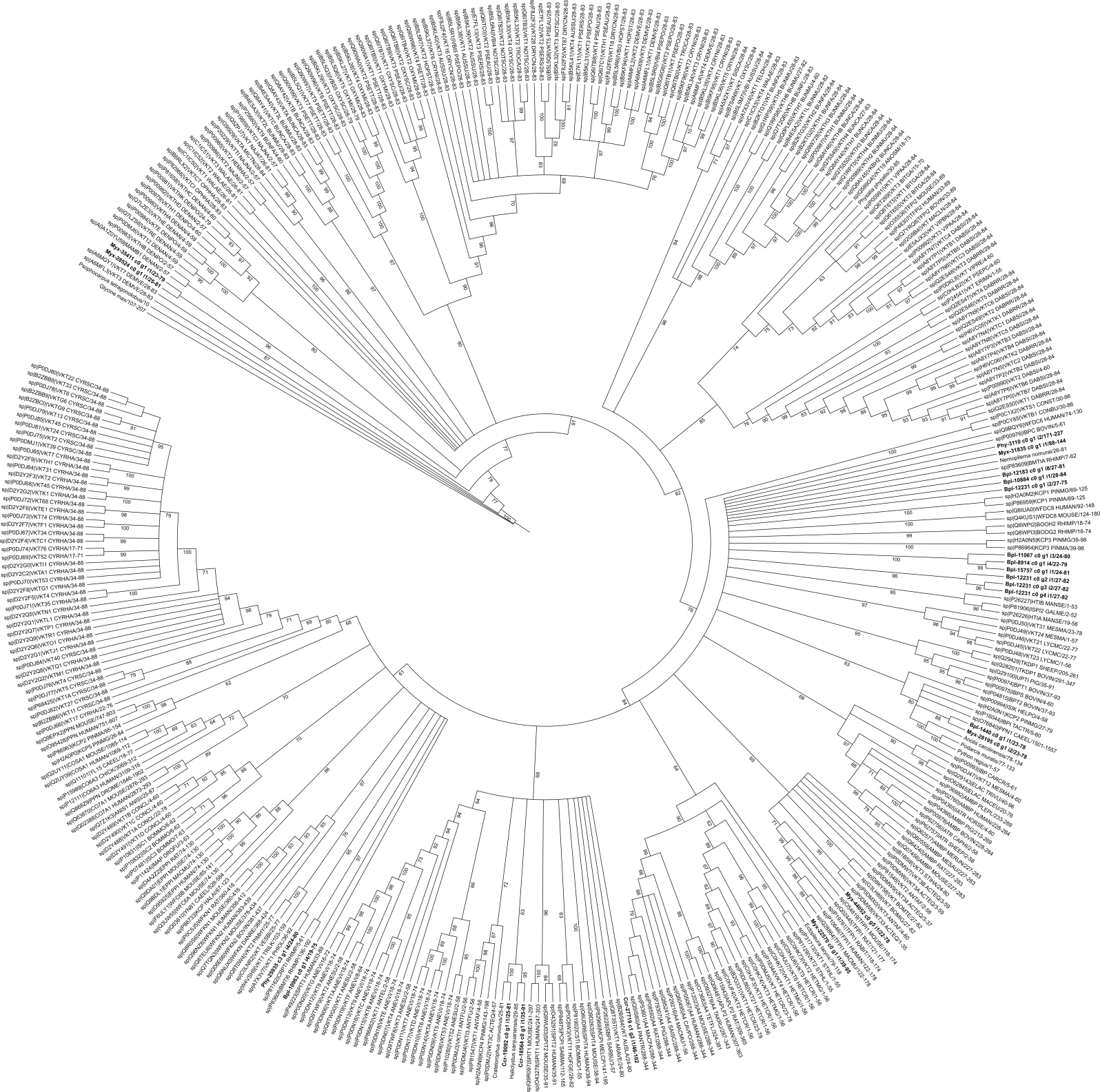

Supplement: Supplemental Information 11 [file peerj-09-11208-s011.pdf]

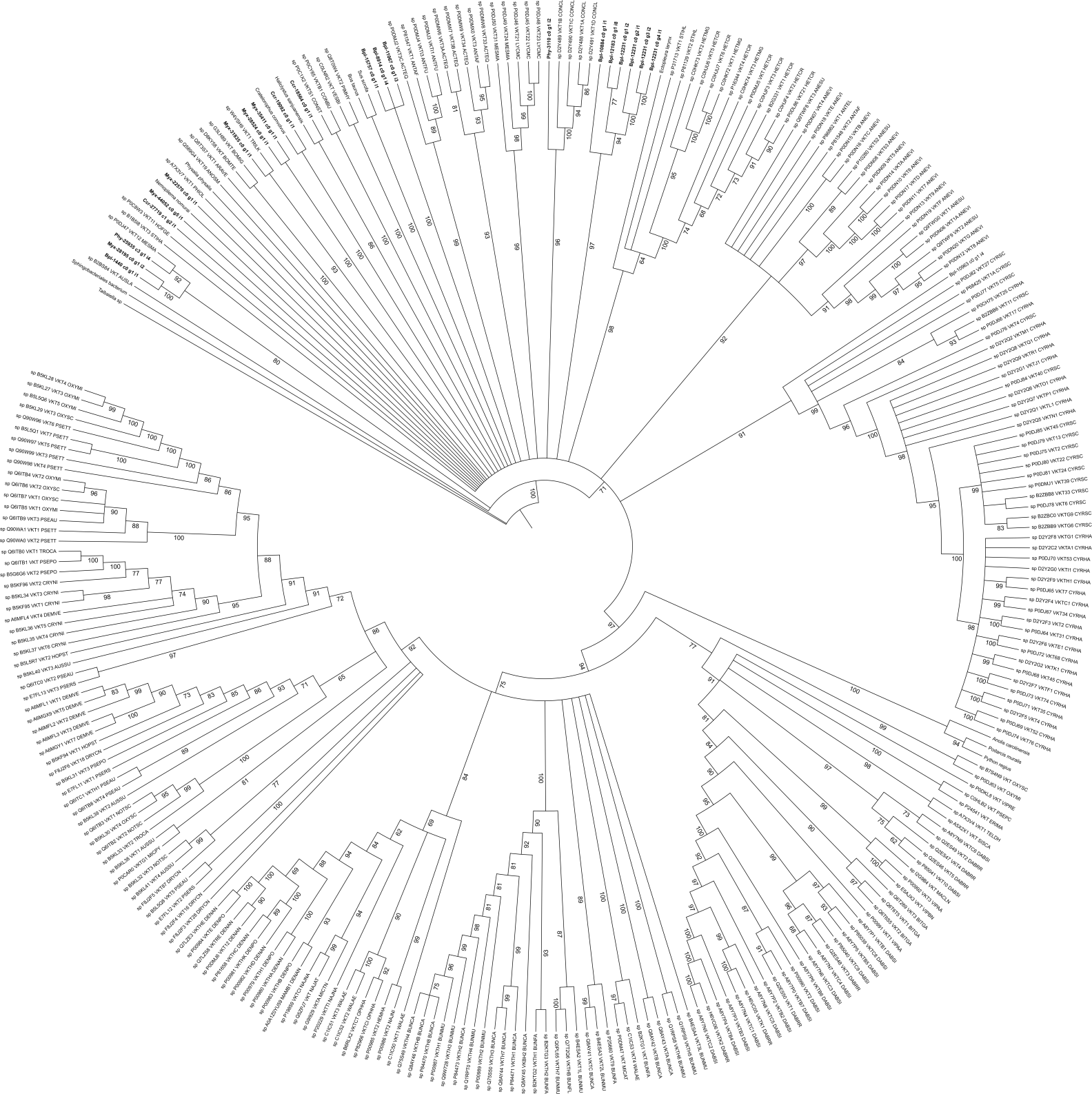

Supplement: Supplemental Information 12 [file peerj-09-11208-s012.pdf]
