# Supplementary material for: Recruitment of toxin-like proteins with ancestral venom function supports endoparasitic lifestyles of Myxozoa
Source: PeerJ. 2021 Apr 26;9:e11208. doi: 10.7717/peerj.11208 (PMC8083181; doi:10.7717/peerj.11208)
Supplement: Supplemental Information 15 [file peerj-09-11208-s015.docx]

| **Species** | **Codes UNIPROT/GENBANK** |
| --- | --- |
| **Outgroup** |  |
| Taibaiella sp | A0A3N7A9E1 |
| Sphingobacteriales bacterium | A0A4V1SFJ6 |
| **Ingroup** |  |
| Anolis carolinensis | H9GSG3 |
| Bos taurus | P00974 |
| Podarcis muralis | XP_028591501.1 |
| Python regius | A0A098LWR4 |
| Sus scrofa | Q29100 |
| Trittame loki (Brush-footed trapdoor spider) | W4VSH9 |
| Bungarus multicinctus (Many-banded krait) | Q9W728 |
| Anemonia sulcata (Mediterranean snakelocks sea anemone) | Q9TWG0 |
| Anemonia sulcata (Mediterranean snakelocks sea anemone) | Q9TWF9 |
| Anemonia sulcata (Mediterranean snakelocks sea anemone) | Q9TWF8 |
| Pseudonaja textilis textilis (Eastern brown snake) | Q90WA1 |
| Pseudonaja textilis textilis (Eastern brown snake) | Q90WA0 |
| Pseudonaja textilis textilis (Eastern brown snake) | Q90W99 |
| Pseudonaja textilis textilis (Eastern brown snake) | Q90W98 |
| Pseudonaja textilis textilis (Eastern brown snake) | Q90W97 |
| Pseudonaja textilis textilis (Eastern brown snake) | Q90W96 |
| Araneus ventricosus (Orbweaver spider) (Epeira ventricosa) | Q8T3S7 |
| Pimpla hypochondriaca (Parasitoid wasp) | Q8T0W4 |
| Bungarus candidus (Malayan krait) | Q8AY46 |
| Bungarus candidus (Malayan krait) | Q8AY45 |
| Bungarus candidus (Malayan krait) | Q8AY44 |
| Bungarus candidus (Malayan krait) | Q8AY43 |
| Bungarus candidus (Malayan krait) | Q8AY42 |
| Bungarus candidus (Malayan krait) | Q8AY41 |
| Bungarus flaviceps flaviceps (Red-headed krait) | Q7T2Q6 |
| Dendroaspis angusticeps (Eastern green mamba) (Naja angusticeps) | Q7LZS8 |
| Dendroaspis angusticeps (Eastern green mamba) (Naja angusticeps) | Q7LZE3 |
| Bungarus candidus (Malayan krait) | Q75S50 |
| Bungarus candidus (Malayan krait) | Q75S49 |
| Bitis gabonica (Gaboon adder) (Gaboon viper) | Q6T6T5 |
| Bitis gabonica (Gaboon adder) (Gaboon viper) | Q6T6S5 |
| Bitis gabonica (Gaboon adder) (Gaboon viper) | Q6T269 |
| Pseudechis australis (Mulga snake) (King brown snake) | Q6ITC1 |
| Pseudechis australis (Mulga snake) (King brown snake) | Q6ITC0 |
| Pseudechis australis (Mulga snake) (King brown snake) | Q6ITB9 |
| Pseudechis australis (Mulga snake) (King brown snake) | Q6ITB8 |
| Oxyuranus scutellatus scutellatus (Australian taipan) (Coastal taipan) | Q6ITB7 |
| Oxyuranus scutellatus scutellatus (Australian taipan) (Coastal taipan) | Q6ITB6 |
| Oxyuranus microlepidotus (Inland taipan) (Diemenia microlepidota) | Q6ITB5 |
| Oxyuranus microlepidotus (Inland taipan) (Diemenia microlepidota) | Q6ITB4 |
| Notechis scutatus scutatus (Mainland tiger snake) (Common tiger snake) | Q6ITB3 |
| Notechis scutatus scutatus (Mainland tiger snake) (Common tiger snake) | Q6ITB2 |
| Pseudechis porphyriacus (Red-bellied black snake) | Q6ITB1 |
| Tropidechis carinatus (Australian rough-scaled snake) | Q6ITB0 |
| Naja atra (Chinese cobra) | Q5ZPJ7 |
| Anoplius samariensis (Solitary wasp) | Q589G4 |
| Daboia russelii (Russel's viper) (Vipera russelii) | Q2ES50 |
| Daboia russelii (Russel's viper) (Vipera russelii) | Q2ES49 |
| Daboia russelii (Russel's viper) (Vipera russelii) | Q2ES48 |
| Daboia russelii (Russel's viper) (Vipera russelii) | Q2ES47 |
| Daboia russelii (Russel's viper) (Vipera russelii) | Q2ES46 |
| Bungarus multicinctus (Many-banded krait) | Q1RPT0 |
| Bungarus multicinctus (Many-banded krait) | Q1RPS9 |
| Bungarus multicinctus (Many-banded krait) | Q1RPS8 |
| Bungarus multicinctus (Many-banded krait) | Q0PL65 |
| Anthopleura elegantissima (Green aggregating anemone) (Actinia elegantissima) | P86862 |
| Daboia siamensis (Eastern Russel's viper) (Daboia russelii siamensis) | P85041 |
| Daboia siamensis (Eastern Russel's viper) (Daboia russelii siamensis) | P85040 |
| Daboia siamensis (Eastern Russel's viper) (Daboia russelii siamensis) | P85039 |
| Bungarus candidus (Malayan krait) | P84473 |
| Bungarus candidus (Malayan krait) | P84471 |
| Bungarus candidus (Malayan krait) | P84470 |
| Ophiophagus hannah (King cobra) (Naja hannah) | P82966 |
| Dendroaspis angusticeps (Eastern green mamba) (Naja angusticeps) | P81658 |
| Anthopleura aff. xanthogrammica (Sea anemone) | P81548 |
| Anthopleura aff. xanthogrammica (Sea anemone) | P81547 |
| Stichodactyla helianthus (Sun anemone) (Stoichactis helianthus) | P81129 |
| Cyriopagopus schmidti (Chinese bird spider) (Haplopelma schmidti) | P68425 |
| Stichodactyla helianthus (Sun anemone) (Stoichactis helianthus) | P31713 |
| Bungarus fasciatus (Banded krait) (Pseudoboa fasciata) | P25660 |
| Eristicophis macmahoni (Leaf-nosed viper) | P24541 |
| Naja naja (Indian cobra) | P20229 |
| Naja naja (Indian cobra) | P19859 |
| Heteractis crispa (Leathery sea anemone) (Radianthus macrodactylus) | P16344 |
| Anemonia sulcata (Mediterranean snakelocks sea anemone) | P10280 |
| Anemonia viridis (Snakelocks anemone) | P0DN20 |
| Anemonia viridis (Snakelocks anemone) | P0DN19 |
| Anemonia viridis (Snakelocks anemone) | P0DN18 |
| Anemonia viridis (Snakelocks anemone) | P0DN17 |
| Anemonia viridis (Snakelocks anemone) | P0DN16 |
| Anemonia viridis (Snakelocks anemone) | P0DN15 |
| Anemonia viridis (Snakelocks anemone) | P0DN14 |
| Anemonia viridis (Snakelocks anemone) | P0DN13 |
| Anemonia viridis (Snakelocks anemone) | P0DN12 |
| Anemonia viridis (Snakelocks anemone) | P0DN11 |
| Anemonia viridis (Snakelocks anemone) | P0DN10 |
| Anemonia viridis (Snakelocks anemone) | P0DN09 |
| Anemonia viridis (Snakelocks anemone) | P0DN08 |
| Anemonia viridis (Snakelocks anemone) | P0DN07 |
| Anemonia viridis (Snakelocks anemone) | P0DN06 |
| Anthopleura aff. xanthogrammica (Sea anemone) | P0DMX0 |
| Actinia equina (Beadlet anemone) | P0DMW9 |
| Actinia equina (Beadlet anemone) | P0DMW8 |
| Actinia equina (Beadlet anemone) | P0DMW7 |
| Actinia equina (Beadlet anemone) | P0DMW6 |
| Dendroaspis angusticeps (Eastern green mamba) (Naja angusticeps) | P0DMJ6 |
| Heteractis crispa (Leathery sea anemone) (Radianthus macrodactylus) | P0DMJ5 |
| Anthopleura fuscoviridis (Sea anemone) | P0DMJ4 |
| Anthopleura fuscoviridis (Sea anemone) | P0DMJ3 |
| Actinia equina (Beadlet anemone) | P0DMJ2 |
| Cyriopagopus schmidti (Chinese bird spider) (Haplopelma schmidti) | P0DMJ1 |
| Micrurus altirostris (Uruguayan coral snake) (Elaps altirostris) | P0DM47 |
| Heteractis crispa (Leathery sea anemone) (Radianthus macrodactylus) | P0DL86 |
| Vipera renardi (Steppe viper) (Vipera ursinii renardi) | P0DKL8 |
| Cyriopagopus schmidti (Chinese bird spider) (Haplopelma schmidti) | P0DJ85 |
| Cyriopagopus schmidti (Chinese bird spider) (Haplopelma schmidti) | P0DJ84 |
| Cyriopagopus schmidti (Chinese bird spider) (Haplopelma schmidti) | P0DJ82 |
| Cyriopagopus schmidti (Chinese bird spider) (Haplopelma schmidti) | P0DJ81 |
| Cyriopagopus schmidti (Chinese bird spider) (Haplopelma schmidti) | P0DJ80 |
| Cyriopagopus schmidti (Chinese bird spider) (Haplopelma schmidti) | P0DJ79 |
| Cyriopagopus schmidti (Chinese bird spider) (Haplopelma schmidti) | P0DJ78 |
| Cyriopagopus schmidti (Chinese bird spider) (Haplopelma schmidti) | P0DJ77 |
| Cyriopagopus schmidti (Chinese bird spider) (Haplopelma schmidti) | P0DJ76 |
| Cyriopagopus schmidti (Chinese bird spider) (Haplopelma schmidti) | P0DJ75 |
| Cyriopagopus hainanus (Chinese bird spider) (Haplopelma hainanum) | P0DJ74 |
| Cyriopagopus hainanus (Chinese bird spider) (Haplopelma hainanum) | P0DJ73 |
| Cyriopagopus hainanus (Chinese bird spider) (Haplopelma hainanum) | P0DJ72 |
| Cyriopagopus hainanus (Chinese bird spider) (Haplopelma hainanum) | P0DJ71 |
| Cyriopagopus hainanus (Chinese bird spider) (Haplopelma hainanum) | P0DJ70 |
| Cyriopagopus hainanus (Chinese bird spider) (Haplopelma hainanum) | P0DJ69 |
| Cyriopagopus hainanus (Chinese bird spider) (Haplopelma hainanum) | P0DJ68 |
| Cyriopagopus hainanus (Chinese bird spider) (Haplopelma hainanum) | P0DJ67 |
| Cyriopagopus hainanus (Chinese bird spider) (Haplopelma hainanum) | P0DJ66 |
| Cyriopagopus hainanus (Chinese bird spider) (Haplopelma hainanum) | P0DJ65 |
| Cyriopagopus hainanus (Chinese bird spider) (Haplopelma hainanum) | P0DJ64 |
| Oxyuranus microlepidotus (Inland taipan) (Diemenia microlepidota) | P0DJ63 |
| Mesobuthus martensii (Manchurian scorpion) (Buthus martensii) | P0DJ50 |
| Mesobuthus martensii (Manchurian scorpion) (Buthus martensii) | P0DJ49 |
| Lychas mucronatus (Chinese swimming scorpion) | P0DJ48 |
| Mesobuthus martensii (Manchurian scorpion) (Buthus martensii) | P0DJ47 |
| Lychas mucronatus (Chinese swimming scorpion) | P0DJ46 |
| Lychas mucronatus (Chinese swimming scorpion) | P0DJ45 |
| Conus bullatus (Bubble cone) | P0CY85 |
| Cyriopagopus hainanus (Chinese bird spider) (Haplopelma hainanum) | P0CH75 |
| Micrurus pyrrhocryptus (Coral snake) | P0CAR0 |
| Hoffmannihadrurus gertschi (Scorpion) (Hadrurus gertschi) | P0C8W3 |
| Conus striatus (Striated cone) | P0C1X2 |
| Vipera ammodytes ammodytes (Western sand viper) | P00992 |
| Vipera ammodytes ammodytes (Western sand viper) | P00991 |
| Daboia siamensis (Eastern Russel's viper) (Daboia russelii siamensis) | P00990 |
| Bungarus multicinctus (Many-banded krait) | P00989 |
| Bungarus multicinctus (Many-banded krait) | P00987 |
| Naja nivea (Cape cobra) (Coluber niveus) | P00986 |
| Hemachatus haemachatus (Rinkhals) (Sepedon haemachatus) | P00985 |
| Dendroaspis polylepis polylepis (Black mamba) | P00984 |
| Dendroaspis polylepis polylepis (Black mamba) | P00983 |
| Dendroaspis angusticeps (Eastern green mamba) (Naja angusticeps) | P00982 |
| Dendroaspis polylepis polylepis (Black mamba) | P00981 |
| Dendroaspis angusticeps (Eastern green mamba) (Naja angusticeps) | P00980 |
| Dendroaspis polylepis polylepis (Black mamba) | P00979 |
| Macrovipera lebetina transmediterranea (Blunt-nosed viper) (Vipera lebetina transmediterranea) | I2G9B4 |
| Daboia russelii (Russel's viper) (Vipera russelii) | H6VC06 |
| Daboia russelii (Russel's viper) (Vipera russelii) | H6VC05 |
| Micrurus tener tener (Texas coral snake) | G9I929 |
| Bombus ignitus (Bumblebee) | G3LH89 |
| Drysdalia coronoides (White-lipped snake) (Hoplocephalus coronoides) | F8J2F6 |
| Drysdalia coronoides (White-lipped snake) (Hoplocephalus coronoides) | F8J2F5 |
| Drysdalia coronoides (White-lipped snake) (Hoplocephalus coronoides) | F8J2F4 |
| Drysdalia coronoides (White-lipped snake) (Hoplocephalus coronoides) | F8J2F3 |
| Pseudechis rossignolii (Papuan pigmy mulga snake) | E7FL13 |
| Pseudechis rossignolii (Papuan pigmy mulga snake) | E7FL12 |
| Pseudechis rossignolii (Papuan pigmy mulga snake) | E7FL11 |
| Vipera berus nikolskii (Nikolsky's adder) (Vipera nikolskii) | E5AJX3 |
| Bombus terrestris (Buff-tailed bumblebee) (Apis terrestris) | D8KY58 |
| Californiconus californicus (California cone) (Conus californicus) | D2Y491 |
| Californiconus californicus (California cone) (Conus californicus) | D2Y490 |
| Californiconus californicus (California cone) (Conus californicus) | D2Y489 |
| Californiconus californicus (California cone) (Conus californicus) | D2Y488 |
| Cyriopagopus hainanus (Chinese bird spider) (Haplopelma hainanum) | D2Y2Q9 |
| Cyriopagopus hainanus (Chinese bird spider) (Haplopelma hainanum) | D2Y2Q8 |
| Cyriopagopus hainanus (Chinese bird spider) (Haplopelma hainanum) | D2Y2Q7 |
| Cyriopagopus hainanus (Chinese bird spider) (Haplopelma hainanum) | D2Y2Q6 |
| Cyriopagopus hainanus (Chinese bird spider) (Haplopelma hainanum) | D2Y2Q5 |
| Cyriopagopus hainanus (Chinese bird spider) (Haplopelma hainanum) | D2Y2Q2 |
| Cyriopagopus hainanus (Chinese bird spider) (Haplopelma hainanum) | D2Y2Q1 |
| Cyriopagopus hainanus (Chinese bird spider) (Haplopelma hainanum) | D2Y2G2 |
| Cyriopagopus hainanus (Chinese bird spider) (Haplopelma hainanum) | D2Y2G1 |
| Cyriopagopus hainanus (Chinese bird spider) (Haplopelma hainanum) | D2Y2G0 |
| Cyriopagopus hainanus (Chinese bird spider) (Haplopelma hainanum) | D2Y2F9 |
| Cyriopagopus hainanus (Chinese bird spider) (Haplopelma hainanum) | D2Y2F8 |
| Cyriopagopus hainanus (Chinese bird spider) (Haplopelma hainanum) | D2Y2F7 |
| Cyriopagopus hainanus (Chinese bird spider) (Haplopelma hainanum) | D2Y2F6 |
| Cyriopagopus hainanus (Chinese bird spider) (Haplopelma hainanum) | D2Y2F5 |
| Cyriopagopus hainanus (Chinese bird spider) (Haplopelma hainanum) | D2Y2F4 |
| Cyriopagopus hainanus (Chinese bird spider) (Haplopelma hainanum) | D2Y2F3 |
| Cyriopagopus hainanus (Chinese bird spider) (Haplopelma hainanum) | D2Y2C2 |
| Walterinnesia aegyptia (Desert black snake) | C1IC53 |
| Walterinnesia aegyptia (Desert black snake) | C1IC52 |
| Walterinnesia aegyptia (Desert black snake) | C1IC51 |
| Walterinnesia aegyptia (Desert black snake) | C1IC50 |
| Vespa bicolor (Black shield wasp) | C0LNR2 |
| Pseudocerastes persicus (Persian horned viper) (False horned viper) | C0HLB2 |
| Heteractis magnifica (Magnificent sea anemone) (Radianthus magnifica) | C0HK74 |
| Heteractis magnifica (Magnificent sea anemone) (Radianthus magnifica) | C0HK73 |
| Heteractis magnifica (Magnificent sea anemone) (Radianthus magnifica) | C0HK72 |
| Heteractis crispa (Leathery sea anemone) (Radianthus macrodactylus) | C0HJU7 |
| Heteractis crispa (Leathery sea anemone) (Radianthus macrodactylus) | C0HJU6 |
| Heteractis crispa (Leathery sea anemone) (Radianthus macrodactylus) | C0HJF4 |
| Heteractis crispa (Leathery sea anemone) (Radianthus macrodactylus) | C0HJF3 |
| Oxyuranus scutellatus scutellatus (Australian taipan) (Coastal taipan) | B7S4N9 |
| Ophiophagus hannah (King cobra) (Naja hannah) | B6RLX2 |
| Hoplocephalus stephensii (Stephens' banded snake) | B5L5R7 |
| Pseudechis australis (Mulga snake) (King brown snake) | B5L5Q8 |
| Oxyuranus microlepidotus (Inland taipan) (Diemenia microlepidota) | B5L5Q6 |
| Pseudonaja textilis textilis (Eastern brown snake) | B5L5Q1 |
| Austrelaps superbus (Lowland copperhead snake) (Hoplocephalus superbus) | B5KL41 |
| Austrelaps superbus (Lowland copperhead snake) (Hoplocephalus superbus) | B5KL40 |
| Austrelaps superbus (Lowland copperhead snake) (Hoplocephalus superbus) | B5KL39 |
| Austrelaps superbus (Lowland copperhead snake) (Hoplocephalus superbus) | B5KL38 |
| Cryptophis nigrescens (Eastern small-eyed snake) (Rhinoplocephalus nigrescens) | B5KL37 |
| Cryptophis nigrescens (Eastern small-eyed snake) (Rhinoplocephalus nigrescens) | B5KL36 |
| Cryptophis nigrescens (Eastern small-eyed snake) (Rhinoplocephalus nigrescens) | B5KL35 |
| Cryptophis nigrescens (Eastern small-eyed snake) (Rhinoplocephalus nigrescens) | B5KL34 |
| Tropidechis carinatus (Australian rough-scaled snake) | B5KL33 |
| Notechis scutatus scutatus (Mainland tiger snake) (Common tiger snake) | B5KL32 |
| Pseudechis porphyriacus (Red-bellied black snake) | B5KL31 |
| Oxyuranus scutellatus scutellatus (Australian taipan) (Coastal taipan) | B5KL30 |
| Oxyuranus scutellatus scutellatus (Australian taipan) (Coastal taipan) | B5KL29 |
| Oxyuranus microlepidotus (Inland taipan) (Diemenia microlepidota) | B5KL28 |
| Oxyuranus microlepidotus (Inland taipan) (Diemenia microlepidota) | B5KL27 |
| Cryptophis nigrescens (Eastern small-eyed snake) (Rhinoplocephalus nigrescens) | B5KF96 |
| Cryptophis nigrescens (Eastern small-eyed snake) (Rhinoplocephalus nigrescens) | B5KF95 |
| Hoplocephalus stephensii (Stephens' banded snake) | B5KF94 |
| Pseudechis porphyriacus (Red-bellied black snake) | B5G6G6 |
| Bungarus multicinctus (Many-banded krait) | B4ESA4 |
| Bungarus multicinctus (Many-banded krait) | B4ESA3 |
| Bungarus multicinctus (Many-banded krait) | B4ESA2 |
| Cyriopagopus schmidti (Chinese bird spider) (Haplopelma schmidti) | B2ZBC0 |
| Cyriopagopus schmidti (Chinese bird spider) (Haplopelma schmidti) | B2ZBB9 |
| Cyriopagopus schmidti (Chinese bird spider) (Haplopelma schmidti) | B2ZBB8 |
| Cyriopagopus schmidti (Chinese bird spider) (Haplopelma schmidti) | B2ZBB6 |
| Bungarus fasciatus (Banded krait) (Pseudoboa fasciata) | B2KTG3 |
| Bungarus fasciatus (Banded krait) (Pseudoboa fasciata) | B2KTG2 |
| Bungarus fasciatus (Banded krait) (Pseudoboa fasciata) | B2KTG1 |
| Heteractis crispa (Leathery sea anemone) (Radianthus macrodactylus) | B2G331 |
| Austrelaps labialis (Pygmy copperhead) (Denisonia superba) | B2BS84 |
| Stichodactyla haddoni (Saddle carpet anemone) (Haddon's sea anemone) | B1B5I8 |
| Daboia siamensis (Eastern Russel's viper) (Daboia russelii siamensis) | A8Y7P6 |
| Daboia siamensis (Eastern Russel's viper) (Daboia russelii siamensis) | A8Y7P5 |
| Daboia siamensis (Eastern Russel's viper) (Daboia russelii siamensis) | A8Y7P4 |
| Daboia siamensis (Eastern Russel's viper) (Daboia russelii siamensis) | A8Y7P3 |
| Daboia siamensis (Eastern Russel's viper) (Daboia russelii siamensis) | A8Y7P2 |
| Daboia siamensis (Eastern Russel's viper) (Daboia russelii siamensis) | A8Y7P1 |
| Daboia siamensis (Eastern Russel's viper) (Daboia russelii siamensis) | A8Y7P0 |
| Daboia siamensis (Eastern Russel's viper) (Daboia russelii siamensis) | A8Y7N9 |
| Daboia siamensis (Eastern Russel's viper) (Daboia russelii siamensis) | A8Y7N8 |
| Daboia siamensis (Eastern Russel's viper) (Daboia russelii siamensis) | A8Y7N7 |
| Daboia siamensis (Eastern Russel's viper) (Daboia russelii siamensis) | A8Y7N6 |
| Daboia siamensis (Eastern Russel's viper) (Daboia russelii siamensis) | A8Y7N5 |
| Daboia siamensis (Eastern Russel's viper) (Daboia russelii siamensis) | A8Y7N4 |
| Philodryas olfersii (Green snake) | A7X3V7 |
| Telescopus dhara (Egyptian catsnake) | A7X3V4 |
| Demansia vestigiata (Lesser black whip snake) (Demansia atra) | A6MGY1 |
| Demansia vestigiata (Lesser black whip snake) (Demansia atra) | A6MGX9 |
| Demansia vestigiata (Lesser black whip snake) (Demansia atra) | A6MFL4 |
| Demansia vestigiata (Lesser black whip snake) (Demansia atra) | A6MFL3 |
| Demansia vestigiata (Lesser black whip snake) (Demansia atra) | A6MFL2 |
| Demansia vestigiata (Lesser black whip snake) (Demansia atra) | A6MFL1 |
| Sistrurus catenatus edwardsii (Desert massasauga) (Crotalophorus edwardsii) | A5X2X1 |
| Dendroaspis angusticeps (Eastern green mamba) (Naja angusticeps) | A0A1Z0YU59 |
