# Supplementary material for: Recruitment of toxin-like proteins with ancestral venom function supports endoparasitic lifestyles of Myxozoa
Source: PeerJ. 2021 Apr 26;9:e11208. doi: 10.7717/peerj.11208 (PMC8083181; doi:10.7717/peerj.11208)
Supplement: Supplemental Information 16 [file peerj-09-11208-s016.docx]

| **Species** | **Codes UNIPROT/GENBANK** |
| --- | --- |
| **Outgroup** |  |
| *Xenopus_laevis* | P41485 |
| **Ingroup** |  |
| *Adamsia_palliata* | Q8WS88 |
| *Apis_mellifera* | P00630 |
| *Bos_taurus* | P00593 |
| *Bothrops_asper* | P24605 |
| *Bungarus_fasciatus* | A6MEY4 |
| Bungarus_fasciatus_1 | Q90WA8 |
| *Bungarus_multicinctus* | P00618 |
| *Bunodosoma_caissarum* | P86780 |
| *Canis_lupus_familiaris* | P06596 |
| *Condylactis_gigantea* | D2X8K2 |
| *Crotalus_atrox* | P00624 |
| *Exaiptasia_pallida* | XP_020900455.1 |
| *Heloderma_suspectum* | P16354 |
| *Hemiscorpius lepturus* | A0A1L4BJ46 |
| *Homo_sapiens* | P04054 |
| *Homo_sapiens_1* | P14555 |
| *Hottentotta_tamulus* | Q6T178 |
| *Hydra_vulgaris* | XP_002157393.1 |
| *Laticauda_semifasciata* | Q8JFG2 |
| *Mus musculus* | Q9QUL3 |
| *Naja_atra* | A4FS04 |
| *Nematostella_vectensis* | A7S5K1 |
| *Nematostella_vectensis_1* | A7S6H4 |
| *Opisthacanthus cayaporum* | P86120 |
| *Rattus_norvegicus* | P04055 |
| *Rhopilema_nomadica* | P43318 |
| *Sus_scrofa* | P00592 |
| *Urticina_crassicornis* | A7LCJ2 |
| *Vipera berus_nikolskii* | Q1RP79 |
