# Supplementary material for: Recruitment of toxin-like proteins with ancestral venom function supports endoparasitic lifestyles of Myxozoa
Source: PeerJ. 2021 Apr 26;9:e11208. doi: 10.7717/peerj.11208 (PMC8083181; doi:10.7717/peerj.11208)
Supplement: Supplemental Information 18 [file peerj-09-11208-s018.docx]

|  | *Buddenbrockia plumatellae* | Mixed Myxosporeans (*Sphaerospora elegans* and *Myxobilatus gasterostei*) | *Calvadosia cruxmelitensis* | *Polypodium hydriforme* |
| --- | --- | --- | --- | --- |
| **Trinity Assembly** | | | | |
| Total transcripts | 74 663 | 157 830 | 142 601 | 149 058 |
| Average contig length | 1355.76 | 773.51 | 941.12 | 953.77 |
| GC percentage | 29.55 | 47.24 | 43.76 | 47.5 |
| **Redundancy and host contamination removal** | | | | |
| Translated (ORF Predictor) | 73 930 | 156 998 | 141 043 | 147 536 |
| NR with CD-HIT | 44 485 | 156 998 | 116 079 | 122 923 |
| Host contaminants | 3 532 | 22 473 | - | 887 |
| Chimeras/Indeterminate seqs | 236 | 46 118 | - | 1 384 |
| NR clean transcriptome | 40 953 | 107 645 | 116 079 | 122 036 |
| **BUSCO gene analysis (complete metazoan gene set = 978)** | | | | |
| Single copy (% of 978) | 270 (27.6%) | 287 (29.3%) | 679 (69.4%) | 565 (57.7%) |
| Duplicate (% of 978) | 387 (39.5%) | 131 (13.3%) | 185 (18.9%) | 279 (28.5%) |
| Fragmented (% of 978) | 51 (5.2%) | 348 (35.6%) | 45 (4.6%) | 45 (4.6%) |
| Missing (% of 978) | 270 (27.6%) | 212 (21.6%) | 69 (7.1%) | 89 (9.1%) |
| Total BUSCO genes found (% of 978) | 708 (72.4%) | 766 (78.3%) | 909 (92.9%) | 889 (90.9%) |
