# Supplementary material for: Recruitment of toxin-like proteins with ancestral venom function supports endoparasitic lifestyles of Myxozoa
Source: PeerJ. 2021 Apr 26;9:e11208. doi: 10.7717/peerj.11208 (PMC8083181; doi:10.7717/peerj.11208)
Supplement: Supplemental Information 23 [file peerj-09-11208-s023.docx]

**Table S10. Putative toxins identified from the proteomes of free-living cnidarians.** Transcripts with putative toxin functions supported by unique MS/MS spectral events are represented here in toxin groups (based on domain presence and homology) from *Calvadosia cruxmelitensis* and *Polypodium hydriforme*. Spectra were matched to transcripts using PEAKS software, percentage of transcripts in each toxin group with matching MS/MS in brackets.

|  | *Calvadosia cruxmelitensis* | *Polypodium hydriforme* |
| --- | --- | --- |
| 5'-nucleotidase | 0 | 2 (11.1%) |
| Aspartic peptidase | 0 | 2 (100%) |
| Conotoxin | 2 (50.0%) | 0 |
| CRISP | 4 (28.6%) | 5 (62.5%) |
| C-type lectin | 2 (20.0%) | 2 (25.0%) |
| Glutaminyl-peptide cyclotransferase | 0 | 2 (66.7%) |
| Glycosyl hydrolase | 3 (16.7%) | 5 (29.4%) |
| Lipase | 1 (12.5%) | 5 (62.5%) |
| Metallopeptidase | 21 (23.1%) | 27 (30.8%) |
| Oxidase | 0 | 6 (100%) |
| Phosphodiesterase | 3 (15.8%) | 0 |
| Phospholipase | 2 (20.0%) | 5 (55.5%) |
| Phospholipase D/Dnase II | 1 (8.3%) | 0 |
| Serine peptidase | 6 (20.7%) | 8 (42.1%) |
| Serine peptidase inhibitor (kunitz-like) | 1 (33.3%) | 0 |
| Venom complement C3 homolog family | 1 (100%) | 0 |
| Other | 14 (23.3%) | 3 (37.5%) |
| No domains | 3 (14.3%) | 5 (33.3%) |
| TOTAL | 64 | 77 |
